# Access to β-Alkylated γ-Functionalized Ketones via Conjugate Additions to Arylideneisoxazol-5-ones and Mo(CO)_6_-Mediated Reductive Cascade Reactions

**DOI:** 10.1021/acsomega.1c07081

**Published:** 2022-03-04

**Authors:** Antonio Macchia, Francesco F. Summa, Guglielmo Monaco, Andreas Eitzinger, Armin R. Ofial, Antonia Di Mola, Antonio Massa

**Affiliations:** † Dipartimento di Chimica e Biologia “A. Zambelli”, Università degli studi di Salerno, Via Giovanni Paolo II, 84084 Fisciano, Salerno, Italy; ‡ Department Chemie, Ludwig-Maximilians-Universität Munchen, 81377 Munchen, Germany

## Abstract

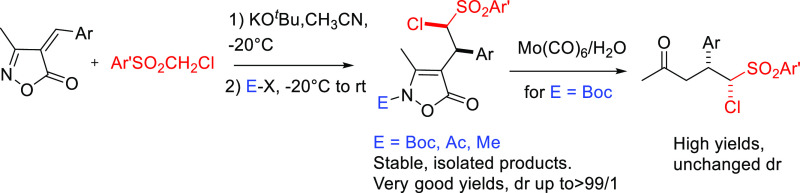

1,4-Conjugate addition of ((chloromethyl)sulfonyl)benzenes to arylideneisoxazol-5-ones, followed by one-pot, N-selective trapping in the presence of electrophiles, was investigated. This strategy led to the synthesis of new, stable N-protected isoxazol-5-ones in good yields and high diastereolectivity. The study of the reactivity of obtained products in the presence of the Mo(CO)_6_/H_2_O system allowed the development of a cascade reaction leading to novel methyl ketones in high yields and unchanged dr bearing an uncommon chloromethinearylsulfonyl end group.

## Introduction

1,4-Conjugate addition of nucleophiles carrying a leaving group (LG) in the α-position like ((chloromethyl)sulfonyl)benzene (PhSO_2_CH_2_Cl) is particularly useful in the development of effective cyclopropanation reactions,^1^ while the isolation of the respective Michael adducts has been rarely accomplished.^2^ The presence of the LG drives the reactivity of this pro-nucleophile in several other domino reactions as typically in vicarious nucleophilic substitutions (VNS reactions) at electron-deficient arenes^3^ or in the formation of oxiranes (Darzens condensation) when combined with carbonyl compounds.^4^ In our recent study, we were able to tune the reactivity of ((chloromethyl)sulfonyl)benzene in the addition to carbonyls with the introduction of a further electrophilic cyano group as in 2-acetylbenzonitriles, which competed with chloride displacement of the alkoxide intermediate, leading to the formation of isoindolin-1-ones instead of oxiranes.^5^

**Figure 1:**
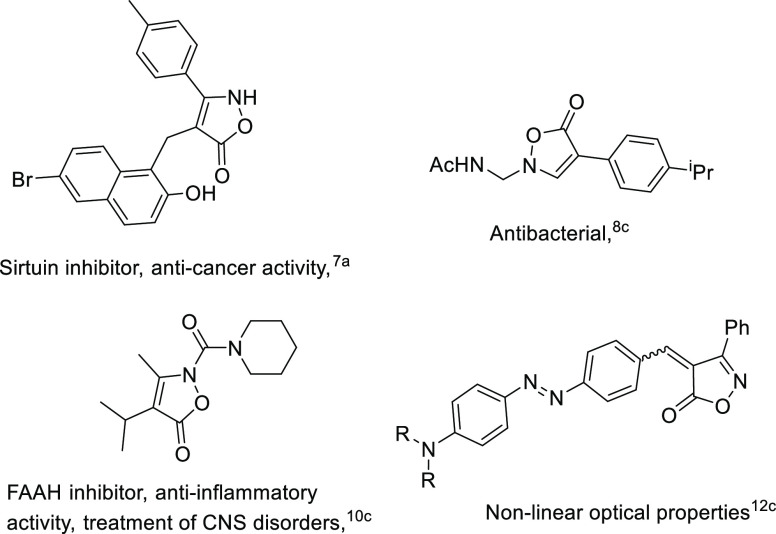
Selected isoxazol-5-ones showing biological and optical properties.

Isoxazol-5-ones are heterocyclic compounds, which are straightforwardly obtained by condensation of hydroxylamine with readily available β-ketoesters. This class of heterocycles gains increasing interest^6^ because of the wide range of biological properties as anti-cancer,^7^ anti-microbial,^8^ anti-obesity,^9^ and anti-inflammatory agents^10^ or as functional materials in non-linear optical^11^ and luminescent probes (see Figure 1 for selected examples).^12^ In addition, the rather labile nature of the N–O bond combined with the unique properties of the isoxazole ring enables the synthesis of numerous classes of diverse acyclic and cyclic compounds under several reaction conditions.^6,13−18^

**Scheme 1:**
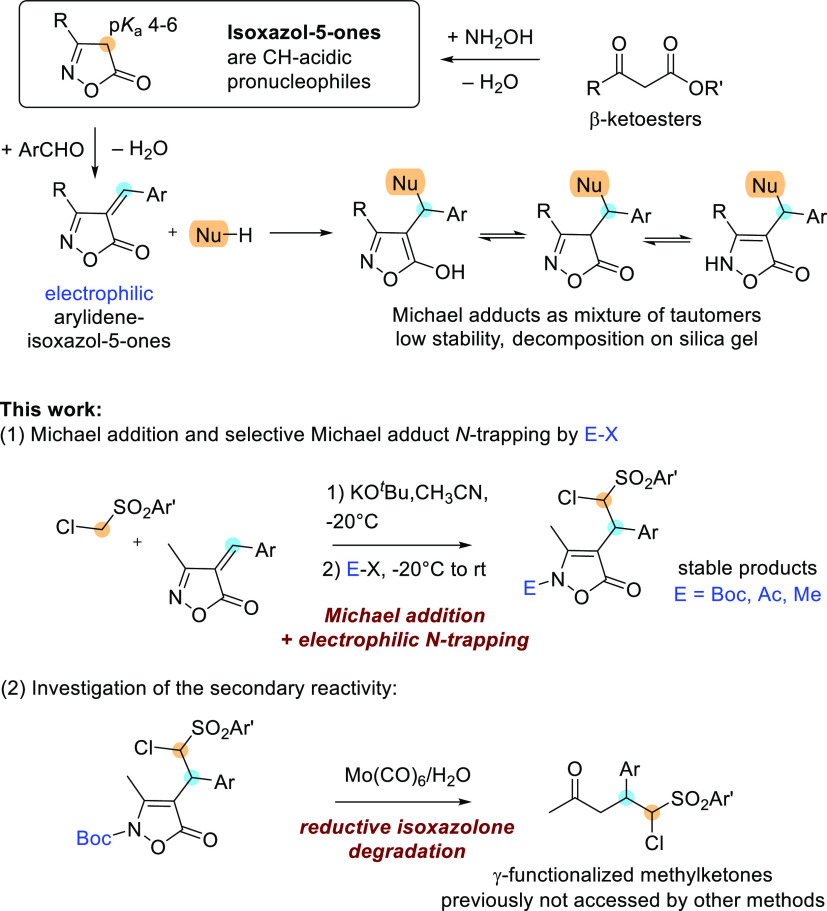
General Reactivity of Isoxazol-5-ones and Present Work

Isoxazol-5-ones are characterized by relatively high acidity at C-4 (p*K*_a_ 4–6),^6a^ and the resulting carbanions find a wide use as nucleophiles,^6a−6d^ which can also be used in condensation reactions with aldehydes to generate electrophilic arylideneisoxazol-5-ones (Scheme 1).^6^

Michael reactions of arylideneisoxazol-5-ones suffer, however, from a relatively limited scope.^6,14^ Tautomerism and scarce stability of the adducts are the main drawbacks (Scheme 1).^6,14^ Non-isolated Michael adducts have been directly transformed into acyclic ketones or alkynes by nitrosative cleavage of the N–O bond in the presence of FeSO_4_/NaNO_2_^13,14^ into diverse heterocyclic compounds using molybdenum reagents^15,16^ or as part of domino reactions in the presence of multifunctional nucleophiles, leading to spirocyclic compounds.^17^ On the other hand, when a reactive electrophilic component was included at the end of 1,4-conjugate addition of malonate diesters to arylideneisoxazol-5-ones, the selective N-trapping of the adduct was achieved in high efficiency, preserving the isoxazol-5-one architecture.^18^ The obtained products showed high stability and were easily purified by standard techniques, while any attempt to purify or isolate the unprotected Michael adducts led to failures.^18^

As part of our research interest in the development of new reactions involving multifunctional electrophiles and nucleophiles, in the present work, we have investigated Michael reactions of arylideneisoxazol-5-ones with ((chloromethyl)sulfonyl)benzenes and the capacity of the obtained adducts to undergo Mo(CO)_6_/H_2_O promoted reductive cleavage of the O−N bond (Scheme 1). After conjugate addition, the enolate intermediate could give intramolecular displacement of the chloride, leading to cyclopropane formation. On the other hand, the N-selective trapping of the enamine form of the adducts by an electrophile E–X should preserve the structure of the chloromethinephenylsulfonyl end group (Scheme 1).

## Results and Discussion

### Conjugate Additions of ((Chloromethyl)sulfonyl)benzene to Arylideneisoxazol-5-ones and Subsequent Trapping with Electrophiles

Intrigued by this possible dualism (see the scheme of Table 1), in a first set of reactions, we investigated the reactivity of the carbanion of ((chloromethyl)sulfonyl)benzene **1** (PhSO_2_CH_2_Cl) quantitatively generated by reaction with KO^*t*^Bu (1 equiv), with 3-methyl-4-benzylideneisoxazol-5-ones **2** in anhydrous acetonitrile. Complete conversion was detected at −20 °C after 4 h of reaction time by thin-layer chromatography (TLC) (Table 1, entry 2), while at rt, we observed a series of unknown decomposition products (entry 1). ^1^H NMR spectroscopic analysis of the reaction mixture, obtained under the conditions of entry 2, in CD_3_CN was inconclusive because the formation of a precipitate affected the spectra. After the evaporation of the solvent, ^1^H NMR analysis in CDCl_3_ gave somewhat better indications, highlighting the disappearance of **2**, the presence of signals compatible with the protonated Michael adduct of **I-3a**, and the lack of the cyclopropyl moiety (Table 1, entry 2). However, every attempt to purify the crude by chromatography led to decomposition together with the isolation of the starting materials, probably due to retro-Michael reaction occurring on silica gel. Therefore, we investigated the possibility to obtain stable products by the addition of electrophiles E–X at the end of the Michael reaction at −20 °C, as previously reported with dimethyl malonate.^18^ Our choice focused on di-*tert*-butyl di-carbonate aiming to a N-selective interception of the **I-3a** intermediate. Under the conditions of entry 3, the sequential reaction allowed the isolation after chromatography of stable *N*-Boc-protected **4a**, bearing an uncommon chloromethinephenylsulfonyl side chain in good yield and excellent dr >94:6. The use of weaker bases, K_2_CO_3_ and Et_3_N, was not effective in promoting the Michael reaction since unreacted starting materials were detected by TLC and ^1^H NMR analysis of the crude materials (entries 4 and 5).

**Table 1:**
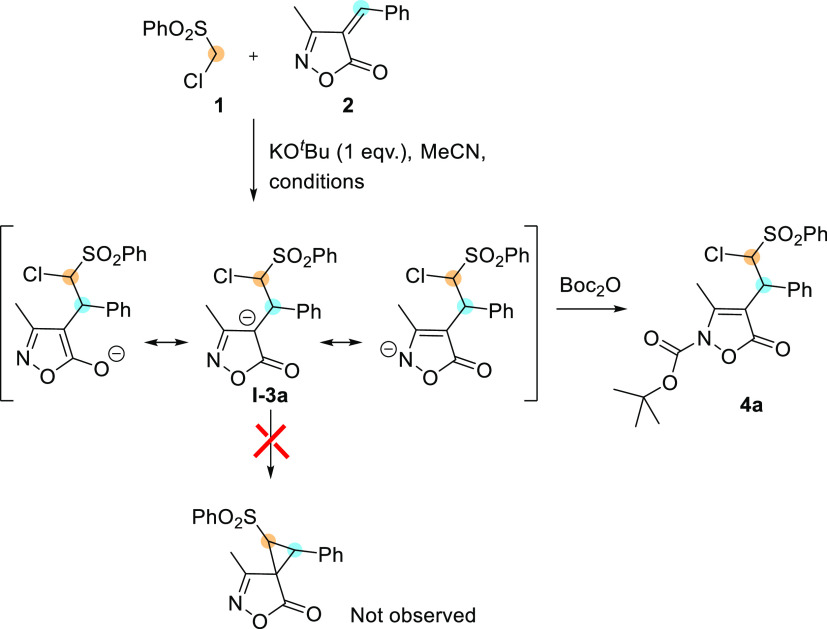
Preliminary Investigation of the Michael Reaction of ((Chloromethyl)sulfonyl)benzene **1** with 3-Methyl-4-benzylideneisoxazol-5-ones **2**

entry	E	*T* (°C)	time (h)	yield (%)
1		r.t.	2	a
2		–20 °C	4	b
3	Boc_2_O	–20 °C	4 + 2^c^	**4a**, 70^d^
4^e^		r.t.	18	no react.
5^f^		r.t.	18	no react.

a Unknown degradation products were detected.

b Starting materials and decomposition products were isolated after chromatography.

c Time addition + protection.

d Isolated yield.

e K_2_CO_3_ was used.

f Et_3_N was used.

Synthetic access to stable, highly functionalized isoxazol-5-ones is a very important aim since this class of compounds shows a wide range of biological activities and interesting optical properties (Figure 1).^6−12^ Therefore, the scope of the sequential reaction was thoroughly analyzed combining different readily available^1a,5^ ((chloromethyl)sulfonyl)benzenes and 3-methyl-4-arylideneisoxazol-5-ones^18^ bearing electron-withdrawing and electron-donating groups on the aromatic rings of both the nucleophiles and electrophiles (Table 2). Apart from di-*tert*-butyl dicarbonate, two other reagents E–X, acetic anhydride and iodomethane, were used in order to investigate if they could lead to products with different substituents on the nitrogen of the heterocyclic ring (Table 2). Based on the data reported in Table 2, the method proved to be effective with all the combinations of substrates, affording in good yields a wide range of new *N*-protected, stable products **4** in the enamine form, demonstrating the efficiency of the electrophilic trapping strategy also in the presence of alkylating or acylating reagents.

**Table 2:**
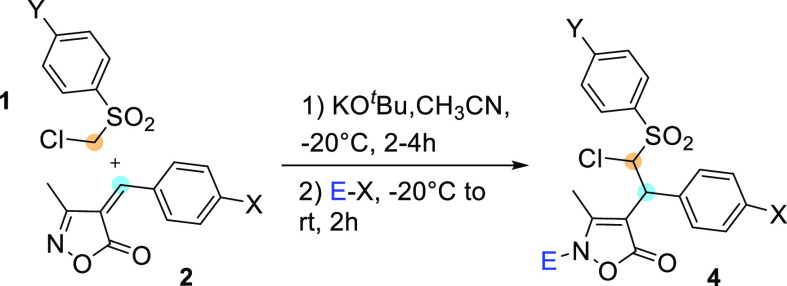

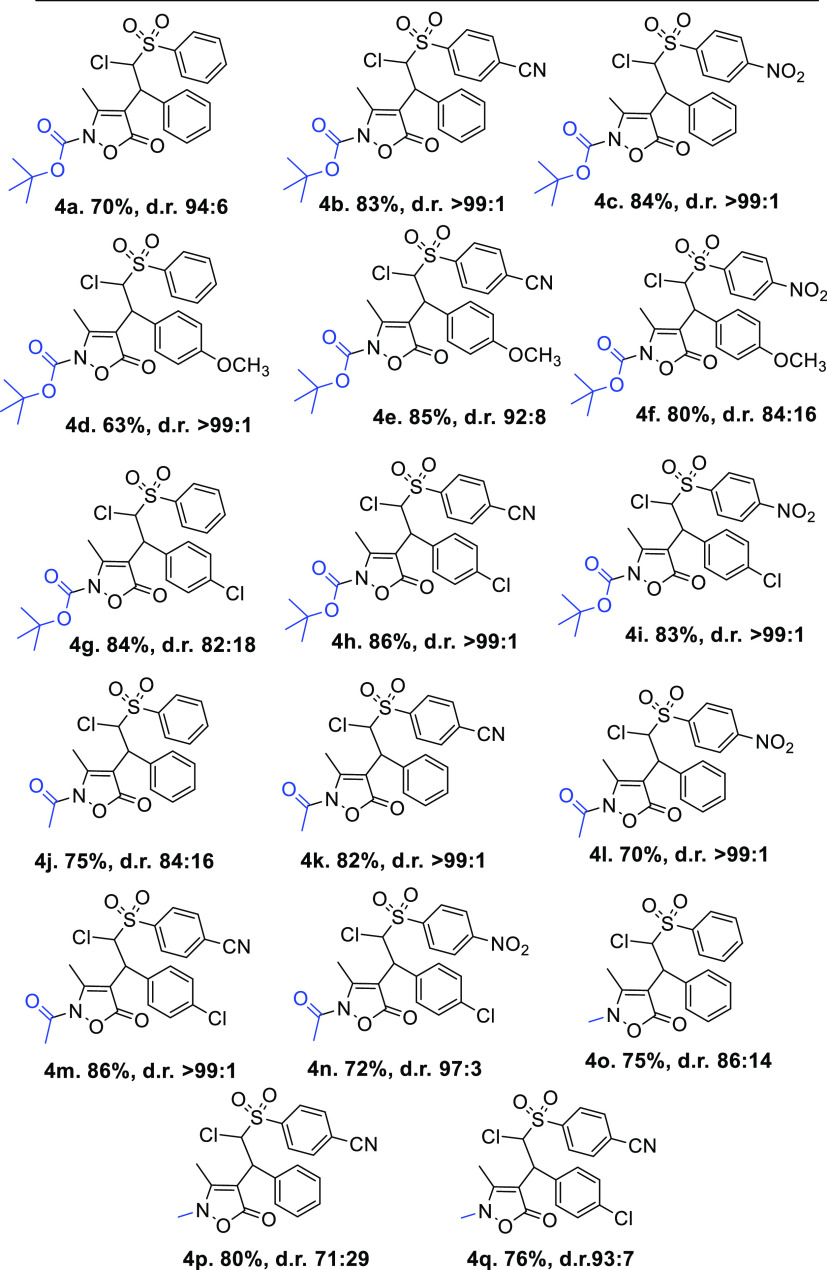
Scope of the Michael Reaction of ((Chloromethyl)sulfonyl)benzenes with 3-Methyl-4-benzylideneisoxazol-5-ones

Excellent diastereomeric ratios, up to >99/1 dr were detected in most of the cases. We have not investigated the mechanism to explain this rather general outcome and the few exceptions, which seem to be independent of the substituents and the type of electrophilic trapping reagents. It is likely that the high diastereoselectivity is the result of kinetic control and in a few cases, epimerization occurs to a certain extent. Since we were not able to prepare single crystals of the compounds obtained, the relative configuration was deduced by correlating the experimental and calculated ^1^H NMR spectra of the products of the further transformation of **4** (see the next section).

### Investigation of the Reactivity of N-protected Isoxazole-5-ones under Reductive Cleavage of the O–N Bond

The obtained products **4** can be particularly useful in further transformations involving the cleavage of the N–O bond which can allow the access to unprecedented compounds. During the last years, there has been an increased interest of academia and industry in molybdenum compounds in organic synthesis.^15^ In particular, molybdenum hexacarbonyl Mo(CO)_6_ has been used in various reactions, namely, C–C bond formation, cyclization, reductions, oxidations, and heterocyclic ring formation^15,19−25^ as well as in the ring cleavage of isoxazole and isooxazoline compounds.^15,16^

In particular, reductive cleavage of the O–N bond of isoxazoles and isoxazolines in the presence of the Mo(CO)_6_/H_2_O system has been used in the synthesis of new heterocycles by further in situ rearrangement of the open intermediates.^15,16^ However, the effect of this system has been scarcely investigated on isoxazol-5-ones derivatives.^25^ After O–N cleavage of **4**, in principle, the supposed formation of enamine or carboxylate groups could lead to competitive intramolecular displacements of chloride to afford five-membered heterocyclic compounds.

In order to explore the reactivity of **4** under reductive cleavage of the O–N bond with the Mo(CO)_6_/H_2_O system, a series of differently protected compounds **4** were subjected to react with Mo(CO)_6_/H_2_O under the conditions of Table 3. *N*-Methyl-enamine derivative **4o** gave decomposition products. *N*-Acetyl-enamine derivative **4j** did not react. Surprisingly, the *N*-Boc-protected **4a** led smoothly to the isolation of an unprecedented β-alkylated γ-functionalized ketone **5a**, which cannot be easily obtained by other methods such as 1,4-conjugate additions of electron-deficient alkene^2^ or by direct β-functionalization of saturated ketones in the presence of Pd catalysts.^26^

**Table 3:**
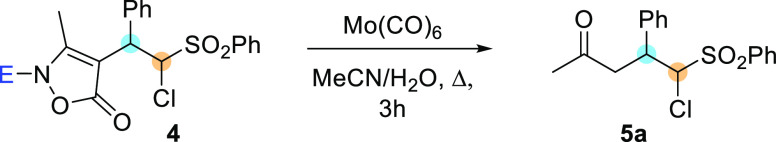
Preliminary Investigation of the Mo(CO)_6_-Mediated Reaction

entry	**4**	E	yield (%)^a^
1	**4o**	Me	decomp.
2	**4j**	Ac	no react.
3	**4a**	Boc	87%

a Isolated yield.

**Scheme 2:**
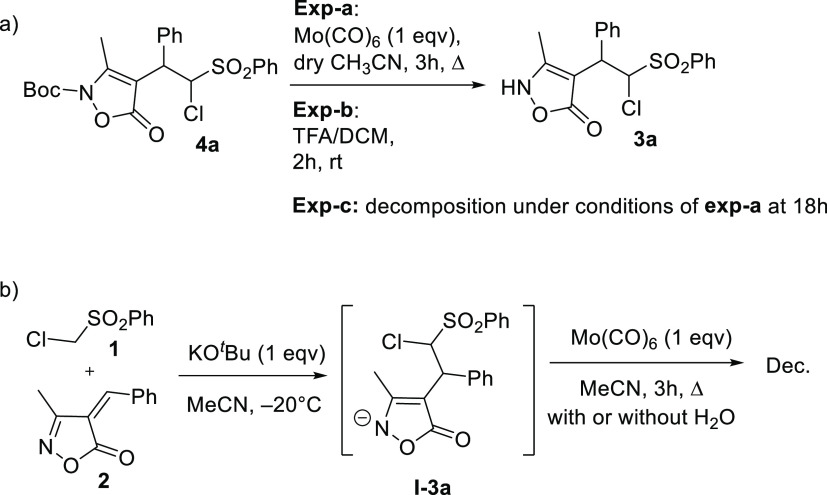
Control Experiments for the Mo(CO)_6_-Mediated Reaction

**Scheme 3:**
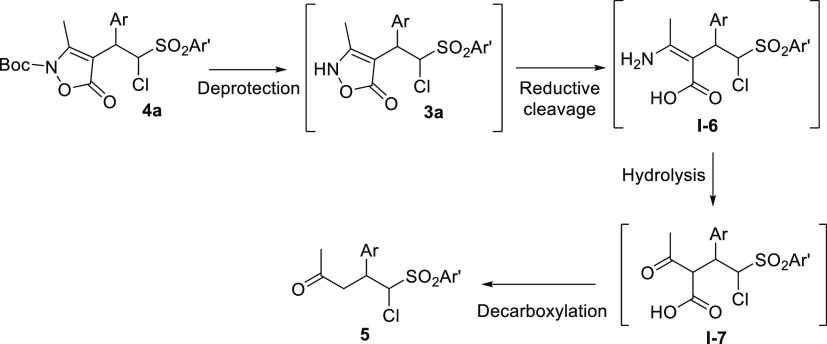
Proposed Steps in the Cascade Reductive Cleavage of the Isozazol-5-ones

As confirmed by a series of control experiments (Scheme 2), the reaction presumably follows the order of Boc-deprotection, cleavage of O–N bond, enamine/imine hydrolysis, and decarboxylation (Scheme 3). The role of water is important since the presence of only Mo(CO)_6_ led to NH-enamine as detected in the ^1^H NMR spectrum of the crude because of Boc-deprotection (Scheme 2a, Exp-a), while a longer reaction time led to decomposition products (Scheme 2a, Exp-c). The unprotected Michael adduct **3a** obtained as crude, according to Scheme 2b (see also Table 1), was subjected to reaction in the presence of Mo(CO)_6_ with or without water. In both the experiments, decomposition products were observed, demonstrating the importance of *N*-Boc-protection to accomplish this cascade reaction. On the other hand, the treatment of **4a** with the TFA/DCM mixture under Boc-deprotection conditions afforded, after aqueous work-up, **3a** and to certain extent **1** and **2** as a consequence of retro-Michael reaction (**3a/1** about 3/1, Scheme 2a, Exp-b). This indicates that Boc-deprotection and acidic conditions are not sufficient to trigger the cleavage of the O–N bond and the following cascade reaction, but Mo(CO)_6_ plays a key role. Based on these considerations, a stepwise mechanism has been proposed, highlighting all the possible intermediates (Scheme 3). After Boc-deprotection, the hydrolysis of the enamine/imine intermediate and decarboxylation of **I-6** or **I-7** are presumably faster than possible isomerization of the double bond and cyclizations,^16a^ preserving the chloromethinephenylsulfonyl moiety. The coordination of the nitrogen in the isoxazole-5-one to Mo(CO)_6_ may facilitate both the deprotection and the reductive cleavage of the O–N bond.^16c^

Then, under the optimized conditions as reported in Table 3, the scope of the reaction was briefly analyzed with other *N*-Boc-protected isoxazole-5-ones **4**, bearing different substituents on both the aromatic rings (Table 4). In all the cases, we obtained methylketones **5** in high yields and with a very high dr, unchanged with respect to starting materials **4**. The relative configuration was determined to be (R*,R*) by comparison of experimental and calculated ^1^H NMR spectra determined on **5a**, **5b**, and **5c**. This was achieved generating conformers for each diastereomeric species using confab^27^ run with an energy window of 5 kcal mol^–1^. These conformers have been then reoptimized using Gaussian 16^28^ at the B3LYP-gCP-D3/6-31G* scheme (see the Supporting Information for further details).^29,30^ For analogy, this relative configuration can be extended to all the other ketones **5** and subsequently to Michael adducts **4**.

**Table 4:**
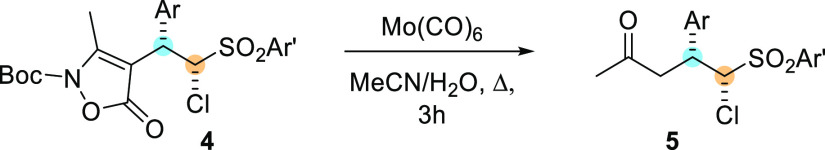
Scope of the Mo(CO)_6_-Mediated Reaction

entry	**5**	Ar	Ar′	*t* (h)	yield^a^	dr
1	**5a**	C_6_H_5_	C_6_H_5_	3	87	>95:5
2	**5b**	4-Cl-C_6_H_4_	4-CN-C_6_H_4_	6	88	>95:5
3	**5c**	C_6_H_5_	4-CN-C_6_H_4_	3	85	>99:1
4	**5d**	C_6_H_5_	4-NO_2_-C_6_H_4_	4	92	>99:1
5	**5e**	4-Cl-C_6_H_4_	4-NO_2_-C_6_H_4_	4	93	>99:1

a Isolated yield.

**Scheme 4:**
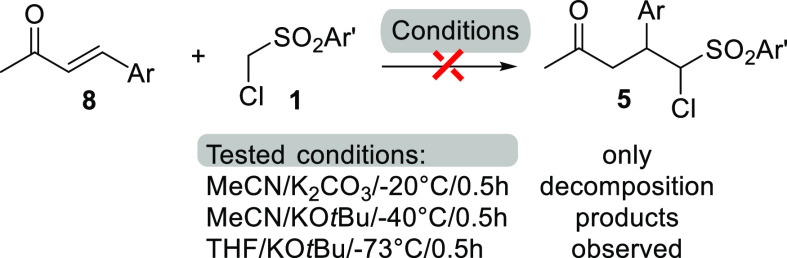
Control Experiments for the Reaction of ((Chloromethyl)sulfonyl)benzenes with (*E*)-4-Arylbut-3-en-2-one

As discussed in the Introduction section, 1,4-conjugate addition of ((chloromethyl)sulfonyl)benzenes has been exploited in cyclopropanation reactions,^1^ while the isolation of the Michael adducts is quite rare.^2^ Nevertheless, ketones **5** may be obtained without involving isoxazol-5-one chemistry by direct 1,4-conjugate addition of ((chloromethyl)sulfonyl)benzenes **1** to the α,β-unsaturated ketones **8** (Scheme 4). Several reaction conditions were tested as reported by Makosza et al. in the conjugated addition of ((chloromethyl)sulfonyl)benzene to nitrochalcone^2^ or in accordance to the conditions described in Table 1. In all the cases, we obtained complex mixtures of unknown products, further demonstrating the utility of the approach herein described.

## Conclusions

1,4-Conjugate additions of ((chloromethyl)sulfonyl)benzene to arylideneisoxazol-5-ones were investigated. In order to overcome the drawbacks related of the scarce stability of the obtained Michael adducts, an effective *N*-trapping by a sequential one-pot addition of electrophiles was developed. This strategy allowed the isolation of a wide range of new, stable isoxazole-5-ones in good yields and with high diastereomeric ratios. Then, the obtained products were subjected to the reductive cleavage of the O–N bond in the presence of the Mo(CO)_6_/H_2_O system. This further investigation led to development of an effective cascade reaction, leading to a new class of methylketones β-substituted with an unprecedented chloromethinephenylsulfonyl end group.

## Experimental Section

### General Methods

Unless otherwise noted, all chemicals, reagents, and solvents for the performed reactions are commercially available and were used without further purification. In particular, ((chloromethyl)sulfonyl)benzene is commercially available; all the other ((chloromethyl)sulfonyl)benzenes were prepared according to ref (1a), while arylideneisoxazol-5-ones were prepared according to ref (18) and (E)-4-arylbut-3-en-2-ones **8** according to ref (31). All the reactions were monitored by TLC on precoated silica gel plates (0.25 mm) and visualized by fluorescence quenching at 254 nm. Flash chromatography was carried out using silica gel 60 (70–230 mesh, Merck, Darmstadt, Germany). Yields are given for isolated products showing one spot on a TLC plate, and no impurities were detectable in the NMR spectrum. The NMR spectra were recorded on Bruker DRX 600, 400, and 300 MHz spectrometers (600 MHz, ^1^H, 125 MHz, ^13^C; 400 MHz, ^1^H, 100.6 MHz, ^13^C; 300 MHz, ^1^H, 75.5 MHz, ^13^C). The internal reference was set to the residual solvent signals (δ_H_ 7.26 ppm, δ_C_ 77.16 ppm for CDCl_3_). The ^13^C NMR spectra were recorded under broad-band proton decoupling. The following abbreviations are used to indicate the multiplicity in NMR spectra: s-singlet, d-doublet, t-triplet, q-quartet, dd-doublet of doublets, m-multiplet, and brs-broad signal. Coupling constants (*J*) are quoted in Hertz. High-resolution mass spectroscopy (HRMS) spectra were acquired using a Bruker SolariX XR Fourier transform ion cyclotron resonance mass spectrometer (Bruker Daltonik GmbH, Bremen, Germany) equipped with a 7T refrigerated actively shielded superconducting magnet. At LMU München, HRMS spectra were recorded on a Finnigan MAT 90, a Finnigan MAT 95, a Thermo Finnigan LTQ FT Ultra Fourier Transform ion cyclotron resonance, or a Q Exactive GC Orbitrap GC/MS. For ionization of the samples, either electron-impact ionization (EI) or electrospray ionization (ESI) was applied. Selected IR spectra (**4i**, **4m**, **4o**, and **5c**) were recorded in KBr on a Bruker Vertex 70 spectrometer.

### General Procedure for the Synthesis of Compounds **4a–4q**

4-Alkylideneisoxazol-5-ones **2** (0.107 mmol, 1.0 equiv) were added to a solution of ((chloromethyl)sulfonyl)benzenes **1** (0.128 mmol, 1.2 equiv) and potassium *tert*-butoxide (0.107 mmol, 12 mg, 1.0 equiv) in anhydrous CH_3_CN (0.21 M, 0.50 mL) at −20 °C. The reaction mixture was monitored by TLC until complete disappearance of starting materials; after that, the reaction mixture was treated with the electrophilic trapping reagents (E–X = Boc_2_O or Ac_2_O or CH_3_I, 0.214 mmol, 2 equiv) and warmed to room temperature. The reaction mixture was allowed to stir until the disappearance of the starting materials on TLC (hexane/ethyl acetate = 80:20). The solution was evaporated, affording the crude product as a white solid, which was purified by column chromatography (hexane/ethyl acetate 80:20) to provide **4a–4q** (63–86%). The reaction with substrate **2a** was scaled to 0.535 mmol (100 mg), leading to the product in 68% yield (0.364 mmol, 174 mg).

#### *tert*-Butyl 4-(2-chloro-2-((4-cyanophenyl)sulfonyl)-1-phenylethyl)-3-methyl-5-oxoisoxazole-2(5*H*)-carboxylate (**4a**)

White solid (70%, 36 mg). A mixture of diastereoisomers, dr 94:6.^1^H NMR (400 MHz, CDCl_3_): δ 7.96 (d, *J* = 7.8 Hz, 2H), 7.69 (t, *J* = 7.8 Hz, 1H), 7.58 (t, *J* = 7.8 Hz, 2H, major + minor), 7.46 (d, *J* = 6.6 Hz, 2H), 7.38–7.28 (m, 3H), 6.15 (d, *J* = 11.1 Hz, 1H, major), 6.09 (d, *J* = 10.7 Hz, 1H, minor), 4.33 (d, *J* = 11.1 Hz, 1H, major + minor), 2.60 (s, 1H, major), 2.54 (s, 1H, minor), 1.56 (s, 9H, major + minor). ^13^C{^1^H} NMR (101 MHz, CDCl_3_): δ 166.5, 154.2, 145.4, 138.8, 136.7, 134.7, 129.7, 129.2, 128.3, 105.0, 86.7, 72.3, 42.8, 28.1, 13.1. MALDI-HRMSl: found *m*/*z*, 516.0692 calcd for C_23_H_24_ClNO_6_SK^+^ (M + K)^+^, 516.0644.

#### *tert*-Butyl 4-(2-chloro-2-((4-cyanophenyl)sulfonyl)-1-phenylethyl)-3-methyl-5-oxoisoxazole-2(5*H*)-carboxylate (**4b**)

White solid (83%, 45 mg). Single diastereomer. mp 205–207 °C (chloroform/hexane). ^1^H NMR (400 MHz, CDCl_3_): δ 8.09 (d, *J* = 8.4 Hz, 2H), 7.87 (d, *J* = 8.4 Hz, 2H), 7.46 (d, *J* = 6.4 Hz, 2H), 7.32 (q, *J* = 8.5, 7.5 Hz, 3H), 6.20 (d, *J* = 11.1 Hz, 1H), 4.33 (d, *J* = 11.1 Hz, 1H), 2.60 (s, 3H), 1.56 (s, 9H). ^13^C{^1^H} NMR (101 MHz, CDCl_3_): δ 166.5, 154.4, 145.3, 141.0, 138.3, 132.9, 130.3, 129.3, 128.5, 128.2, 118.4, 117.1, 104.5, 87.0, 72.2, 42.7, 28.1, 13.1. ESI-HRMS: found, *m*/*z*, 501.0902 calcd for C_24_H_23_ClN_2_O_6_S^–^ (M)^−^, 501.0893.

#### *tert*-Butyl 4-(2-chloro-2-((4-nitrophenyl)sulfonyl)-1-phenylethyl)-3-methyl-5-oxoisoxazole-2(5*H*)-carboxylate (**4c**)

White solid (84%, 47 mg). Single diastereomer. mp 186–188 °C (chloroform/hexane). ^1^H NMR (400 MHz, CDCl_3_): δ 8.42 (d, *J* = 8.9 Hz, 2H), 8.18 (d, *J* = 8.9 Hz, 2H), 7.46 (d, *J* = 7.9 Hz, 2H), 7.33 (q, *J* = 8.8, 7.8 Hz, 3H), 6.23 (d, *J* = 11.1 Hz, 1H), 4.34 (d, *J* = 11.1 Hz, 1H), 2.61 (s, 3H), 1.56 (s, 9H). ^13^C{^1^H} NMR (101 MHz, CDCl_3_): δ 166. 6, 154.5, 151.3, 145.3, 142.4, 138.2, 131.1, 129.4, 128.5, 128.2, 124.4, 104.4, 87.0, 72.2, 42.6, 28.1, 13.1. ESI-HRMS: found, 540.1201 calcd for C_23_H_27_ClN_3_O_8_S^+^ (M + NH_4_)^+^, 540.1207.

#### *tert*-Butyl 4-(2-chloro-1-(4-methoxyphenyl)-2-(phenylsulfonyl)ethyl)-3-methyl-5-oxoisoxazole-2(5*H*)-carboxylate (**4d**)

White solid (63%, 34 mg). Single diastereomer. mp 235–237 °C (chloroform/hexane). ^1^H NMR (400 MHz, CDCl_3_): δ 7.95 (d, *J* = 7.1 Hz, 2H), 7.71–7.67 (m, 1H), 7.58 (d, *J* = 7.7 Hz, 2H), 7.39 (d, *J* = 8.7 Hz, 2H), 6.84 (d, *J* = 8.7 Hz, 2H), 6.09 (d, *J* = 11.1 Hz, 1H), 4.28 (d, *J* = 11.1 Hz, 1H), 3.77 (s, 3H), 2.59 (s, 3H), 1.56 (s, 9H). ^13^C{^1^H} NMR (101 MHz, CDCl_3_): δ 166.6, 159.4, 154.0, 145.4, 136.7, 134.6, 130.9, 129.6, 129.4, 129.2, 114.5, 105.3, 86.7, 72.6, 55.4, 42.0, 28.1, 13.1. ESI-HRMS: found, 530.1010 *m*/*z* calcd for C_24_H_26_ClNaNO_7_^+^ (M + Na)^+^, 530.1016.

#### *tert*-Butyl 4-(2-chloro-2-((4-cyanophenyl)sulfonyl)-1-(4-methoxyphenyl)ethyl)-3-methyl-5-oxoisoxazole-2(5*H*)-carboxylate (**4e**)

White solid (85%, 49 mg). A mixture of diastereomers, dr 92:8. ^1^H NMR (300 MHz, CDCl_3_): δ 8.14 (d, *J* = 7.4 Hz, 2H, minor), 8.08 (d, *J* = 8.0 Hz, 2H, major), 7.86 (d, *J* = 8.2 Hz, 2H, major), 7.77 (d, *J* = 7.9 Hz, 2H, minor), 7.38 (d, *J* = 8.9 Hz, 2H), 6.85 (d, *J* = 8.4 Hz, 2H), 6.14 (d, *J* = 11.0 Hz, 1H), 4.28 (d, *J* = 11.0 Hz, 1H), 3.78 (s, 3H), 2.67 (s, 3H, minor), 2.59 (s, 3H, major), 1.57 (s, 9H, major + minor). ^13^C{^1^H} NMR (75 MHz, CDCl_3_): δ 166.6, 159.6, 154.3, 145.4, 141.0, 132.9, 132.6, 130.3, 130.3, 129.7, 129.6, 129.4, 118.3, 117.1, 114.6, 104.8, 87.0, 74.4 (minor), 72.5 (major), 55.4, 44.5 (minor), 41.8 (major), 28.1, 13.1. ESI-HRMS: found, 531.1009 *m*/*z* calcd for C_25_H_24_ClN_2_O_7_S^–^ (M)^−^, 531.0998.

#### *tert*-Butyl 4-(2-chloro-1-(4-methoxyphenyl)-2-((4-nitrophenyl)sulfonyl)ethyl)-3-methyl-5-oxoisoxazole-2(5*H*)-carboxylate (**4f**)

White solid (80%, 47 mg). A mixture of diastereomers, dr 84:16. ^1^H NMR (400 MHz, CDCl_3_): δ 8.40 (d, *J* = 8.8 Hz, 2H, major), 8.21 (d, *J* = 8.8 Hz, 2H, minor), 8.16 (d, *J* = 8.8 Hz, 2H, major), 7.81 (d, *J* = 8.8 Hz, 2H, minor), 7.38 (d, *J* = 8.7 Hz, 2H, major), 7.32 (d, *J* = 8.7 Hz, 2H, minor), 6.85 (d, *J* = 8.7 Hz, 2H, major), 6.67 (d, *J* = 8.7 Hz, 2H, minor), 6.17 (d, *J* = 11.0 Hz, 1H, major + minor), 4.29 (d, *J* = 11.1 Hz, 1H, major + minor), 3.77 (s, 3H, major), 3.72 (s, 3H, minor), 2.59 (s, 3H, major), 2.53 (s, 3H, minor), 1.56 (s, 9H, major + minor). ^13^C{^1^H} NMR (75 MHz, CDCl_3_): δ 166.7, 159.7, 154.3, 151.3, 145.4, 142.5, 131.1, 130.3, 129.4, 124.3, 124.0, 114.6, 104.7, 87.0, 74.5 (minor), 72.6 (major), 55.4, 44.6 (minor), 41.9 (major), 28.1, 13.1. ESI-HRMS: found, 575.0854 *m*/*z* calcd for C_24_H_25_ClNaN_2_O_9_S^+^ (M + Na)^+^, 575.0854.

#### *tert*-Butyl 4-(2-chloro-1-(4-chlorophenyl)-2-(phenylsulfonyl)ethyl)-3-methyl-5-oxoisoxazole-2(5*H*)-carboxylate (**4g**)

White solid (84%, 46 mg). A mixture of diastereomers, dr 82:18. ^1^H NMR (300 MHz, CDCl_3_): δ 7.95 (d, *J* = 7.8 Hz, 2H), 7.68 (d, *J* = 7.1 Hz, 2H), 7.57 (t, *J* = 7.6 Hz, 2H), 7.41 (d, *J* = 8.4 Hz, 2H, major + minor), 7.29 (d, *J* = 8.4 Hz, 2H, major), 6.07 (d, *J* = 11.1 Hz, 2H), 4.32 (d, *J* = 11.1 Hz, 1H), 2.59 (s, 1H, major), 2.53 (s, 3H, minor), 1.56 (s, 9H). ^13^C{1H} NMR (63 MHz, CDCl_3_): δ 166.4, 154.4, 145.4, 137.2, 136.6, 134.7, 134.2, 129.7, 129.6, 129.5, 129.3, 129.2, 129.1, 104.5, 86.8, 73.9 (minor), 72.1 (major), 44.3 (minor), 42.3 (major), 28.1, 13.9(minor), 13.1 (major). ESI-HRMS: found, 529.0960 *m*/*z* calcd for C_23_H_27_Cl_2_N_2_O_6_S^+^ (M + NH_4_)^+^, 529.0961.

#### *tert*-Butyl 4-(2-chloro-1-(4-chlorophenyl)-2-((4-cyanophenyl)sulfonyl)ethyl)-3-methyl-5-oxoisoxazole-2(5*H*)-carboxylate (**4h**)

White solid (86%, 49 mg). Single diastereomers. mp 235–237 °C (ethyl acetate/hexane). ^1^H NMR (300 MHz, CDCl_3_): δ 8.07 (d, *J* = 8.0 Hz, 2H), 7.87 (d, *J* = 8.3 Hz, 2H), 7.41 (d, *J* = 8.3 Hz, 2H), 7.30 (d, *J* = 8.4 Hz, 2H), 6.13 (d, *J* = 10.9 Hz, 1H), 4.32 (d, *J* = 11.1 Hz, 1H), 2.59 (s, 3H), 1.57 (s, 9H). ^13^C{^1^H} NMR (101 MHz, CDCl_3_): δ 166.5, 154.7, 145.3, 140.8, 136.7, 134.5, 133.0, 130.3, 129.6, 129.5, 118.5, 117.1, 104.0, 87.2, 72.0, 42.1, 28.1, 13.1. ESI-HRMS: found, 535.0514 *m*/*z* calcd for C_24_H_21_Cl_2_N_2_O_6_S^–^ (M)^−^, 535.0503.

#### *tert*-Butyl 4-(2-chloro-1-(4-chlorophenyl)-2-((4-nitrophenyl)sulfonyl)ethyl)-3-methyl-5oxoisoxazole-2(5*H*)-carboxylate (**4i**)

White solid (83%, 50 mg). Single diastereomer. mp 221–223 °C (chloroform/hexane). IR (KBr) ν: 1767; 1732; 1608; 1535; 1338; 1144; 734 cm^–1^. ^1^H NMR (400 MHz, CDCl_3_): δ 8.43 (d, *J* = 8.8 Hz, 2H), 8.17 (d, *J* = 8.8 Hz, 2H), 7.43 (d, *J* = 8.4 Hz, 2H), 7.32 (d, *J* = 8.4 Hz, 2H), 6.18 (d, *J* = 11.1 Hz, 1H), 4.35 (d, *J* = 11.1 Hz, 1H), 2.62 (s, 3H), 1.54 (s, 9H). ^13^C{^1^H} NMR (101 MHz, CDCl_3_): δ 166.5, 154.7, 151.4, 145.3, 142.3, 136.6, 134.5, 131.1, 129.6, 129.5, 124.9, 103.9, 87.2, 72.0, 42.1, 28.1, 13.1. ESI-HRMS: found, 555.0413 *m*/*z* calcd for C_23_H_21_Cl_2_N_2_O_8_S^–^ (M)^−^, 555.0401.

#### 2-Acetyl-4-(2-chloro-1-phenyl-2-(phenylsulfonyl)ethyl)-3-methylisoxazol-5(2*H*)-one (**4j**)

White solid (75%, 34 mg). A mixture of diastereomers, dr 84:16. ^1^H NMR (400 MHz, CDCl_3_): δ 7.97 (d, *J* = 1.0 Hz, 1H), 7.95 (d, *J* = 1.0 Hz, 1H), 7.72 (t, *J* = 1.2 Hz, 1H, minor), 7.71–7.68 (m, 1H), 7.61–7.56 (m, 3H), 7.47–7.42 (m, 3H), 7.36–7.29 (m, 3H), 7.24–7.23 (m, 1H, minor), 6.09 (d, *J* = 11.2 Hz, 1H, major), 6.04 (d, *J* = 10.7 Hz, 1H, minor), 4.40 (d, *J* = 10.7 Hz, 1H, minor), 4.34 (d, *J* = 11.2 Hz, 1H, major), 2.68 (s, 3H, major), 2.62 (s, 3H, minor), 2.40 (s, 3H, major), 2.39 (s, 3H, minor). ^13^C{^1^H} NMR (101 MHz, CDCl_3_) 165.9, 165.1, 154.6 (minor), 153.5 (major), 138.4, 136.5, 134.7, 129.7 (major), 129.6 (minor), 129.3, 129.2 (major), 129.1 (minor), 128.4 (minor), 128.2 (major), 107 (minor), 106.3 (major), 74.0 (minor), 72 (major), 44.5 (minor), 42.6 (major), 22.9 (minor), 22.6 (major), 14.2 (minor), 13.4 (major). ESI-HRMS: found, 442.0487 *m*/*z* calcd for C_20_H_18_ClNaNO_5_S^+^ (M + Na)^+^, 442.0486.

#### 4-((2-(2-Acetyl-3-methyl-5-oxo-2,5-dihydroisoxazol-4-yl)-1-chloro-2phenylethyl)sulfonyl) Benzonitrile (**4k**)

White solid (82%, 39 mg). Single diastereomer. mp 220–222 °C (ethyl acetate/hexane).^1^H NMR (250 MHz, CDCl_3_): δ 8.09 (d, J = 8.3 Hz, 2H), 7.88 (d, *J* = 8.3 Hz, 2H), 7.46 (d, *J* = 5.9 Hz, 2H), 7.40–7.30 (m, 3H), 6.14 (d, *J* = 11.1 Hz, 1H), 4.36 (d, *J* = 11.2 Hz, 1H), 2.68 (s, 3H), 2.41 (s, 3H). ^13^C{^1^H} NMR (63 MHz, CDCl_3_): δ 166.0, 165.1, 153.8, 140.8, 137.8, 132.9, 130.3, 129.4, 128.6, 128.2, 118.4, 117.1, 105.8, 77.7, 76.7, 72.0, 42.3, 22.8, 13.3. EI-HRMS: found, 444.0537 *m*/*z* calcd for C_21_H_17_ClN_2_O_5_S (M), 444.0547.

#### 2-Acetyl-4-(2-chloro-2-((4-nitrophenyl)sulfonyl)-1-phenylethyl)-3-methylisoxazol-5(2*H*)-one (**4l**)

White solid (70%, 40 mg). Single diastereomer. mp 226–228 °C (chloroform/hexane). ^1^H NMR (400 MHz, CDCl_3_): δ 8.42 (d, *J* = 8.7 Hz, 2H), 8.18 (d, *J* = 8.7 Hz, 2H), 7.46 (d, *J* = 7.5 Hz, 2H), 7.38–7.30 (m, 3H), 6.16 (d, *J* = 11.1 Hz, 1H), 4.37 (d, *J* = 11.1 Hz, 1H), 2.69 (s, 3H), 2.42 (s, 3H). ^13^C{^1^H} NMR (101 MHz, CDCl_3_) δ 166.0, 165.1, 153.8, 151.5, 142.2, 137.8, 131.2, 129.5, 128.6, 128.2, 124.4, 105.8, 72.1, 42.3, 22.9, 13.4. MALDI-HRMS: found *m*/*z*, 487.0359 calcd for C_20_H_17_ClNaN_2_O_7_S^+^ (M + Na)^+^, 487.0337.

#### 4-((2-(2-Acetyl-3-methyl-5-oxo-2,5-dihydroisoxazol-4-yl)-1-chloro-2-(4-chlorophenyl)ethyl)sulfonyl)benzonitrile (**4m**)

White solid (86%, 44 mg). Single diastereomer. mp 202–204 °C (ethyl acetate/hexane). IR (KBr) ν: 2235; 1762; 1721; 1617; 1305 cm^–1^. ^1^H NMR (300 MHz, CDCl_3_): δ 8.08 (d, *J* = 8.5 Hz, 2H), 7.88 (d, *J* = 8.5 Hz, 2H), 7.41 (d, *J* = 8.6 Hz, 2H), 7.31 (d, *J* = 8.6 Hz, 2H), 6.06 (d, *J* = 11.0 Hz, 1H), 4.34 (d, *J* = 11.1 Hz, 1H), 2.68 (s, 3H), 2.42 (s, 3H). ^13^C{^1^H} NMR (101 MHz, CDCl_3_): δ 165.7, 164.9, 153.8, 140.4, 136.1, 134.4, 132.8, 130.1, 129.4, 118.3, 116.8, 105.1, 71.6, 41.6, 22.6, 13.2. ESI-HRMS: found, 501.0044 *m*/*z* calcd for C_21_H_16_Cl_2_NaN_2_O_5_S^+^ (M + Na)^+^, 501.0055.

#### 2-Acetyl-4-(2-chloro-1-(4-chlorophenyl)-2-((4-nitrophenyl) sulfonyl)ethyl)-3-methylisoxazol-5(2*H*)-one (**4n**)

White solid (72%, 39 mg). A mixture of diastereomers, dr 97:3. ^1^H NMR (400 MHz, CDCl_3_): δ 8.42 (d, *J* = 8.8 Hz, 2H), 8.16 (d, *J* = 8.8 Hz, 2H), 7.41 (d, *J* = 8.4 Hz, 2H), 7.32 (d, *J* = 8.4 Hz, 2H), 6.09 (d, *J* = 11.1 Hz, 1H), 4.36 (d, *J* = 11.1 Hz, 1H), 2.69 (s, 3H), 2.43 (s, 3H). ^13^C{1H} NMR (101 MHz, CDCl_3_): δ 166.0, 165.1, 154.0, 151.5, 142.1, 136.2, 134.7, 131.2, 129.6, 124.4, 105.2, 71.8, 41.8, 22.9, 13.4. ESI-HRMS: found, 498.0051 *m*/*z* calcd for C_20_H_16_Cl_2_N_2_O_7_S (M), 498.0055.

#### 4-(2-Chloro-1-phenyl-2-(phenylsulfonyl)ethyl)-2,3-dimethylisoxazol-5(2*H*)-one (**4o**)

White solid (75% 32 mg). A mixture of diastereoisomers, dr 86:14. IR (KBr) ν: 1715; 1571; 1322; 1143; 752 cm^–1^. ^1^H NMR (300 MHz, CDCl_3_): δ 7.96–7.92 (m, 2H, major + minor), 7.67 (t, *J* = 7.4 Hz, 1H, major), 7.58–7.49 (m, 5H), 7.42–7.37 (m, 1H, minor), 7.34–7.27 (m, 3H), 7.22–7.19 (m, 1H), 6.15 (d, *J* = 11.0 Hz, 1H, major + minor), 4.27 (d, *J* = 11.2 Hz, 1H, major + minor), 3.28 (s, 3H, major + minor), 2.24 (s, 3H, major), 2.17 (s, 3H, minor). ^13^C{^1^H} NMR (75 MHz, CDCl_3_): δ 169.8, 160.5, 139.6, 137.1, 134.4, 129.5, 129.1, 129.0, 128.4, 128.0, 100.9, 72.8, 43.4, 37.6, 10.7. ESI-HRMS: found, 392.0718 *m*/*z* calcd for C_19_H_18_ClNO_4_S^+^ (M + H)^+^, 392.0718.

#### 4-((1-Chloro-2-(2,3-dimethyl-5-oxo-2,5-dihydroisoxazol-4-yl)-2-phenylethyl)sulfonyl) Benzonitrile (**4p**)

White solid (80%, 36 mg). A mixture of diastereomers, dr 71:29. ^1^H NMR (300 MHz, CDCl_3_): δ 8.07 (d, *J* = 8.4 Hz, 2H, major), 7.85 (d, *J* = 8.4 Hz, 2H, major), 7.75 (d, *J* = 8.5 Hz, 2H, minor), 7.66 (d, *J* = 8.5 Hz, 2H, minor), 7.50 (d, *J* = 7.2 Hz, 2H, major), 7.45 (d, *J* = 7.7 Hz, 2H, minor), 7.35–7.28 (m, 4H, major + minor), 7.20 (d, *J* = 6.8 Hz, 1H), 6.21 (d, *J* = 11.0 Hz, 2H, major + minor), 4.26 (d, *J* = 10.9 Hz, 2H, major + minor), 3.32 (s, 3H, major + minor), 2.24 (s, 1H, major), 2.17 (s, 1H, minor). ^13^C{^1^H} NMR (101 MHz, CDCl_3_): δ 169.6, 160.2, 141.1, 139.0, 133.2, 132.7, 132.5, 130.0, 129.5, 129.0, 128.3, 128.2, 128.0, 118.0, 117.0, 100.0, 72.5, 43.0, 37.4, 10.6. ESI-HRMS: found, 417.0670 *m*/*z* calcd for C_20_H_18_ClN_2_O_4_S^+^ (M + H)^+^, 417.0670.

#### 4-((1-Chloro-2-(4-chlorophenyl)-2-(2,3-dimethyl-5-oxo-2,5-dihydroisoxazol-4-yl)ethyl) sulfonyl)benzonitrile (**4q**)

White solid (76%, 37 mg). A mixture of diastereomers, dr 93:7. ^1^H NMR (300 MHz, CDCl_3_): δ 8.01 (d, *J* = 8.1 Hz, 2H), 7.81 (d, *J* = 8.1 Hz, 2H), 7.41 (d, *J* = 7.7 Hz, 2H), 7.23 (d, *J* = 4.4 Hz, 2H), 6.09 (d, *J* = 10.9 Hz, 1H), 4.20 (d, *J* = 11.1 Hz, 1H), 3.30 (s, 3H, major), 3.05 (s, 3H, minor), 2.20 (s, 9H, major), 2.13 (s, 9H, minor). ^13^C{^1^H} NMR (75 MHz, CDCl_3_): δ 169.7, 160.2, 141.1, 137.6, 134.2, 132.9, 130.2, 129.8, 129.3, 128.4, 118.3, 117.1, 99.5, 72.4, 42.6, 37.5, 10.7. ESI-HRMS: found, 451.0270 *m*/*z* calcd for C_20_H_17_Cl_2_N_2_O_4_S^+^ (M + H)^+^, 451.0286.

### Mo(CO)_6_-Mediated Reductive Cascade Reactions

Molybdenum hexacarbonyl (0.067 mmol, 18 mg, 1.0 equiv) was added to a solution of **4** (0.067 mmol, 1.0 equiv) in a H_2_O/MeCN mixture (0.2 + 1.3 mL) at 85 °C in an oil bath. The reaction mixture was monitored by TLC until complete disappearance of starting materials. The reaction mixture was allowed to cool down to room temperature, diluted with CHCl_3_, and filtered over Celite. The solvent was evaporated, affording the crude product as a yellow solid, which was purified by column chromatography (hexane: ethyl acetate from 95:5 to 80:20) to provide products **5a–5e** (84–93%). The reaction of **4h** was scaled to 0.201 mmol (108 mg, 1 equiv), affording **5b** in 80% yield (0.161 mmol, 64 mg).

#### 5-Chloro-4-phenyl-5-(phenylsulfonyl)pentan-2-one (**5a**)

Colorless oil (87%, 20 mg). Single diastereomer. ^1^H NMR (400 MHz, CDCl_3_): δ 7.85 (d, *J* = 7.2 Hz, 2H), 7.64 (t, *J* = 7.5 Hz, 1H), 7.51 (t, *J* = 7.8 Hz, 2H), 7.39–7.28 (m, 5H), 5.29 (d, *J* = 5.3 Hz, 1H), 4.58–4.26 (m, 1H), 3.28 (dd, *J* = 18.2, 7.6 Hz, 1H), 3.06 (dd, *J* = 18.2, 6.0 Hz, 1H), 2.13 (s, 3H). ^13^C{^1^H} NMR (101 MHz, CD_2_Cl_2_): δ 206.0, 138.2, 137.5, 134.9, 130.2, 130.1, 129.6, 128.7, 128.4, 77.5, 47.2, 41.1, 30.7. ESI-HRMS: found, 375.0223 calcd for C_17_H_17_ClKO_3_S^+^ (M + K)^+^, 375.0219.

#### 4-((1-Chloro-2-(4-chlorophenyl)-4-oxopentyl)sulfonyl)benzonitrile (**5b**)

Colorless oil (88%, 24 mg). Single distereomer. ^1^H NMR (300 MHz, CDCl_3_): δ 7.94 (d, *J* = 8.2 Hz, 2H), 7.80 (d, *J* = 8.2 Hz, 2H), 7.34–7.28 (m, 4H), 5.34 (d, *J* = 4.7 Hz, 1H), 4.37–4.33 (m, 1H), 3.20 (dd, *J* = 18.5, 8.1 Hz, 1H), 2.99 (dd, *J* = 18.5, 5.4 Hz, 1H), 2.15 (s, 3H). ^13^C{^1^H} NMR (151 MHz, CD_2_Cl_2_): δ 205.8, 141.2, 136.1, 134.5, 133.3, 131.6, 130.8, 129.0, 118.6, 117.6, 77.3, 46.8, 40.1, 30.7. ESI-HRMS: found, 418.0053 *m*/*z* calcd for C_18_H_15_Cl_2_NNaO_3_S^+^ (M + Na)^+^, 418.0047.

#### 4-((1-Chloro-4-oxo-2-phenylpentyl)sulfonyl)benzonitrile (**5c**)

Colorless oil (85%, 21 mg). Single diastereomer. IR (KBr) ν: 2236; 1719; 1335; 1156; 647 cm^–1^. ^1^H NMR (300 MHz, CDCl_3_): δ 7.84 (d, *J* = 8.4 Hz, 2H), 7.70 (d, *J* = 8.5 Hz, 2H), 7.50–7.00 (m, 5H), 5.37 (d, *J* = 4.9 Hz, 1H), 4.33–4.27 (m, 1H), 3.20 (dd, *J* = 18.4, 8.2 Hz, 1H), δ 2.97 (dd, *J* = 18.4, 5.4 Hz, 1H), 2.11 (s, 3H). ^13^C{^1^H} NMR (75 MHz, CD_2_Cl_2_): δ 205.7, 140.8, 137.2, 132.8, 130.4, 129.8, 128.4, 128.3, 118.0, 117.3, 77.2, 46.6, 40.5, 30.3. ESI-HRMS: found, 360.0474 *m*/*z* calcd for C_18_H_15_ClNO_3_S^–^ (M)^−^, 360.0467.

#### 5-Chloro-5-((4-nitrophenyl)sulfonyl)-4-phenylpentan-2-one (**5d**)

Colorless oil (92%, 24 mg). Single diastereomer. ^1^H NMR (400 MHz, CDCl_3_): δ 8.28 (d, *J* = 8.9 Hz, 2H), 7.95 (d, *J* = 8.8 Hz, 2H), 7.53–7.18 (m, 5H), 5.44 (d, *J* = 4.8 Hz, 1H), 4.49–4.25 (m, 1H), 3.24 (dd, *J* = 18.4, 8.2 Hz, 1H), 3.01 (dd, *J* = 18.4, 5.4 Hz, 1H), 2.15 (s, 3H). ^13^C{^1^H} NMR (75 MHz, CD_2_Cl_2_): δ 206.0, 151.5, 142.7, 137.5, 131.7, 130.2, 128.9, 128.7, 124.5, 77.7, 47.0, 40.9, 30.7. ESI-HRMS: found, 380.0372 *m*/*z* calcd for C_17_H_15_ClNO_5_S^–^ (M)^−^, 380.0365.

#### 5-Chloro-4-(4-chlorophenyl)-5-((4-nitrophenyl)sulfonyl)pentan-2-one (**5e**)

Colorless oil (93%, 26 mg). Single diastereomer. ^1^H NMR (300 MHz, CDCl_3_): δ 8.35 (d, *J* = 8.7 Hz, 2H), 8.03 (d, *J* = 8.8 Hz, 2H), 7.66–7.26 (m, 4H), 5.37 (d, *J* = 4.7 Hz, 1H), 4.40–4.34 (m, 1H), 3.21 (dd, *J* = 18.5, 8.2 Hz, 1H), 3.00 (dd, *J* = 18.5, 5.3 Hz, 1H), 2.16 (s, 3H). ^13^C{^1^H} NMR (101 MHz, CD_2_Cl_2_): δ 205.8, 151.7, 142.7, 136.1, 134.6, 131.7, 131.6, 129.0, 124.6, 77.3, 46.8, 40.2, 30.7. ESI-HRMS: found 413.9980 *m*/*z* calcd for C_17_H_14_Cl_2_NO_5_S^–^ (M)^−^, 413.9975.

## Supporting Information Available

Copies of ^1^H and ^13^C{1H} NMR spectra for the series of compunds **4** and **5**; calculated 1H NMR spectra of **5a**, **5b**, and **5c**, FTIR spectra of selected compounds (PDF)
