# Supplementary material for: Access to β-Alkylated γ-Functionalized Ketones via Conjugate Additions to Arylideneisoxazol-5-ones and Mo(CO)6-Mediated Reductive Cascade Reactions
Source: ACS Omega. 2022 Mar 4;7(10):8808–18. doi: 10.1021/acsomega.1c07081 (PMC8928520; doi:10.1021/acsomega.1c07081)
Supplement: Supplementary file 1 — ao1c07081_si_001.pdf [file ao1c07081_si_001.pdf]

SUPPORTING INFORMATION

Access to β-Alkylated γ-Functionalized Ketones via Conjugate Additions to Arylideneisoxazol-5-ones and $Mo(CO)_6$-Mediated Reductive Cascade Reactions

Antonio Macchia,[a] Francesco F. Summa,[a] Guglielmo Monaco,[a] Andreas Eitzinger,[b] Armin R. Ofial[b] Antonia Di Mola[a] and Antonio Massa*,[a]

[a]Dipartimento di Chimica e Biologia "A. Zambelli", Università degli studi di Salerno, Via Giovanni Paolo II, 84084-Fisciano (SA), Italy.

[b]Department Chemie, Ludwig-Maximilians-Universität Munchen, 81377 Munchen, Germany

Table of Contents

^{1}HNMR calculations for **5a**, **5b** and **5c** S2

Cartesian coordinates........ S5

References S17

Copies of ^{1}H-NMR and ^{13}C-NMR spectra S18

Copies of IR spectra of selected compounds........S40

Calculations of ^{1}HNMR spectra of 5a, 5b and 5c.

For each diastereomeric species, conformers have been first generated using confab[1] run with an energy window of 5 kcal mol^{-1}. Those conformers have been then re-optimized using Gaussian 16[2] at the B3LYP-gCP-D3/6-31G* scheme,[3,4] which corrects for missing London dispersion and basis set superposition error. The version with the BJ-damping[5] has been used. Relative energies of conformers in gas phase remaining after removal of duplicates are given in Table S1. Minimum energy conformers are sketched in Figures S1.

Table S1. Relative energies of optimized conformers computed at the B3LYP-gCP-D3/6-31G* level for each of the 6 diastereomers studied. Absolute energies of the minimum energy conformer at the same level are also given.

Conformer	1	2	3	4	5	6	7	8	9	E_{min} (hartree)
**5a**-(*R*,*R*)	0.00	3.71	2.44	0.27	1.80	3.51	3.32	4.44		-1742.13829951
**5a**-(*R*,*S*)	2.82	0.00	3.64	2.07	3.37	1.15	1.76	3.88	1.12	-1742.13672629
**5b**-(*R*,*R*)	0.00	2.03	2.03							-2293.98212096
**5b**-(*R*,*S*)	2.79	1.89	0.31	0.00	0.86					-2293.92885717
**5c**-(*R*,*R*)	0.00	1.83	0.47	3.86	5.51	3.87	3.74			-1834.38690012
**5c**-(*R*,*S*)	1.78	0.42	0.98	0.00	1.73					-1834.38354936

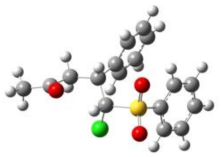	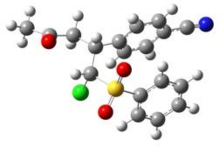	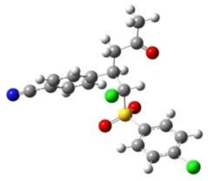
**5a**-*(R,R)*	**5c**-*(R,R)*	**5b**-*(R,R)*
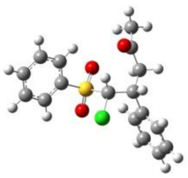	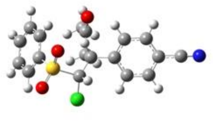	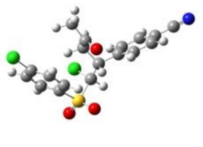
**5a**-*(R,S)*	**5c**-*(R,S)*	**5b**-*(R,S)*

Figure S1. Geometries of the minimum energy conformers obtained at the B3LYP-gCP-D3/6-31G* level.

Nuclear shielding constants have been computed for all conformers at the B3LYP/6-31+G** level using GIAO and the SMD protocol[6] to model the effect of chloroform. Conversion from nuclear shielding constants to chemical shifts has been afforded with the recommended values for intercept and slope for the method used, published on the CHESHIRE website (slope=-1.0472, intercept=31.6874 ppm).[7,8] Conformer populations to be used in the thermal average at 298 K of nuclear shieldings have been computed considering either

I) E(B3LYP-gCP-D3/6-31G*),
II) G(B3LYP-gCP-D3/6-31G*) or
III) G_{solv}(B3LYP-gCP-D3/6-31G*).

The solvent correction to the energy in calculation III) has been estimated from the difference of two single point calculations at the level used for the chemical shift calculations, i.e. B3LYP/6-31+G** level, one including and the other devoid of the scrf=(solvent=chloroform,smd) keyword in Gaussian. The agreement between calculated and experimental chemical shifts has been evaluated with the sample standard deviation *s* between observed and calculated values. Results are gathered in Table S2.

Table S2. Experimental and computed proton chemical shifts for the two stereoisomers of **5a**, **5b** and **5c**. Chemical shifts have been computed at the B3LYP/6-31+G**//B3LYP-gCP-D3/6-31G* level, weighting the conformers populations according to the three schemes described above. All values in ppm.

	δ_{exp}	δ_{I}(R,R)	δ_{I}(R,S)	δ_{II}(R,R)	δ_{II}(R,S)	δ_{III}(R,R)	δ_{III}(R,S)
**5a**							
CH_3	2.13	2.23	2.32	2.20	2.20	2.14	2.32
CH_2(H2)	3.06	3.03	2.96	2.99	3.19	2.79	2.96
CH_2(H1)	3.28	3.05	3.99	3.18	3.85	3.31	3.99
CH-Ph	4.32	3.81	3.75	3.99	4.09	4.26	3.75
CH-Cl	5.29	5.92	5.74	5.78	5.15	5.59	5.74
s		0.42	0.50	0.30	0.31	0.21	0.50
**5b**							
CH_3	2.15	2.17	1.89	2.18	2.01	2.17	2.07
CH_2(H2)	2.99	2.75	2.32	2.75	3.20	2.79	3.53
CH_2(H1)	3.2	3.27	2.66	3.28	3.15	3.22	3.27
CH-Ph	4.35	4.42	4.53	4.45	4.59	4.46	4.64
CH-Cl	5.34	5.63	4.73	5.65	4.53	5.58	4.46
s		0.19	0.40	0.20	0.42	0.16	0.51
**5c**							

CH_3	2.11	2.25	1.82	2.24	2.00	2.16	2.05
CH_2(H2)	2.97	2.94	2.26	3.03	3.31	2.85	3.56
CH_2(H1)	3.2	3.06	2.61	3.12	3.17	3.22	3.25
CH-Ph	4.3	3.91	4.50	4.04	4.62	4.36	4.67
CH-Cl	5.37	5.89	4.69	5.80	4.50	5.52	4.47
s		0.34	0.43	0.26	0.47	0.10	0.54

5a-*(R,R)*

6	-3.979122	0.330464	-0.057834
8	-4.057317	-0.474090	0.852988
6	-2.685438	1.068169	-0.374036
1	-2.513702	1.051278	-1.456318
1	-2.841755	2.125929	-0.120201
6	-5.164950	0.657569	-0.941741
1	-5.277754	1.739958	-1.072283
1	-4.999839	0.228815	-1.938666
1	-6.075414	0.231971	-0.516022
6	-1.448779	0.565557	0.391613
1	-1.705082	0.564574	1.455613
6	-1.182751	-0.921125	0.106739
1	-2.015320	-1.505510	0.500401
6	-0.279205	1.512588	0.195192
6	0.196988	2.236519	1.294201
6	0.312078	1.725493	-1.057142
6	1.245153	3.145771	1.152783
1	-0.245525	2.066605	2.271582
6	1.368238	2.623793	-1.198095
1	-0.038852	1.175218	-1.922666
6	1.837716	3.338374	-0.095132
1	1.601597	3.696549	2.018779
1	1.826394	2.763881	-2.172931
1	2.658521	4.040968	-0.208483
17	-1.075679	-1.349380	-1.646071
16	0.244009	-1.644516	1.047168
6	1.762815	-1.131436	0.253153
6	2.514335	-0.113377	0.836534

6	2.186568	-1.790914	-0.902604
6	3.701594	0.280506	0.224029
1	2.163107	0.359648	1.743739
6	3.372548	-1.381956	-1.508538
1	1.603456	-2.610129	-1.304800
6	4.123800	-0.344901	-0.950141
1	4.291312	1.079418	0.662113
1	3.714616	-1.879094	-2.411210
1	5.047548	-0.030746	-1.427726
8	0.122965	-3.099427	0.887699
8	0.171798	-1.008256	2.373526

5a-*(S,S)*

6	0.272840	3.047605	0.341916
8	-0.448029	2.878973	1.312857
6	1.426238	2.114348	0.009198
1	1.464599	1.984369	-1.073771
1	2.355722	2.634042	0.283647
6	0.067720	4.201030	-0.612540
1	1.018182	4.674444	-0.883410
1	-0.372760	3.797183	-1.532150
1	-0.611505	4.935746	-0.175899
6	1.425905	0.754129	0.738911
1	1.567067	0.968200	1.804459
6	0.048810	0.052809	0.734244
1	-0.650614	0.691605	1.270410
6	2.591016	-0.105746	0.283864
6	2.768714	-0.459086	-1.059137
6	3.532116	-0.538529	1.223387
6	3.859557	-1.234589	-1.447489
1	2.047955	-0.149601	-1.807598
6	4.627266	-1.310940	0.837129
1	3.400801	-0.272726	2.269275
6	4.793270	-1.661779	-0.502143
1	3.976943	-1.507763	-2.492190
1	5.346527	-1.638823	1.582380
1	5.643146	-2.265423	-0.807962
17	0.101670	-1.517371	1.604082
16	-0.773431	-0.196191	-0.906884
6	-2.449739	-0.568447	-0.404280
6	-3.249835	0.468969	0.078734

6	-2.911228	-1.879335	-0.499544
6	-4.550496	0.174565	0.481779
1	-2.859469	1.480150	0.136189
6	-4.217935	-2.157147	-0.099520
1	-2.253392	-2.651591	-0.881835
6	-5.031787	-1.134341	0.391196
1	-5.189034	0.965946	0.862128
1	-4.598563	-3.171615	-0.168372
1	-6.047353	-1.357256	0.705223
8	-0.223308	-1.358122	-1.614630
8	-0.779455	1.133698	-1.551228

5c-*(R,R)*

6	-3.956155	1.468794	-0.031937
8	-4.304435	0.708256	0.852593
6	-2.485412	1.733063	-0.330440
1	-2.321282	1.694373	-1.413201
1	-2.273385	2.770969	-0.038089
6	-4.954491	2.207749	-0.897183
1	-4.693067	3.268075	-0.990223
1	-4.938344	1.782277	-1.908947
1	-5.957817	2.101767	-0.480980
6	-1.506964	0.807721	0.414397
1	-1.757288	0.858894	1.478271
6	-1.757438	-0.671782	0.075585
1	-2.736200	-0.953325	0.466040
6	-0.080084	1.292352	0.246875
6	0.605501	1.781512	1.365100
6	0.570160	1.288536	-0.995308
6	1.916435	2.232980	1.262384
1	0.113871	1.777347	2.332977
6	1.885225	1.720169	-1.109803
1	0.058424	0.916298	-1.874449
6	2.569235	2.190155	0.021922
1	2.445200	2.597507	2.136685
1	2.393987	1.684416	-2.066845
17	-1.815706	-1.042491	-1.691693
16	-0.654389	-1.865463	0.973691
6	0.972809	-1.810302	0.230638
6	2.004265	-1.189942	0.934154
6	1.189861	-2.424070	-1.005107

6	3.274591	-1.142716	0.364296
1	1.801152	-0.751446	1.902473
6	2.462616	-2.359021	-1.568490
1	0.382819	-2.945916	-1.504016
6	3.499035	-1.712838	-0.890064
1	4.085954	-0.653572	0.894087
1	2.647526	-2.822351	-2.532844
1	4.488445	-1.663496	-1.335145
8	-1.225278	-3.194188	0.725194
8	-0.560344	-1.306114	2.333273
6	3.940352	2.588182	-0.084712
7	5.060060	2.891578	-0.169770

5c-*(S,S)*

6	-0.241828	2.402250	-0.213543
8	0.000711	2.580183	0.966453
6	-0.165025	1.024873	-0.859978
1	-1.177927	0.773050	-1.191977
1	0.434028	1.088211	-1.775478
6	-0.686814	3.531022	-1.118413
1	-0.014050	3.624853	-1.979027
1	-1.687790	3.314847	-1.511645
1	-0.706463	4.468982	-0.560874
6	0.402808	-0.039311	0.096317
1	0.112892	0.254021	1.109267
6	-0.184809	-1.447398	-0.099580
1	0.484518	-2.206970	0.310225
6	1.922486	-0.039880	0.075363
6	2.649801	-0.611668	-0.978256
6	2.618620	0.580089	1.120372
6	4.039572	-0.577366	-0.983713
1	2.129134	-1.091004	-1.800524
6	4.008473	0.626056	1.123948
1	2.060413	1.043427	1.926910
6	4.728532	0.043698	0.069939
1	4.597884	-1.026321	-1.798322
1	4.541427	1.107797	1.936709
17	-0.493370	-1.905248	-1.814731
16	-1.717685	-1.712083	0.928445
6	-2.818227	-0.387141	0.451804
6	-3.615215	-0.545781	-0.684075
6	-2.804240	0.798733	1.186971

6	-4.398026	0.527996	-1.105287
1	-3.616501	-1.490550	-1.215209
6	-3.594088	1.863460	0.754772
1	-2.179370	0.882296	2.068155
6	-4.381212	1.730032	-0.391595
1	-5.023959	0.425770	-1.986391
1	-3.590205	2.795012	1.311861
1	-4.992859	2.563128	-0.725654
8	-1.262466	-1.463842	2.305532
8	-2.314260	-2.990026	0.528663
6	6.160359	0.085925	0.065158
7	7.322884	0.121706	0.058698

5b-*(R,R)*

6	-0.836595	3.793256	-0.183705
8	0.138983	3.598844	-0.887410
6	-1.771833	2.665330	0.235054
1	-1.790667	2.626613	1.331195
1	-2.792031	2.939104	-0.064076
6	-1.193277	5.175170	0.322777
1	-2.185465	5.469822	-0.040335
1	-1.240166	5.179694	1.418568
1	-0.449385	5.897629	-0.017137
6	-1.428058	1.293926	-0.367428
1	-1.266952	1.442557	-1.440006
6	-0.055761	0.802260	0.128562
1	0.699708	1.534395	-0.152908
6	-2.550558	0.286828	-0.193103
6	-3.192296	0.100483	1.038124
6	-2.961913	-0.477398	-1.292193
6	-4.216025	-0.829575	1.173514
1	-2.888039	0.676654	1.904416
6	-3.984470	-1.410500	-1.171383
1	-2.455131	-0.356508	-2.243934
6	-4.619394	-1.591316	0.066243
1	-4.706828	-0.972798	2.130193
1	-4.293690	-2.002250	-2.026086
17	0.060399	0.579628	1.909073
16	0.442555	-0.758967	-0.735022
6	2.199534	-0.824267	-0.408132
6	3.064653	-0.065833	-1.197762
6	2.666746	-1.621706	0.635638

6	4.430422	-0.098290	-0.931131
1	2.673912	0.527670	-2.017502
6	4.033192	-1.657492	0.901744
1	1.967079	-2.208474	1.219704
6	4.898125	-0.892336	0.118009
1	5.127125	0.477372	-1.529637
1	4.426303	-2.270302	1.704824
17	6.615507	-0.932570	0.455074
8	0.250002	-0.458185	-2.165189
8	-0.188790	-1.920224	-0.101209
6	-5.679870	-2.544947	0.200331
7	-6.544907	-3.314677	0.310296

5b-*(S,S)*

6	-0.162981	2.165021	0.045637
8	0.065197	2.275442	1.237223
6	0.107616	0.872117	-0.718693
1	-0.868066	0.496853	-1.048364
1	0.655329	1.099662	-1.640436
6	-0.790406	3.286177	-0.756310
1	-0.159791	3.548747	-1.614108
1	-1.759307	2.959705	-1.155315
1	-0.934758	4.161530	-0.120453
6	0.864565	-0.176430	0.124459
1	0.574119	-0.019241	1.168061
6	0.477389	-1.637308	-0.181872
1	1.269475	-2.321779	0.132813
6	2.368162	0.058849	0.062676
6	3.158865	-0.424248	-0.990401
6	2.976068	0.822508	1.069436
6	4.525042	-0.163674	-1.033187
1	2.709710	-1.009703	-1.786005
6	4.339920	1.094359	1.034861
1	2.366588	1.217447	1.875803
6	5.125185	0.599359	-0.018660
1	5.131000	-0.545759	-1.848435
1	4.802199	1.684560	1.819502
17	0.149481	-1.989971	-1.922411
16	-0.950356	-2.223364	0.879829
6	-2.262293	-1.048987	0.554245
6	-3.077286	-1.235229	-0.565351
6	-2.387261	0.073293	1.375026

6	-4.017639	-0.259729	-0.888707
1	-2.970429	-2.128355	-1.170900
6	-3.328265	1.049051	1.053005
1	-1.744387	0.190682	2.239849
6	-4.123789	0.877579	-0.082295
1	-4.660820	-0.373666	-1.754228
1	-3.435472	1.936376	1.666694
17	-5.279439	2.123181	-0.510660
8	-0.474736	-2.013762	2.256478
8	-1.367400	-3.541148	0.390935
6	6.531728	0.873867	-0.061523
7	7.672485	1.098354	-0.098671

References

(1) O'Boyle, N. M.; Vandermeersch, T.; Flynn, C. J.; Maguire, A. R.; Hutchison, G. R. Confab - Systematic Generation of Diverse Low-Energy Conformers. *J. Cheminformatics* **2011**, *3* (1), 8. https://doi.org/10.1186/1758-2946-3-8.

(2) M. J. Frisch, G. W. Trucks, H. B. Schlegel, G. E. Scuseria, M. A. Robb, J. R. Cheeseman, G. Scalmani, V. Barone, G. A. Petersson, H. Nakatsuji, X. Li, M. Caricato, A. V. Marenich, J. Bloino, B. G. Janesko, R. Gomperts, B. Mennucci, H. P. Hratchian, J. V. Ortiz, A. F. Izmaylov, J. L. Sonnenberg, D. Williams-Young, F. Ding, F. Lipparini, F. Egidi, J. Goings, B. Peng, A. Petrone, T. Henderson, D. Ranasinghe, V. G. Zakrzewski, J. Gao, N. Rega, G. Zheng, W. Liang, M. Hada, M. Ehara, K. Toyota, R. Fukuda, J. Hasegawa, M. Ishida, T. Nakajima, Y. Honda, O. Kitao, H. Nakai, T. Vreven, K. Throssell, J. A. Montgomery, Jr., J. E. Peralta, F. Ogliaro, M. J. Bearpark, J. J. Heyd, E. N. Brothers, K. N. Kudin, V. N. Staroverov, T. A. Keith, R. Kobayashi, J. Normand, K. Raghavachari, A. P. Rendell, J. C. Burant, S. S. Iyengar, J. Tomasi, M. Cossi, J. M. Millam, M. Klene, C. Adamo, R. Cammi, J. W. Ochterski, R. L. Martin, K. Morokuma, O. Farkas, J. B. Foresman, and D. J. Fox,. *Gaussian16, Revision C.01*; Gaussian, Inc.: Wallingford CT, 2016.

(3) Grimme, S.; Ehrlich, S.; Goerigk, L. Effect of the Damping Function in Dispersion Corrected Density Functional Theory. *J. Comput. Chem.* **2011**, *32* (7), 1456–1465. https://doi.org/10.1002/jcc.21759.

(4) Kruse, H.; Goerigk, L.; Grimme, S. Why the Standard B3LYP/6-31G* Model Chemistry Should Not Be Used in DFT Calculations of Molecular Thermochemistry: Understanding and Correcting the Problem. *J. Org. Chem.* **2012**, *77* (23), 10824–10834. https://doi.org/10.1021/jo302156p.

(5) Johnson, E. R.; Becke, A. D. A Post-Hartree-Fock Model of Intermolecular Interactions: Inclusion of Higher-Order Corrections. *J. Chem. Phys.* **2006**, *124* (17), 174104. https://doi.org/10.1063/1.2190220.

(6) Marenich, A. V.; Cramer, C. J.; Truhlar, D. G. Universal Solvation Model Based on Solute Electron Density and on a Continuum Model of the Solvent Defined by the Bulk Dielectric Constant and Atomic Surface Tensions. *J. Phys. Chem. B* **2009**, *113* (18), 6378–6396. https://doi.org/10.1021/jp810292n.

(7) CHESHIRE Chemical Shift Repository http://cheshirenmr.info/ (accessed 2021 -09 -24).

(8) Lodewyk, M. W.; Siebert, M. R.; Tantillo, D. J. Computational Prediction of ^{1} H and ^{13} C Chemical Shifts: A Useful Tool for Natural Product, Mechanistic, and Synthetic Organic Chemistry. *Chem. Rev.* **2012**, *112* (3), 1839–1862. https://doi.org/10.1021/cr200106v.

^{1}H-NMR and ^{13}C-NMR SPECTRA

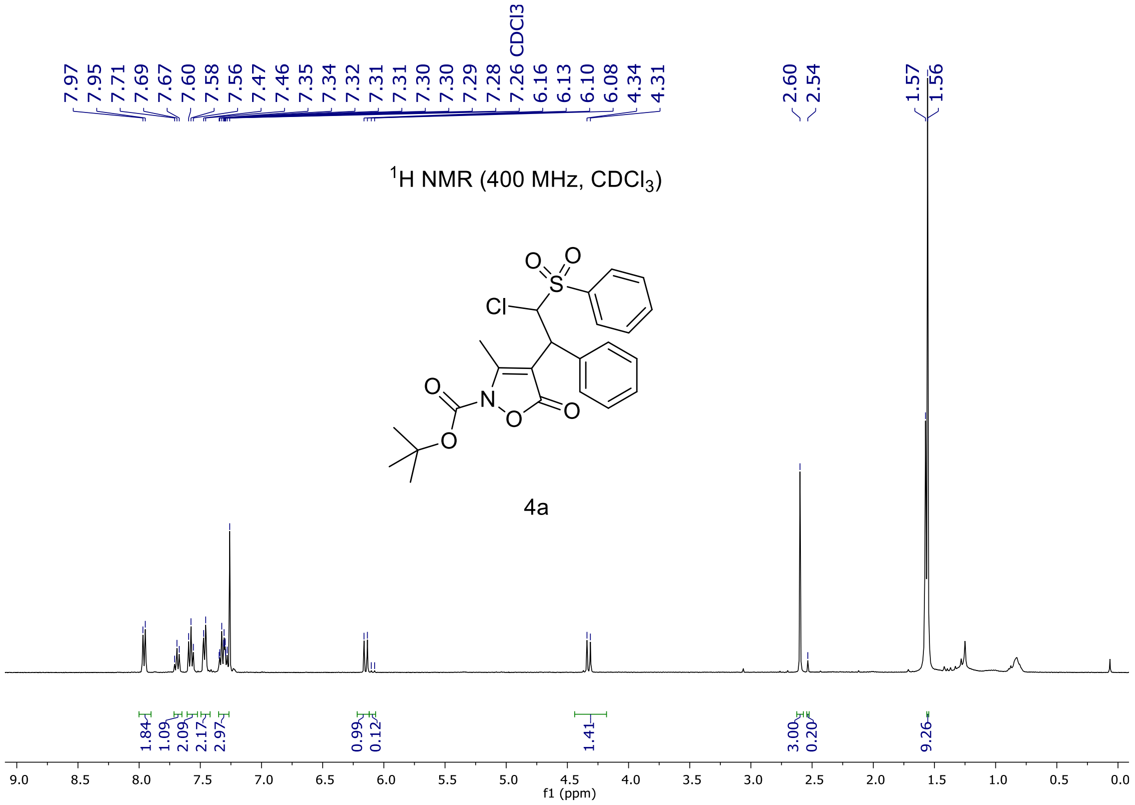

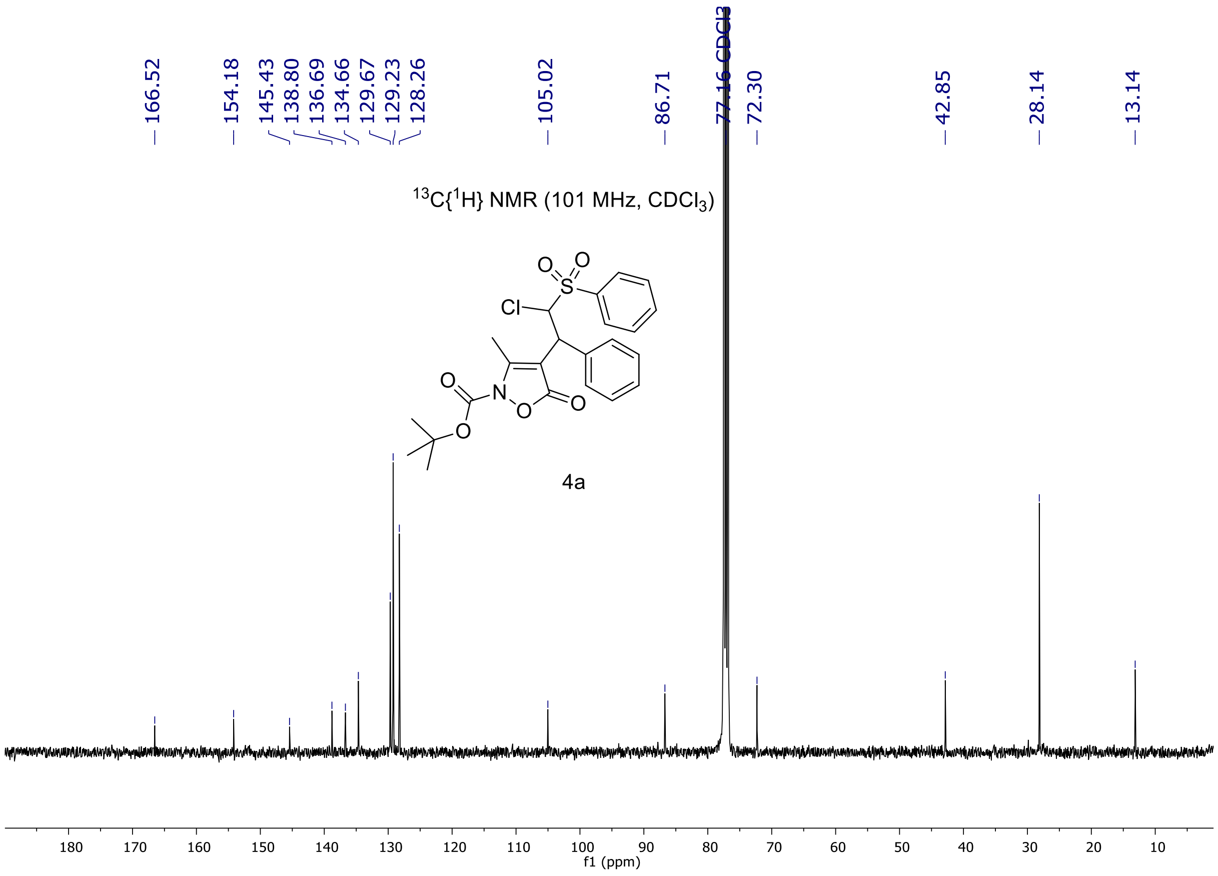

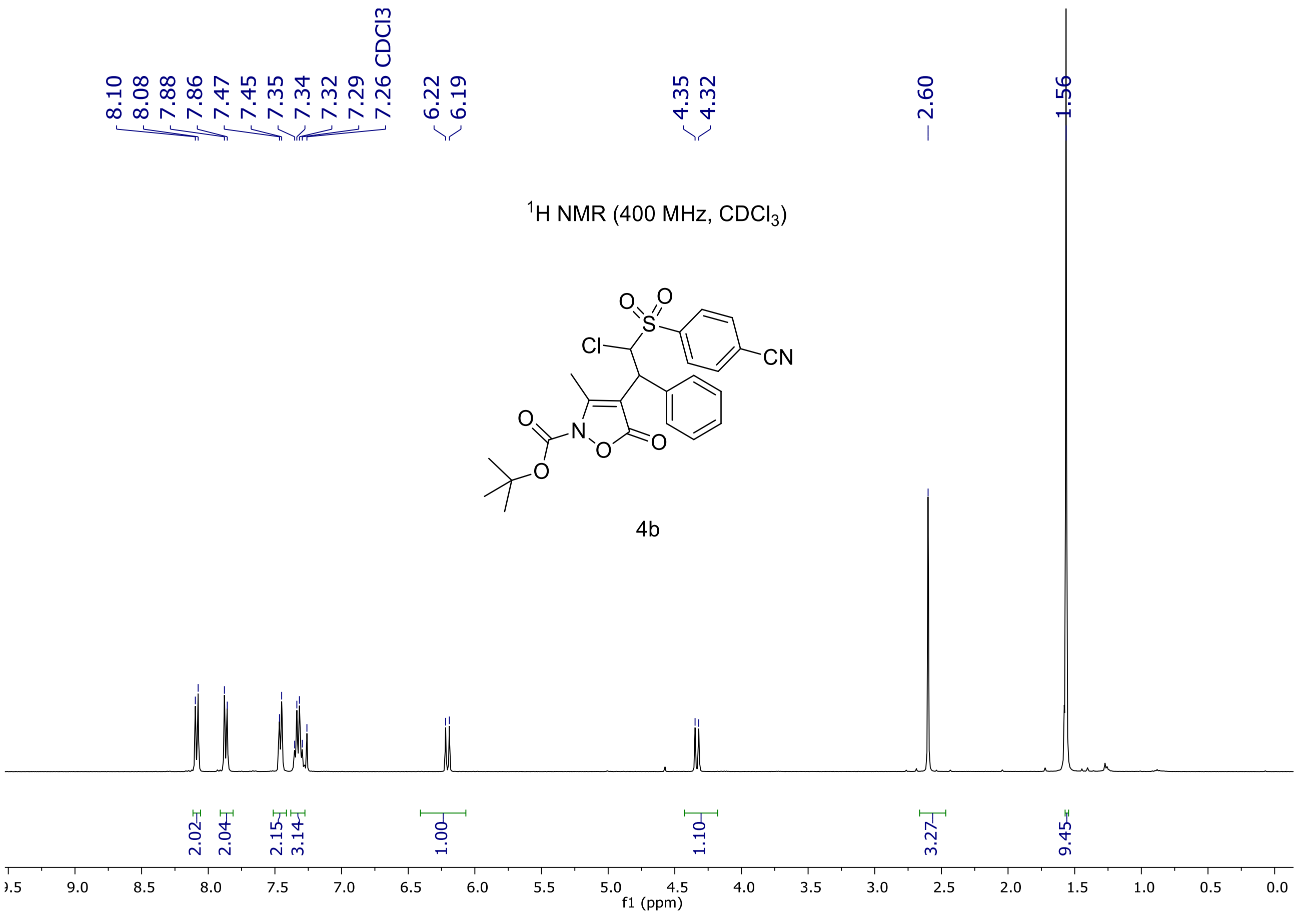

8.10 8.08 7.88 7.86 7.47 7.45 7.35 7.34 7.32 7.29 7.26 CDCl3 6.22 6.19 4.35 4.32 2.60 1.56
^{1}H NMR (400 MHz, CDCl$_3$)
Cl
O
S
CN
N
4b
2.02 2.04 2.15 3.14 1.00 1.10 3.27 9.45
f1 (ppm)

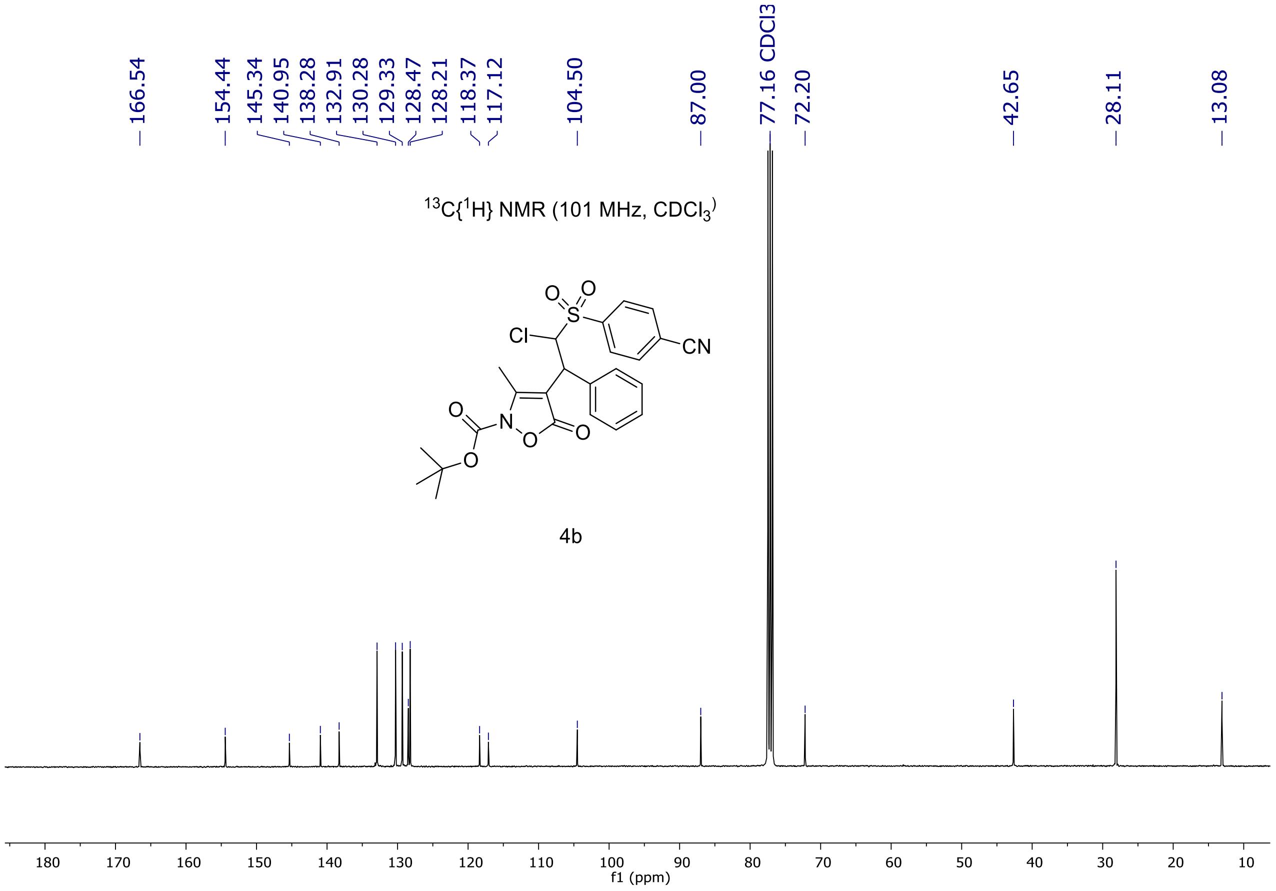

166.54 154.44 145.34 140.95 138.28 132.91 130.28 129.33 128.47 128.21 118.37 117.12 104.50 87.00 77.16 CDCl3 72.20 42.65 28.11 13.08
^{13}C{^{1}H} NMR (101 MHz, CDCl$_3$)
Cl
O
S
CN
N
4b
f1 (ppm)

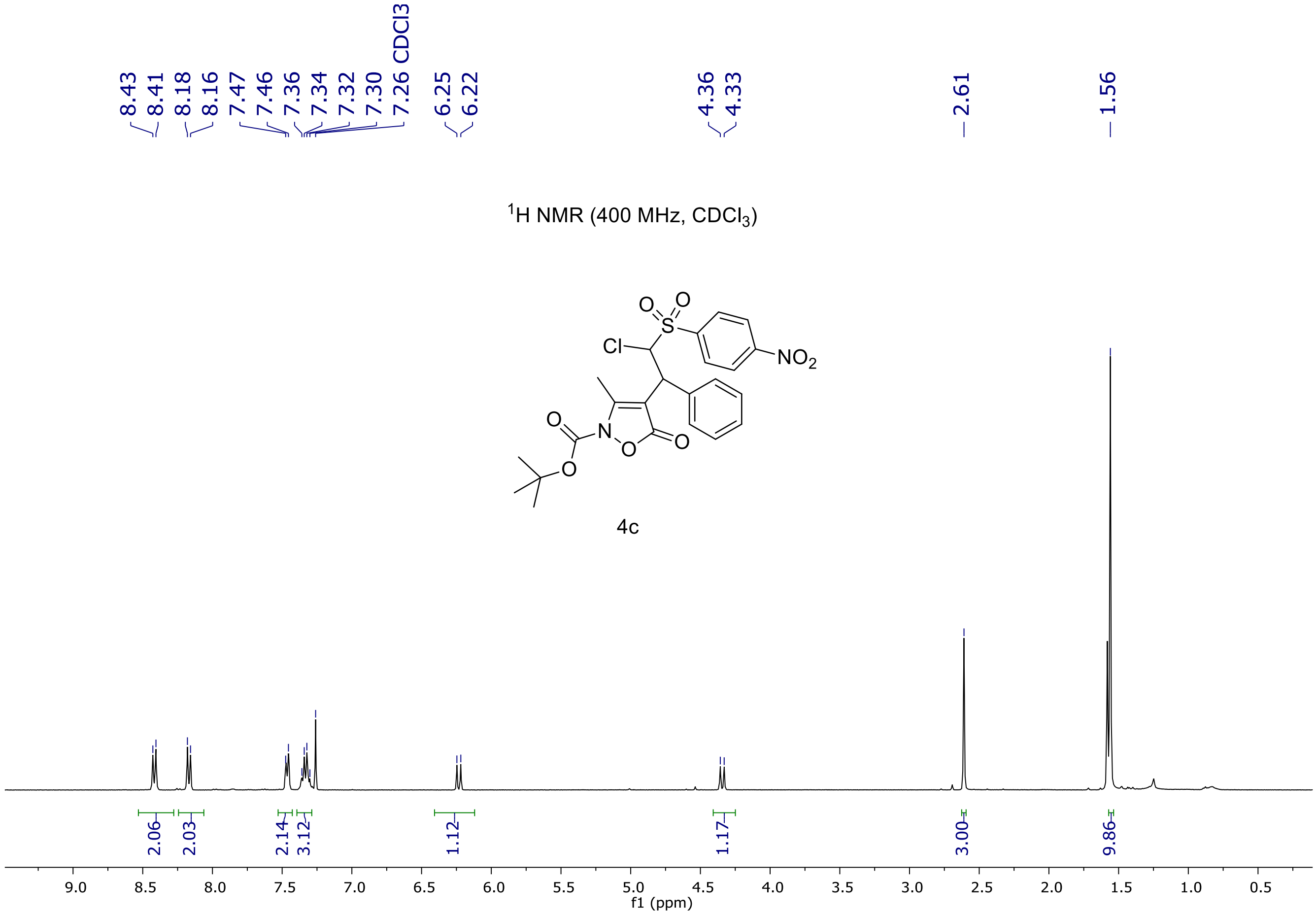
8.43
8.41
8.18
8.16
7.47
7.46
7.36
7.34
7.32
7.30
7.26 CDCl3
6.25
6.22
4.36
4.33
2.61
1.56
Cl
O
S
O
NO2
N
O
O
O
O
4c
2.06
2.03
2.14
3.12
1.12
1.17
3.00
9.86
f1 (ppm)

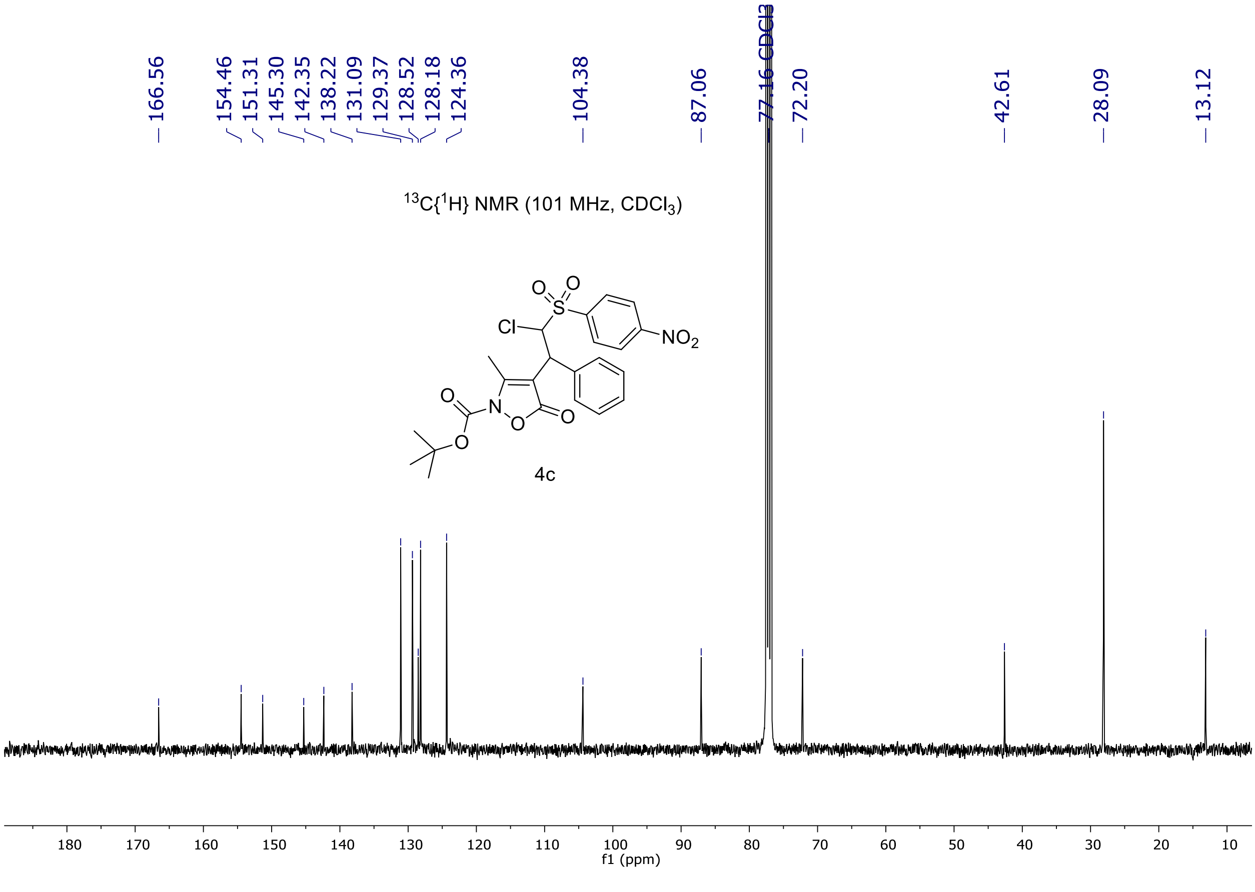
166.56
154.46
151.31
145.30
142.35
138.22
131.09
129.37
128.52
128.18
124.36
104.38
87.06
77.16 CDCl3
72.20
42.61
28.09
13.12
Cl
NO2
4c
f1 (ppm)

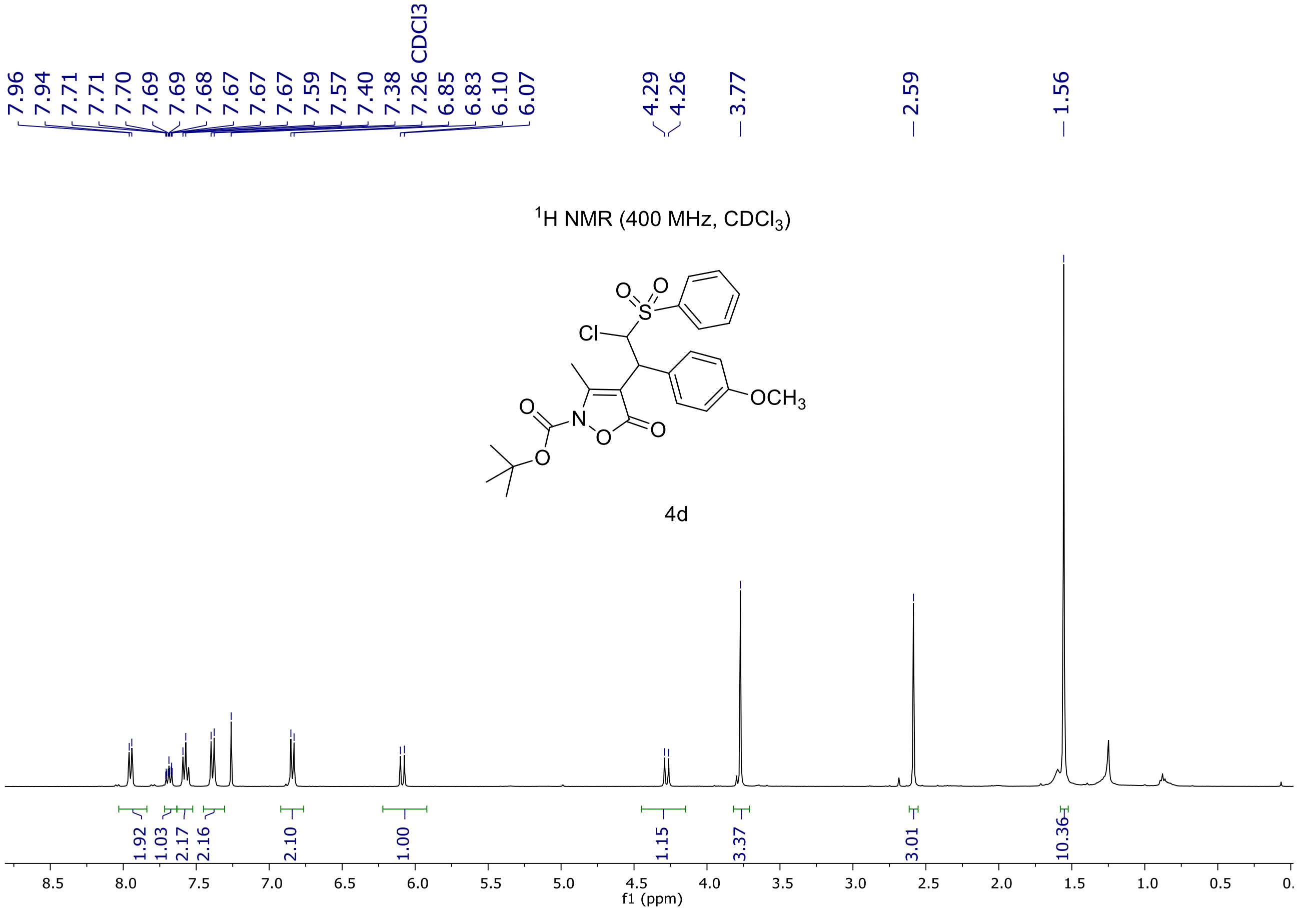
7.96
7.94
7.71
7.71
7.70
7.69
7.69
7.68
7.67
7.67
7.67
7.59
7.57
7.40
7.38
7.26 CDCl3
6.85
6.83
6.10
6.07
4.29
4.26
3.77
2.59
1.56
1H NMR (400 MHz, CDCl3)
Cl
S
O
O
OCH3
N
O
O
O
O
4d
1.92
1.03
2.17
2.16
2.10
1.00
1.15
3.37
3.01
10.36
f1 (ppm)

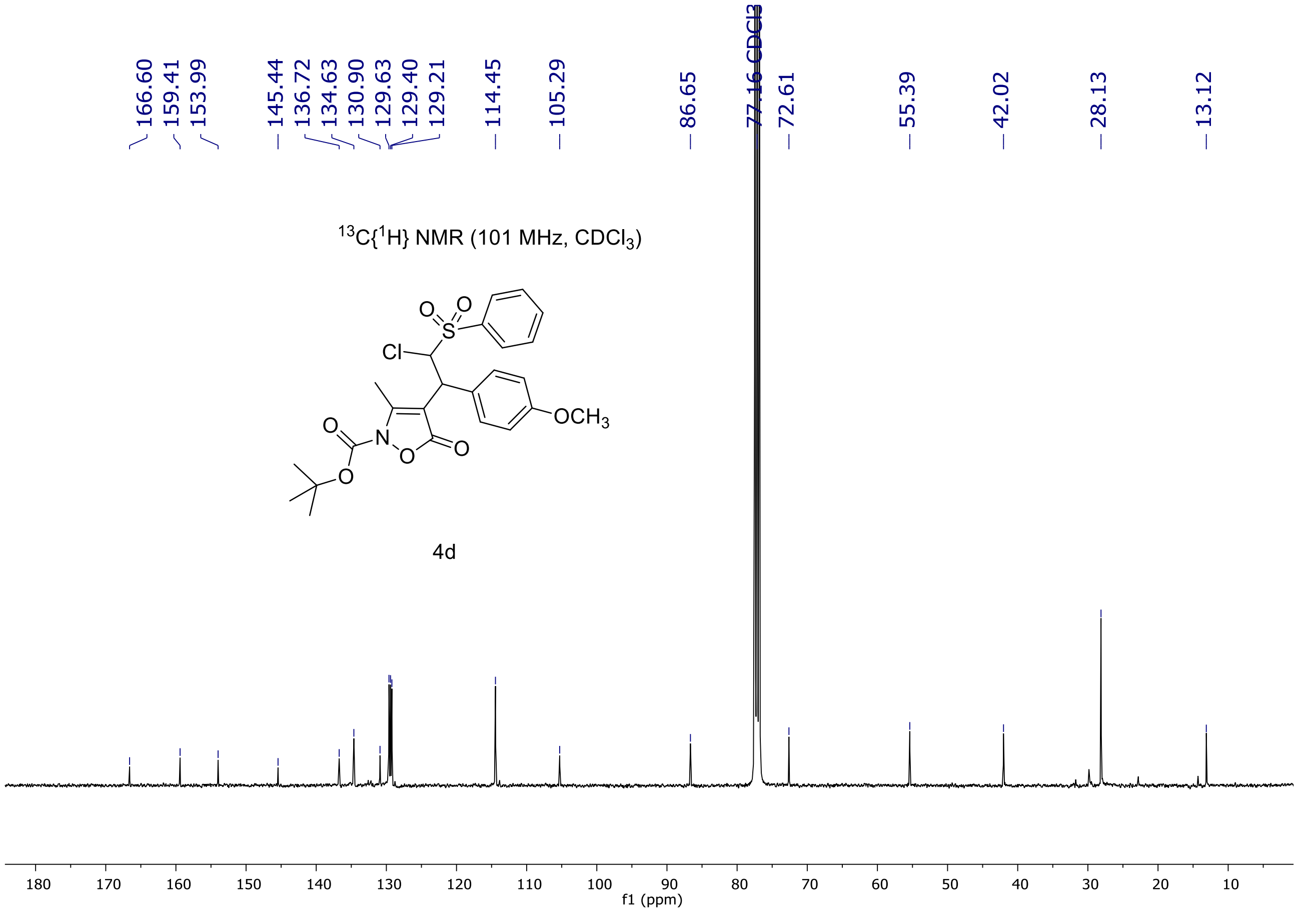
166.60
159.41
153.99
145.44
136.72
134.63
130.90
129.63
129.40
129.21
114.45
105.29
86.65
77.16 CDCl3
72.61
55.39
42.02
28.13
13.12
13C{1H} NMR (101 MHz, CDCl3)
Cl
S
O
O
OCH3
N
O
O
O
O
4d
f1 (ppm)

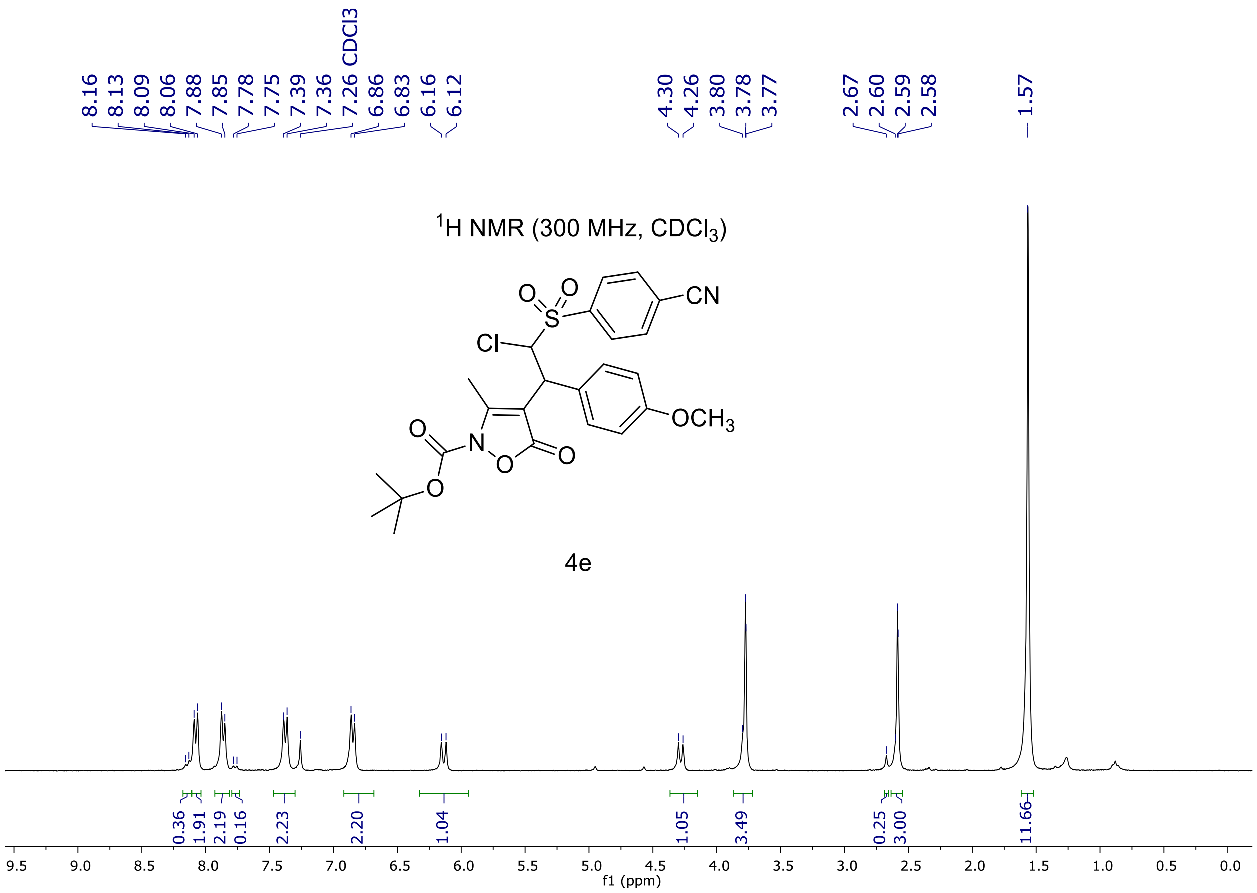
8.16
8.13
8.09
8.06
7.88
7.85
7.78
7.75
7.39
7.36
7.26 CDCl3
6.86
6.83
6.16
6.12
4.30
4.26
3.80
3.78
3.77
2.67
2.60
2.59
2.58
1.57
^{1}H NMR (300 MHz, $CDCl_3$)
CN
Cl
OCH_3
4e
0.36
1.91
2.19
0.16
2.23
2.20
1.04
1.05
3.49
0.25
3.00
11.66
f1 (ppm)

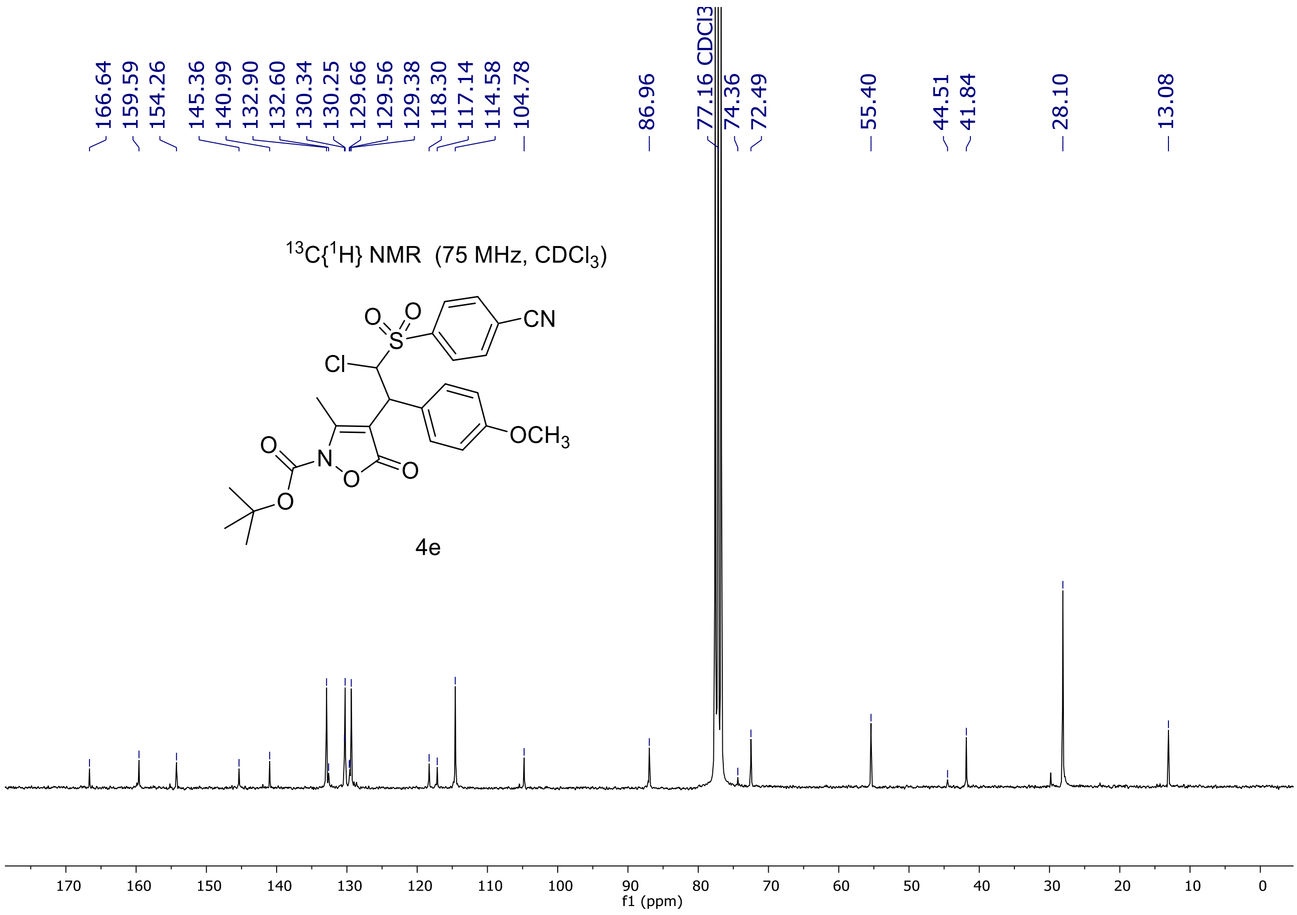
166.64
159.59
154.26
145.36
140.99
132.90
132.60
130.34
130.25
129.66
129.56
129.38
118.30
117.14
114.58
104.78
86.96
77.16 CDCl3
74.36
72.49
55.40
44.51
41.84
28.10
13.08
^{13}C{^{1}H} NMR (75 MHz, $CDCl_3$)
CN
Cl
OCH_3
4e
f1 (ppm)

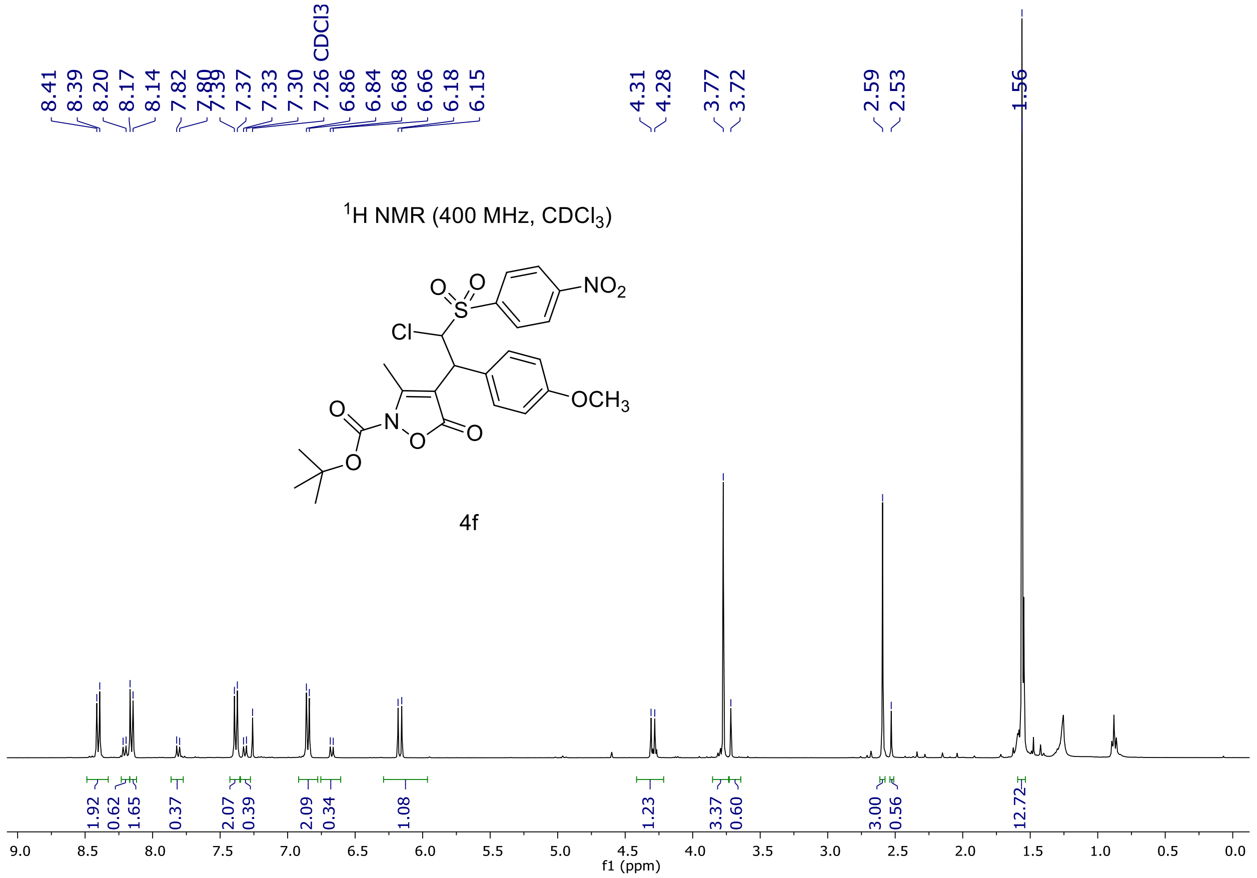
1H NMR (400 MHz, CDCl3)
4f
f1 (ppm)

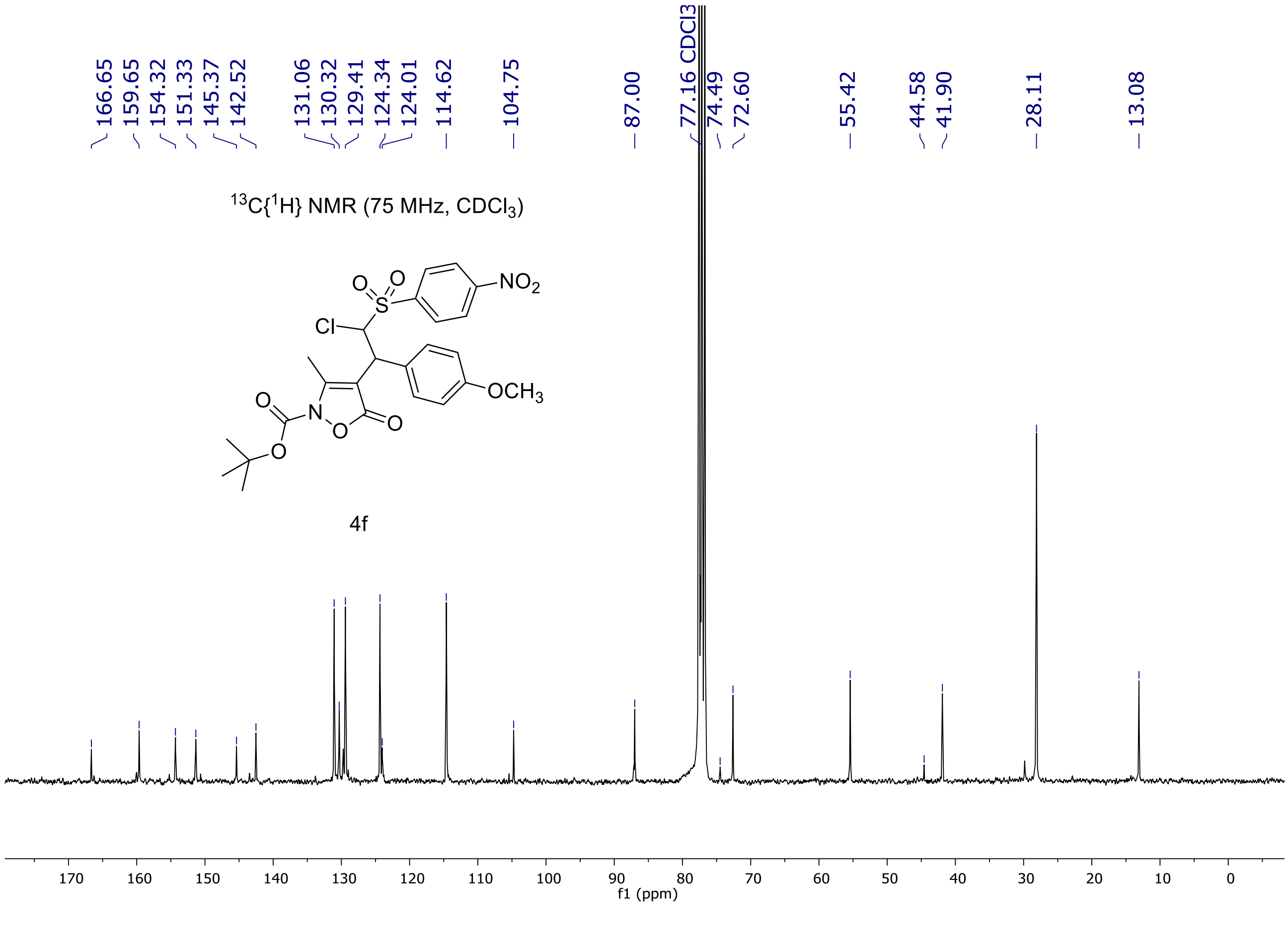
13C{1H} NMR (75 MHz, CDCl3)
4f
f1 (ppm)

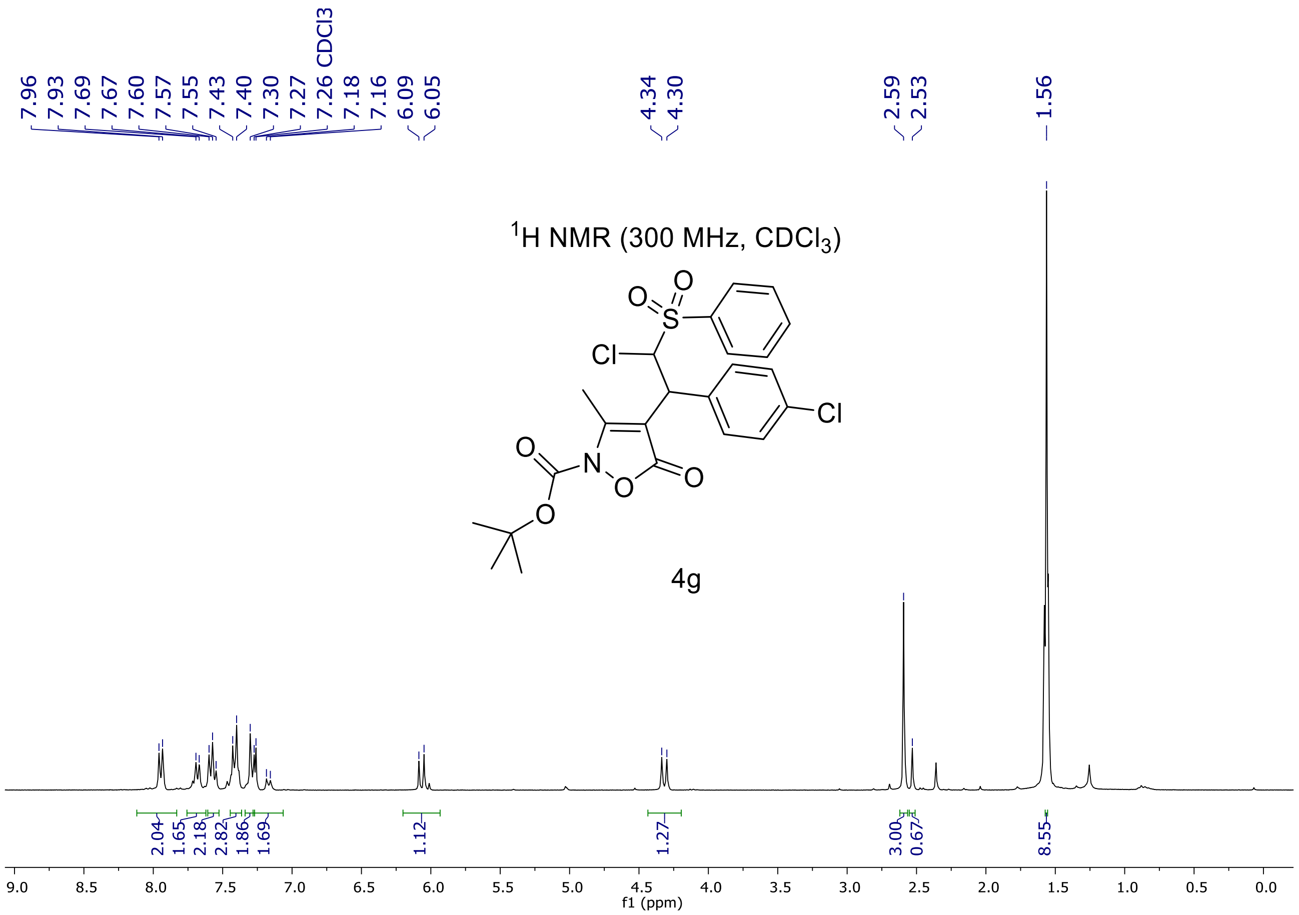

7.96
7.93
7.69
7.67
7.60
7.57
7.55
7.43
7.40
7.30
7.27
7.26 CDCl3
7.18
7.16
6.09
6.05
4.34
4.30
2.59
2.53
1.56
1H NMR (300 MHz, CDCl3)
O
O
S
Cl
Cl
O
N
O
O
O
4g
2.04
1.65
2.18
2.82
1.86
1.69
1.12
1.27
3.00
0.67
8.55
f1 (ppm)

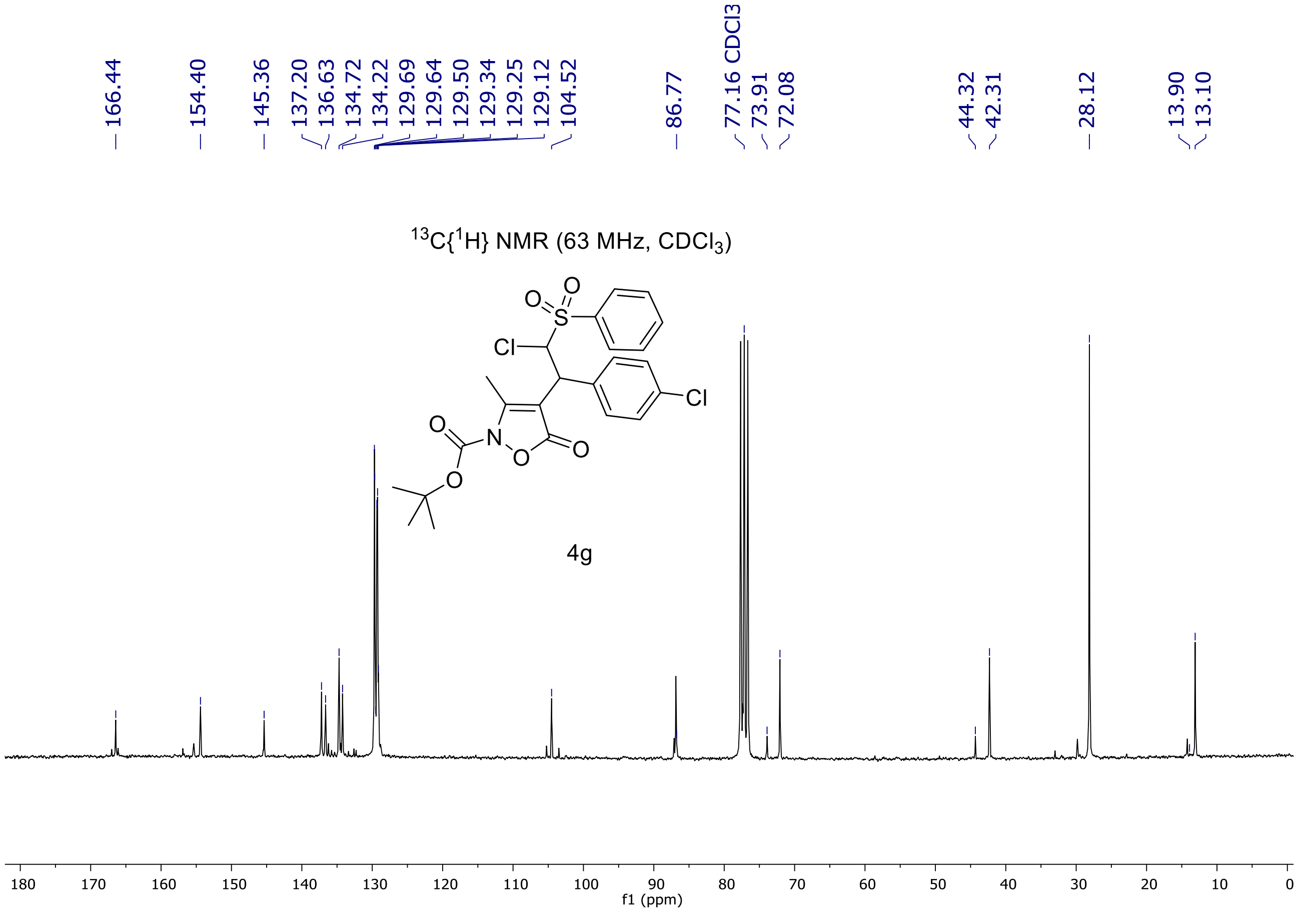

166.44
154.40
145.36
137.20
136.63
134.72
134.22
129.69
129.64
129.50
129.34
129.25
129.12
104.52
86.77
77.16 CDCl3
73.91
72.08
44.32
42.31
28.12
13.90
13.10
13C{1H} NMR (63 MHz, CDCl3)
4g
f1 (ppm)

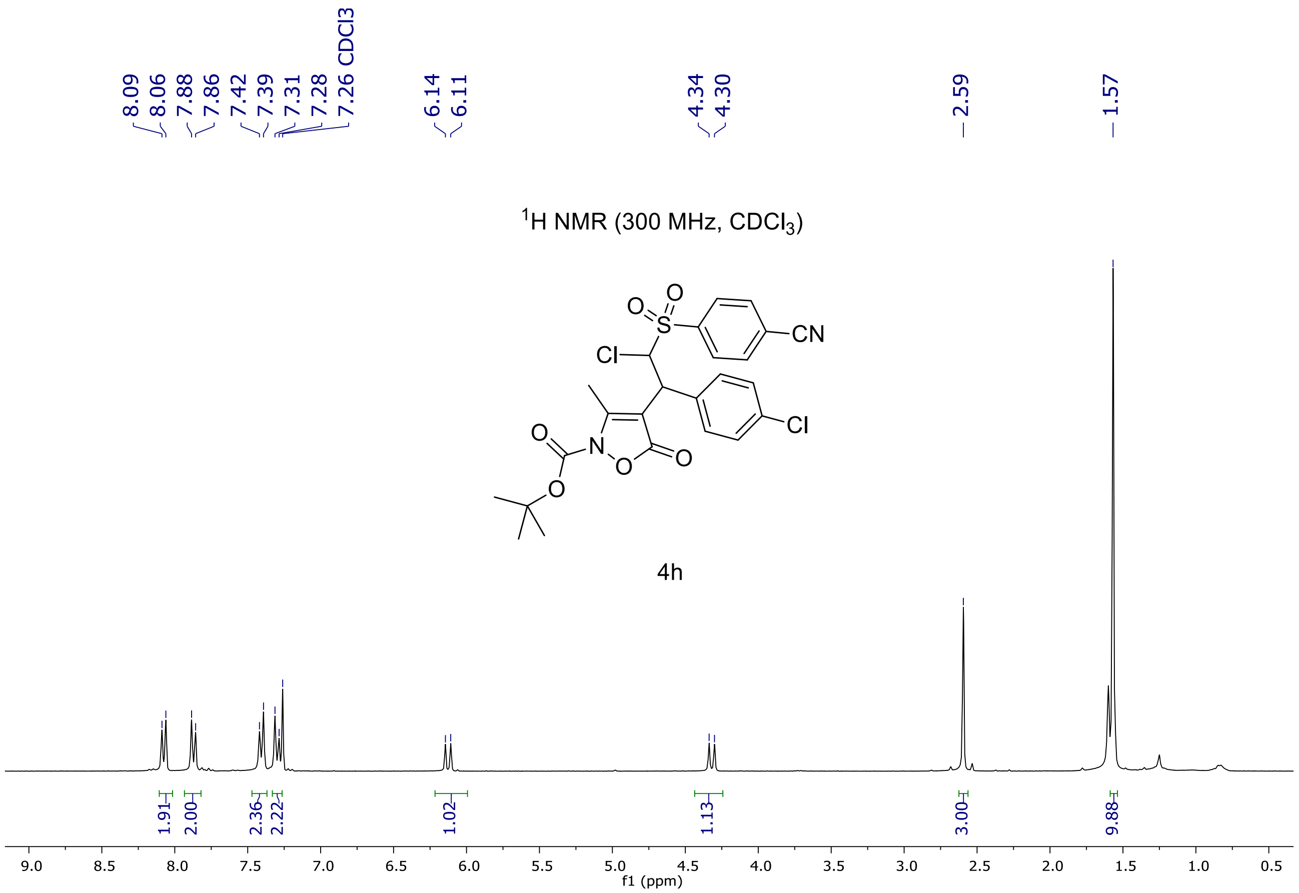
8.09
8.06
7.88
7.86
7.42
7.39
7.31
7.28
7.26 CDCl3
6.14
6.11
4.34
4.30
2.59
1.57
1H NMR (300 MHz, CDCl3)
4h
1.91
2.00
2.36
2.22
1.02
1.13
3.00
9.88
f1 (ppm)

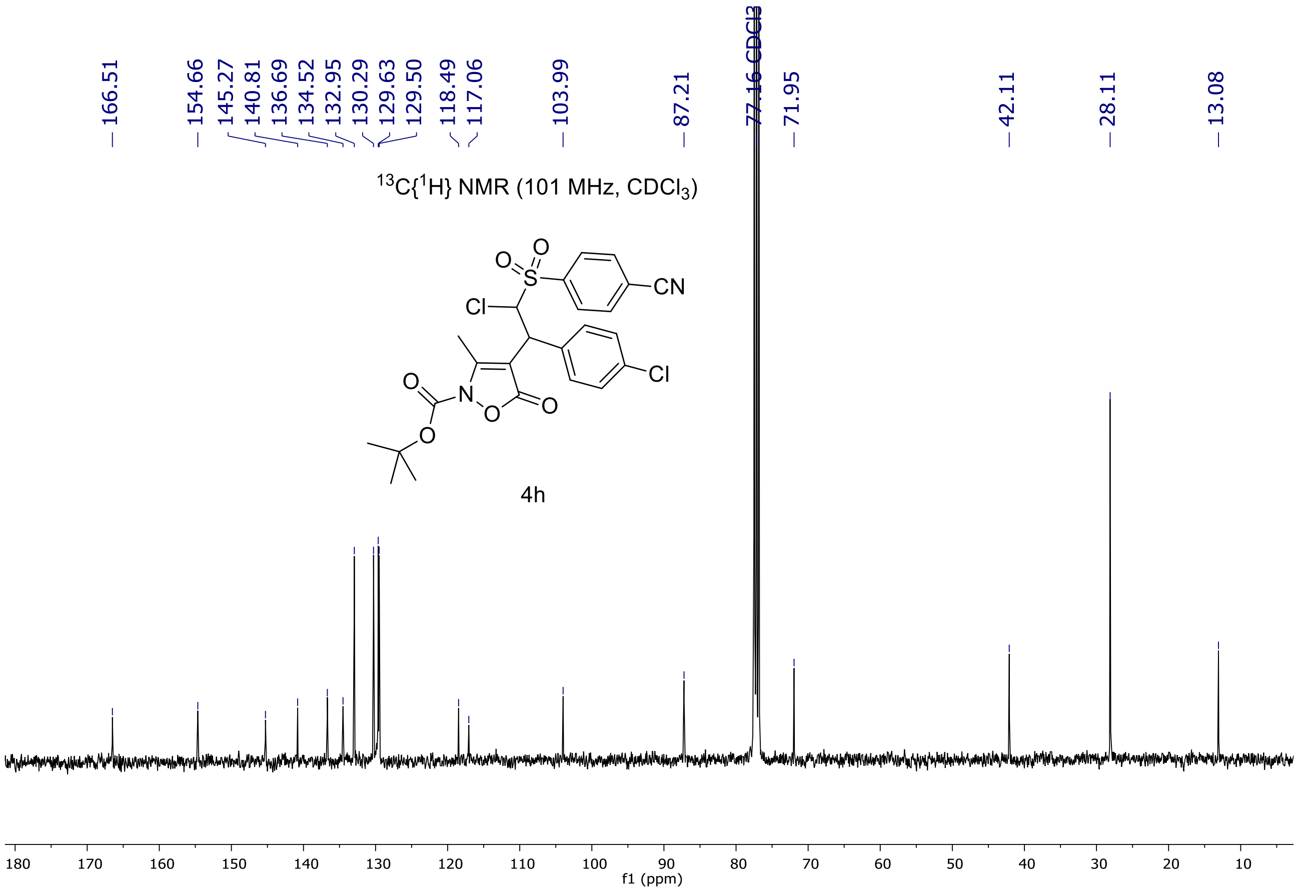
166.51
154.66
145.27
140.81
136.69
134.52
132.95
130.29
129.63
129.50
118.49
117.06
103.99
87.21
77.16 CDCl3
71.95
42.11
28.11
13.08
13C{1H} NMR (101 MHz, CDCl3)
4h
f1 (ppm)

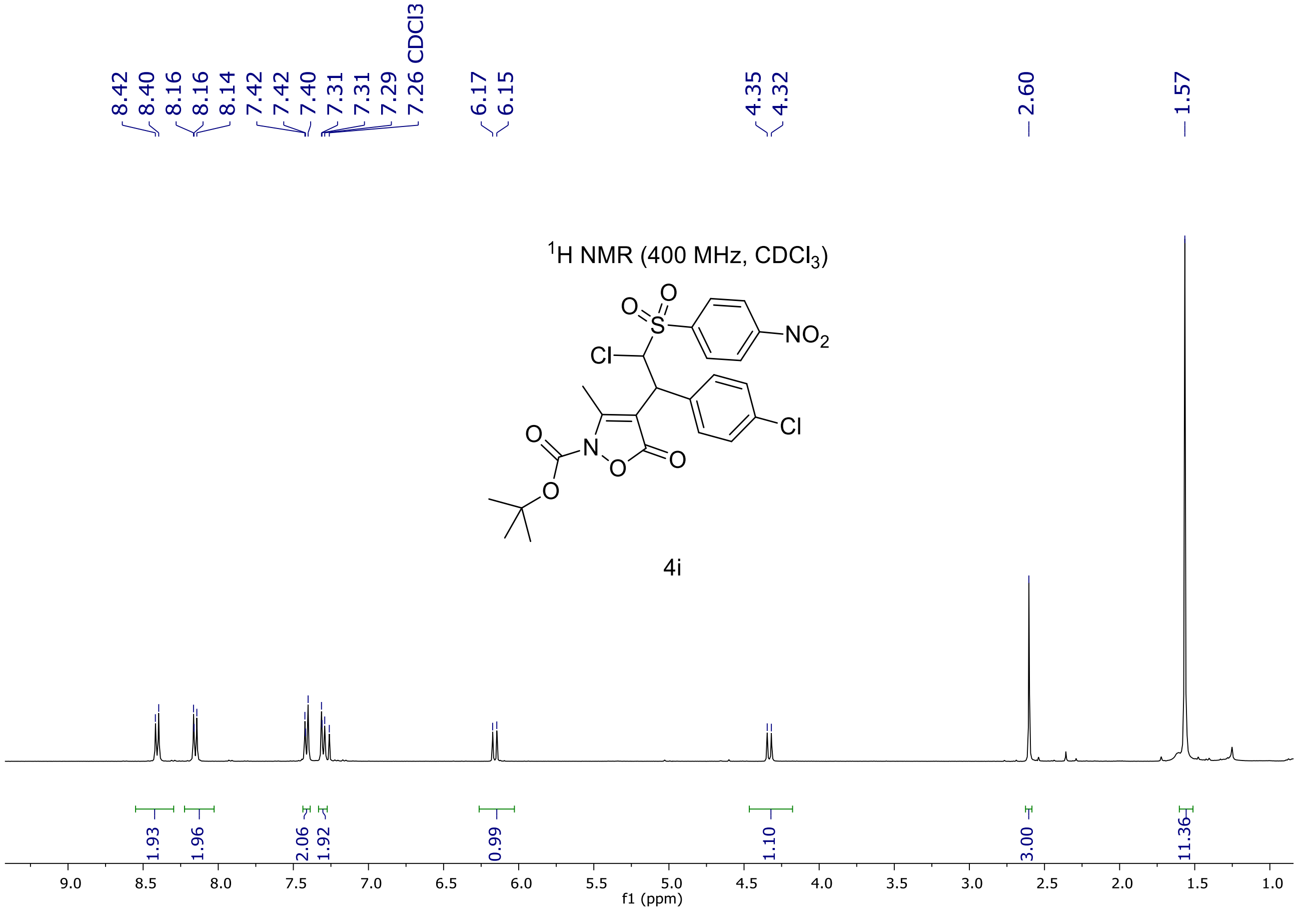
8.42
8.40
8.16
8.16
8.14
7.42
7.42
7.40
7.31
7.31
7.29
7.26 CDCl3
6.17
6.15
4.35
4.32
2.60
1.57
^{1}H NMR (400 MHz, $CDCl_3$)
O
O
S
NO_2
Cl
Cl
O
N
O
O
O
4i
1.93
1.96
2.06
1.92
0.99
1.10
3.00
11.36
9.0
8.5
8.0
7.5
7.0
6.5
6.0
5.5
5.0
4.5
4.0
3.5
3.0
2.5
2.0
1.5
1.0
f1 (ppm)

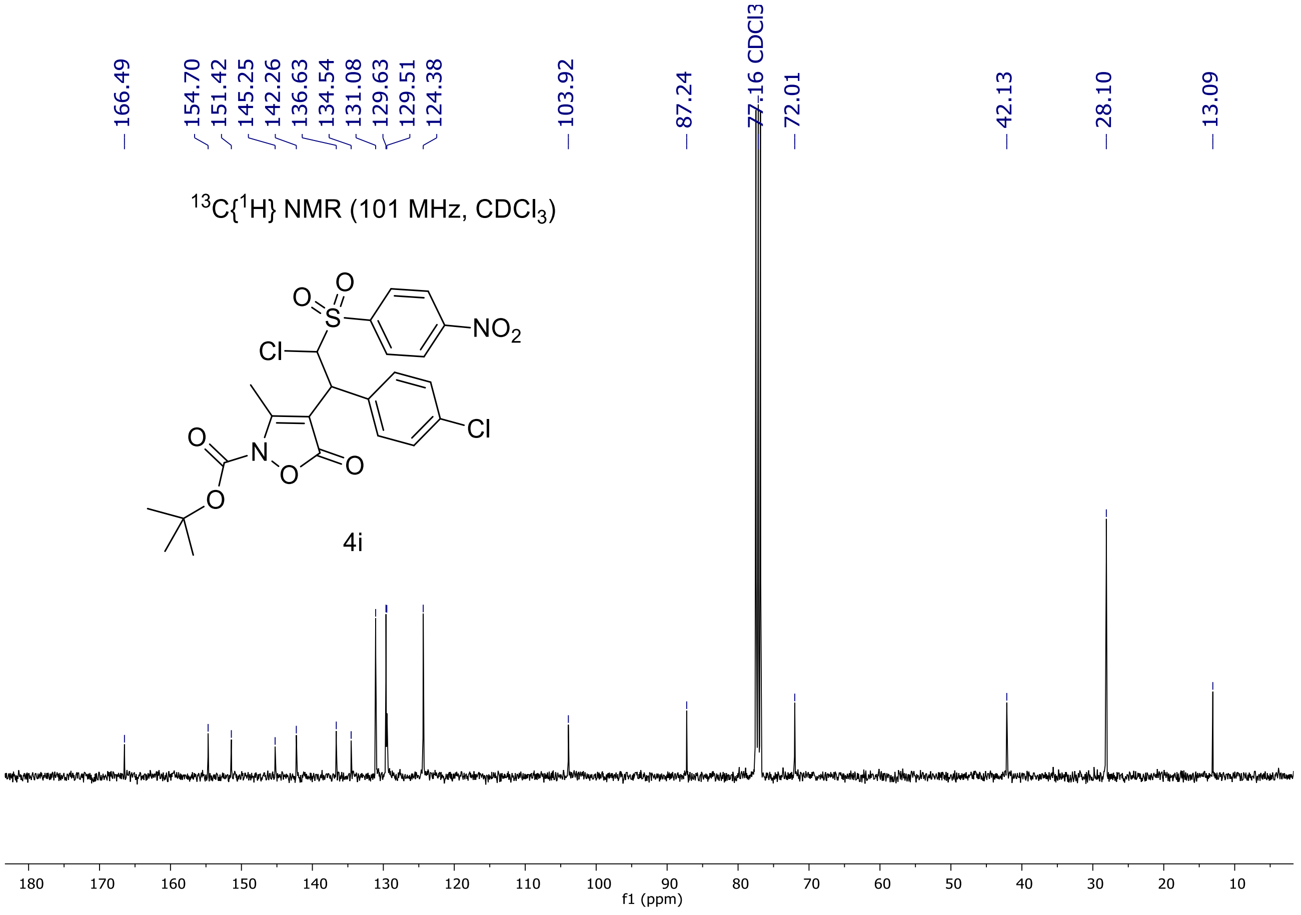
166.49
154.70
151.42
145.25
142.26
136.63
134.54
131.08
129.63
129.51
124.38
103.92
87.24
77.16 CDCl3
72.01
42.13
28.10
13.09
$^{13}C\{^{1}H\}$ NMR (101 MHz, $CDCl_3$)
O
O
S
NO_2
Cl
Cl
O
N
O
O
O
4i
180
170
160
150
140
130
120
110
100
90
80
70
60
50
40
30
20
10
f1 (ppm)

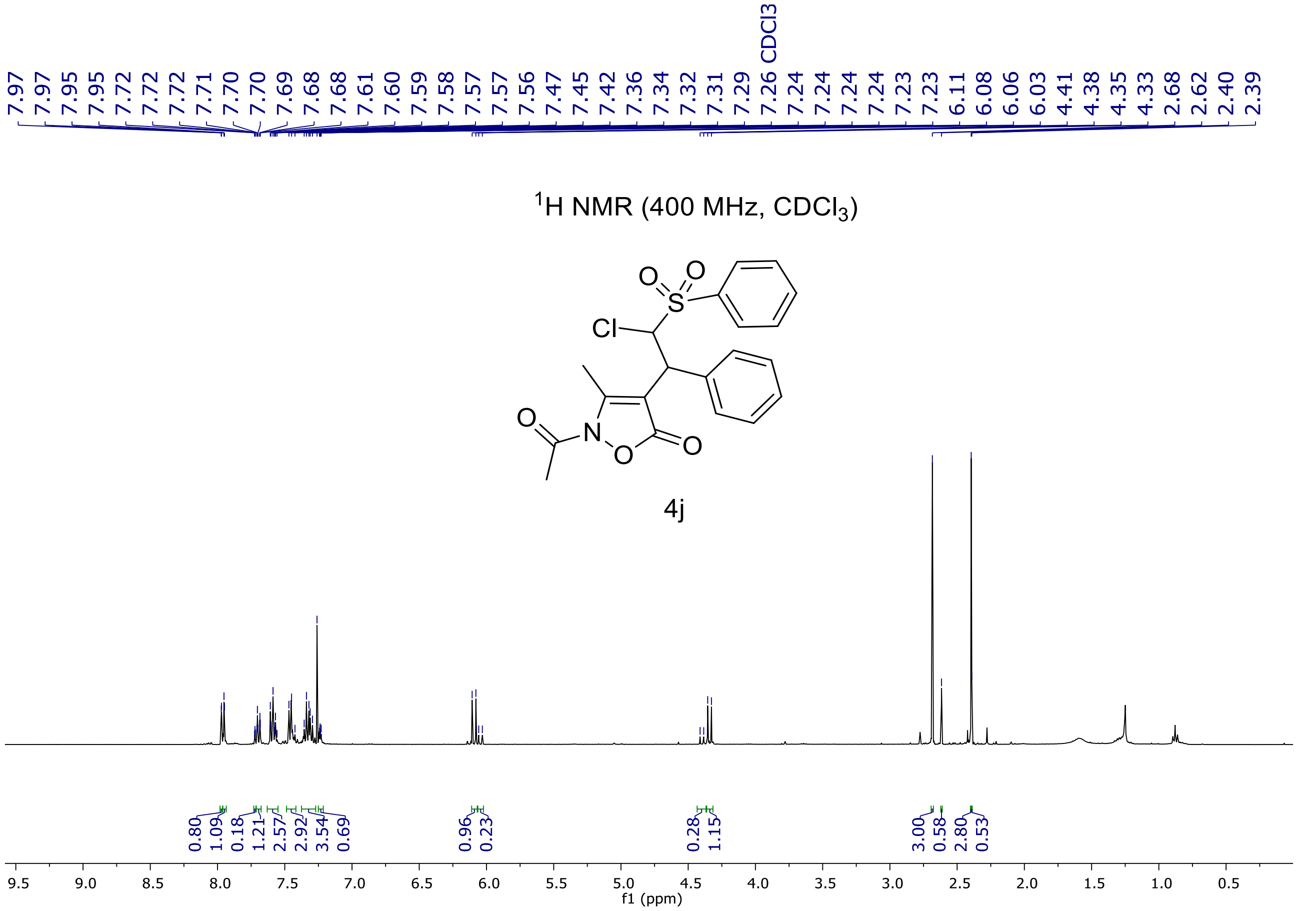
7.97 7.97 7.95 7.95 7.72 7.72 7.72 7.71 7.70 7.70 7.69 7.68 7.68 7.61 7.60 7.59 7.58 7.57 7.57 7.56 7.47 7.45 7.42 7.36 7.34 7.32 7.31 7.29 7.26 CDCl3 7.24 7.24 7.24 7.24 7.23 7.23 6.11 6.08 6.06 6.03 4.41 4.38 4.35 4.33 2.68 2.62 2.40 2.39
^{1}H NMR (400 MHz, $CDCl_3$)
O O S Cl N O O O
4j
0.80 1.09 0.18 1.21 2.57 2.92 3.54 0.69 0.96 0.23 0.28 1.15 3.00 0.58 2.80 0.53
9.5 9.0 8.5 8.0 7.5 7.0 6.5 6.0 5.5 5.0 4.5 4.0 3.5 3.0 2.5 2.0 1.5 1.0 0.5
f1 (ppm)

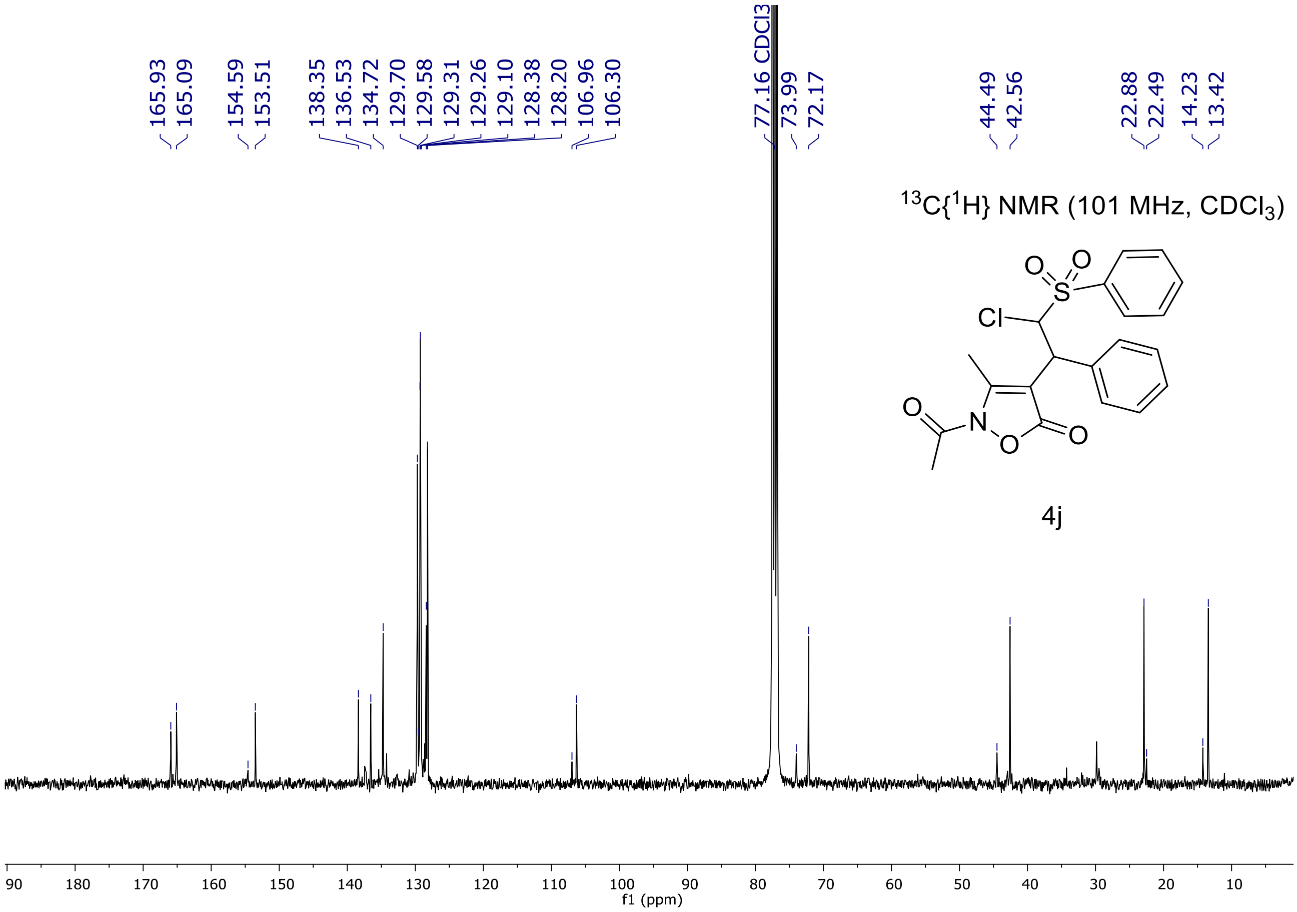
165.93 165.09 154.59 153.51 138.35 136.53 134.72 129.70 129.58 129.31 129.26 129.10 128.38 128.20 106.96 106.30 77.16 CDCl3 73.99 72.17 44.49 42.56 22.88 22.49 14.23 13.42
$^{13}C\{^1H\}$ NMR (101 MHz, $CDCl_3$)
O O S Cl N O O O
4j
90 180 170 160 150 140 130 120 110 100 90 80 70 60 50 40 30 20 10
f1 (ppm)

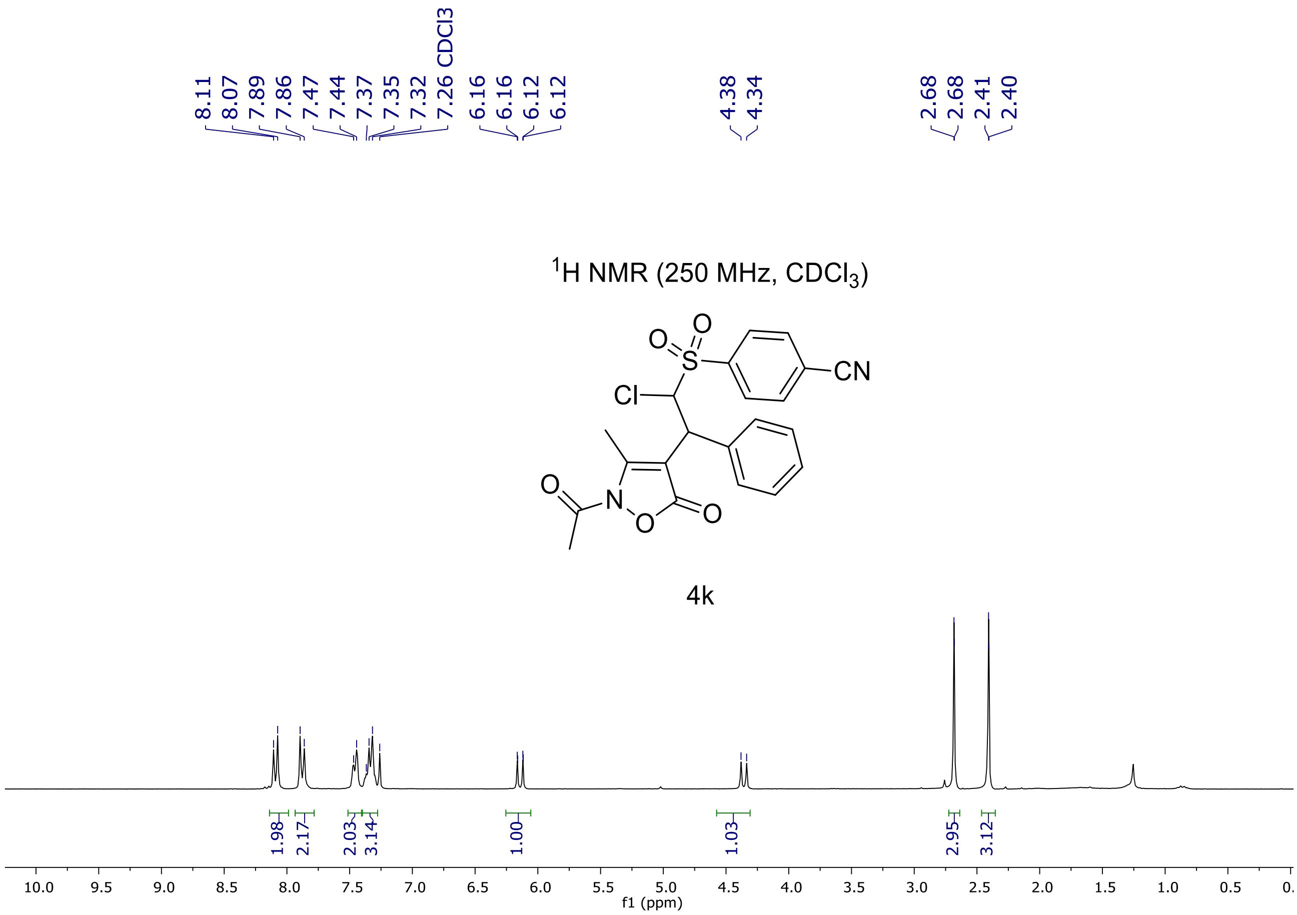
8.11
8.07
7.89
7.86
7.47
7.44
7.37
7.35
7.32
7.26 CDCl3
6.16
6.16
6.12
6.12
4.38
4.34
2.68
2.68
2.41
2.40
¹H NMR (250 MHz, CDCl₃)
4k
1.98
2.17
2.03
3.14
1.00
1.03
2.95
3.12
f1 (ppm)

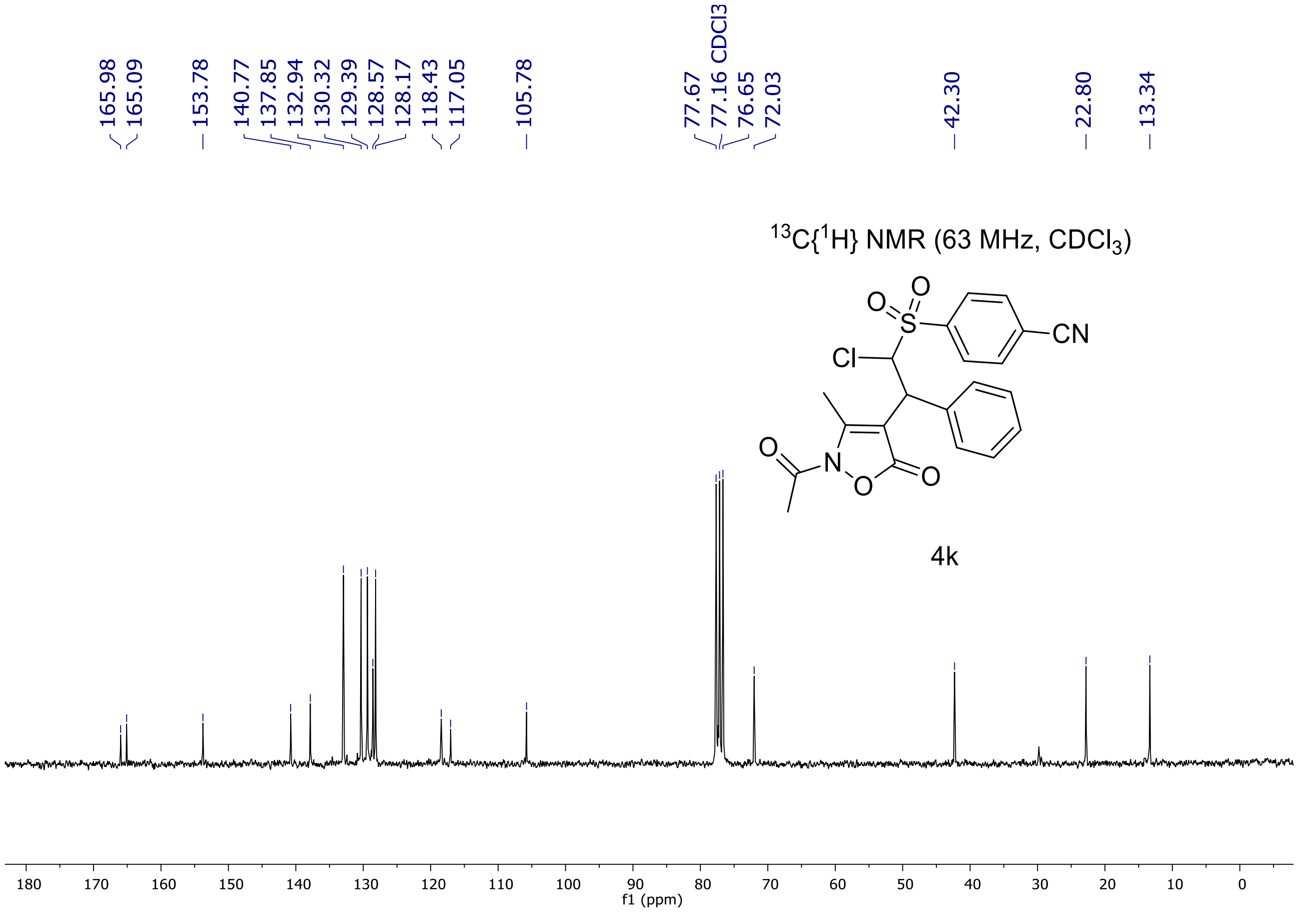
165.98
165.09
153.78
140.77
137.85
132.94
130.32
129.39
128.57
128.17
118.43
117.05
105.78
77.67
77.16 CDCl3
76.65
72.03
42.30
22.80
13.34
¹³C{¹H} NMR (63 MHz, CDCl₃)
4k
f1 (ppm)

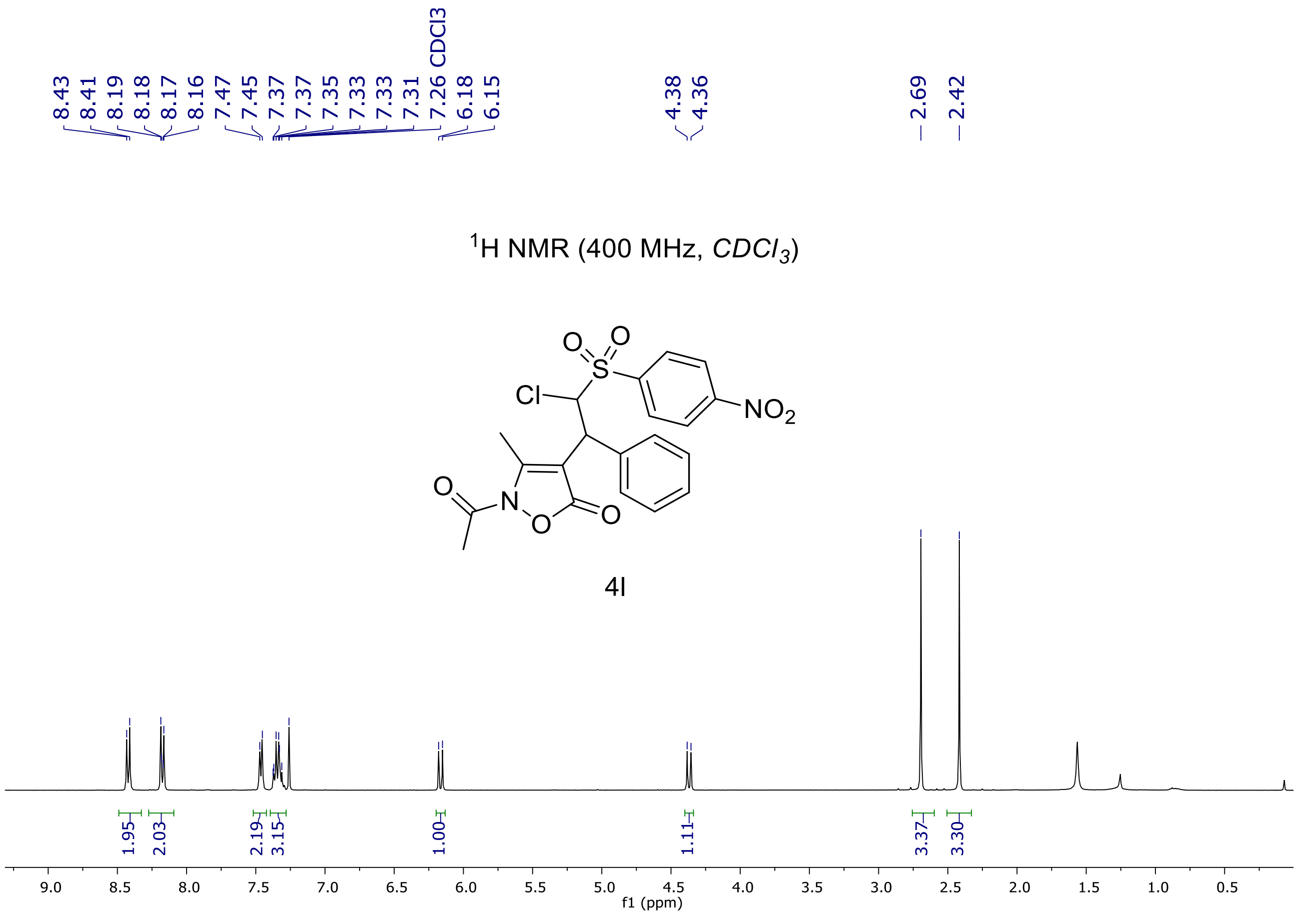
8.43
8.41
8.19
8.18
8.17
8.16
7.47
7.45
7.37
7.37
7.35
7.33
7.33
7.31
7.26 CDCl3
6.18
6.15
4.38
4.36
2.69
2.42
^{1}H NMR (400 MHz, $CDCl_3$)
Cl
O
S
O
NO_2
N
O
O
O
4l
1.95
2.03
2.19
3.15
1.00
1.11
3.37
3.30
9.0 8.5 8.0 7.5 7.0 6.5 6.0 5.5 5.0 4.5 4.0 3.5 3.0 2.5 2.0 1.5 1.0 0.5
f1 (ppm)

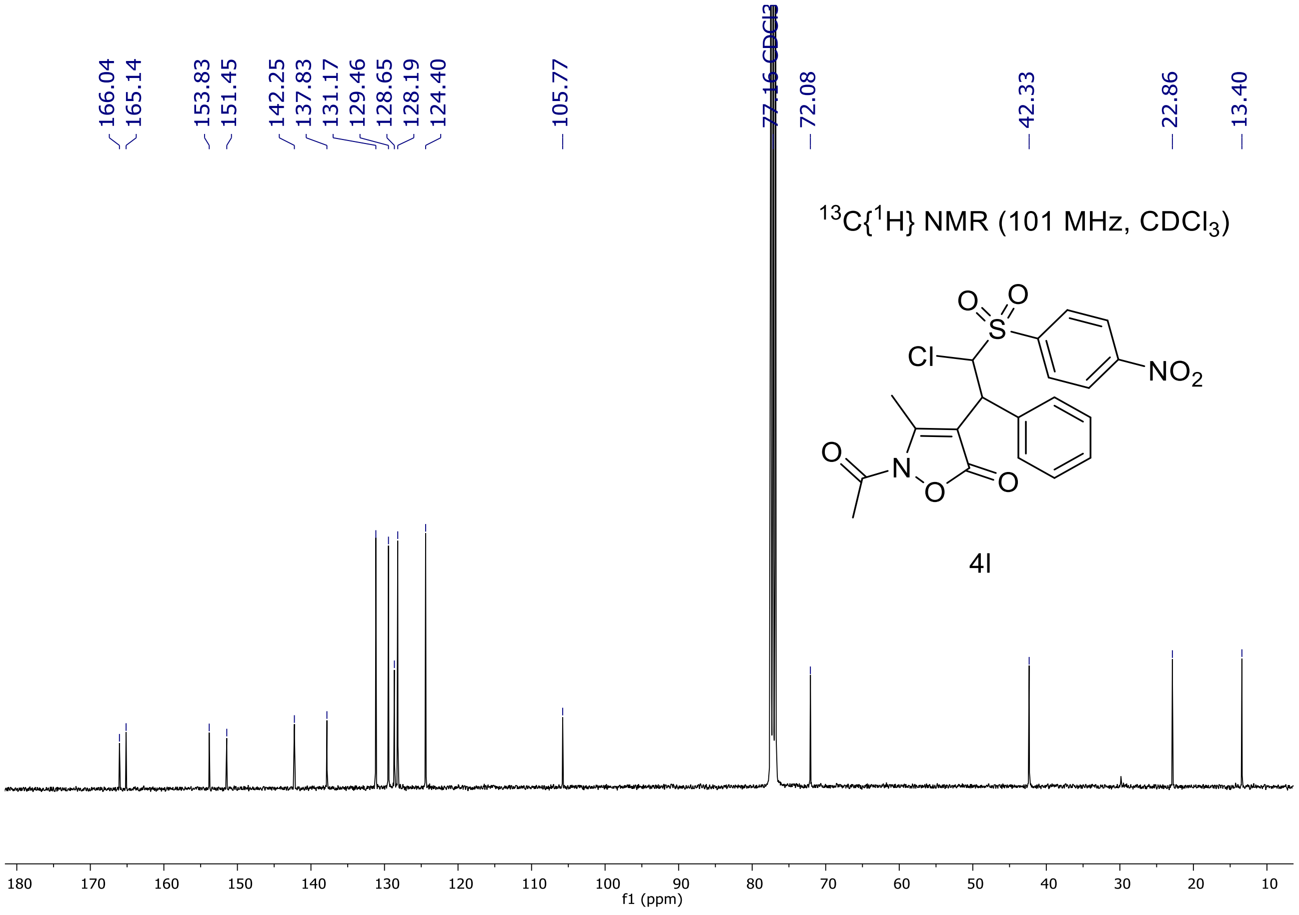
166.04
165.14
153.83
151.45
142.25
137.83
131.17
129.46
128.65
128.19
124.40
105.77
77.16 CDCl3
72.08
42.33
22.86
13.40
$^{13}C\{^1H\}$ NMR (101 MHz, $CDCl_3$)
Cl
O
S
O
NO_2
N
O
O
O
4l
180 170 160 150 140 130 120 110 100 90 80 70 60 50 40 30 20 10
f1 (ppm)

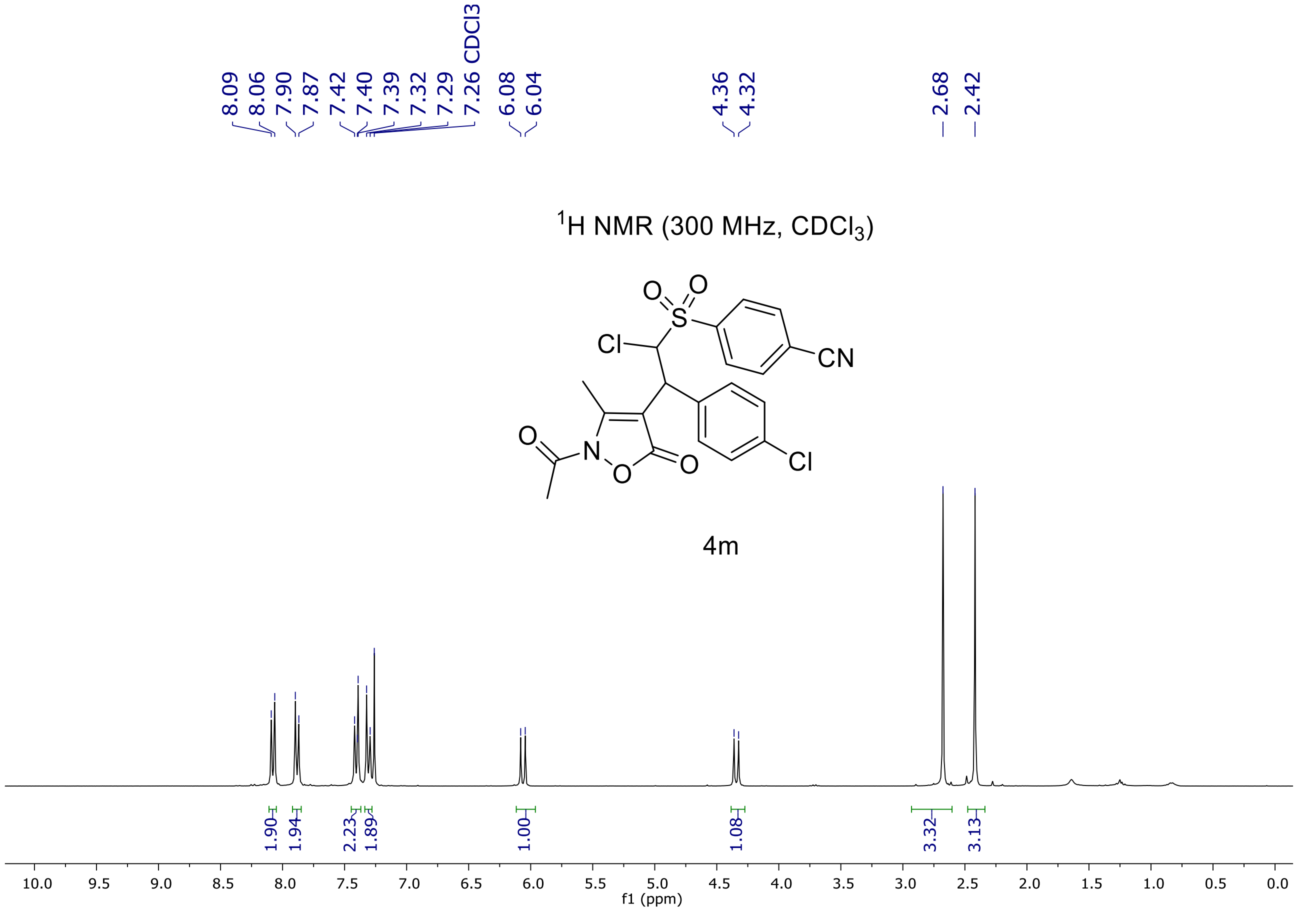
8.09
8.06
7.90
7.87
7.42
7.40
7.39
7.32
7.29
7.26 CDCl3
6.08
6.04
4.36
4.32
2.68
2.42
^{1}H NMR (300 MHz, $CDCl_3$)
O
O
S
Cl
CN
O
N
O
O
Cl
4m
1.90
1.94
2.23
1.89
1.00
1.08
3.32
3.13
10.0
9.5
9.0
8.5
8.0
7.5
7.0
6.5
6.0
5.5
5.0
4.5
4.0
3.5
3.0
2.5
2.0
1.5
1.0
0.5
0.0
f1 (ppm)

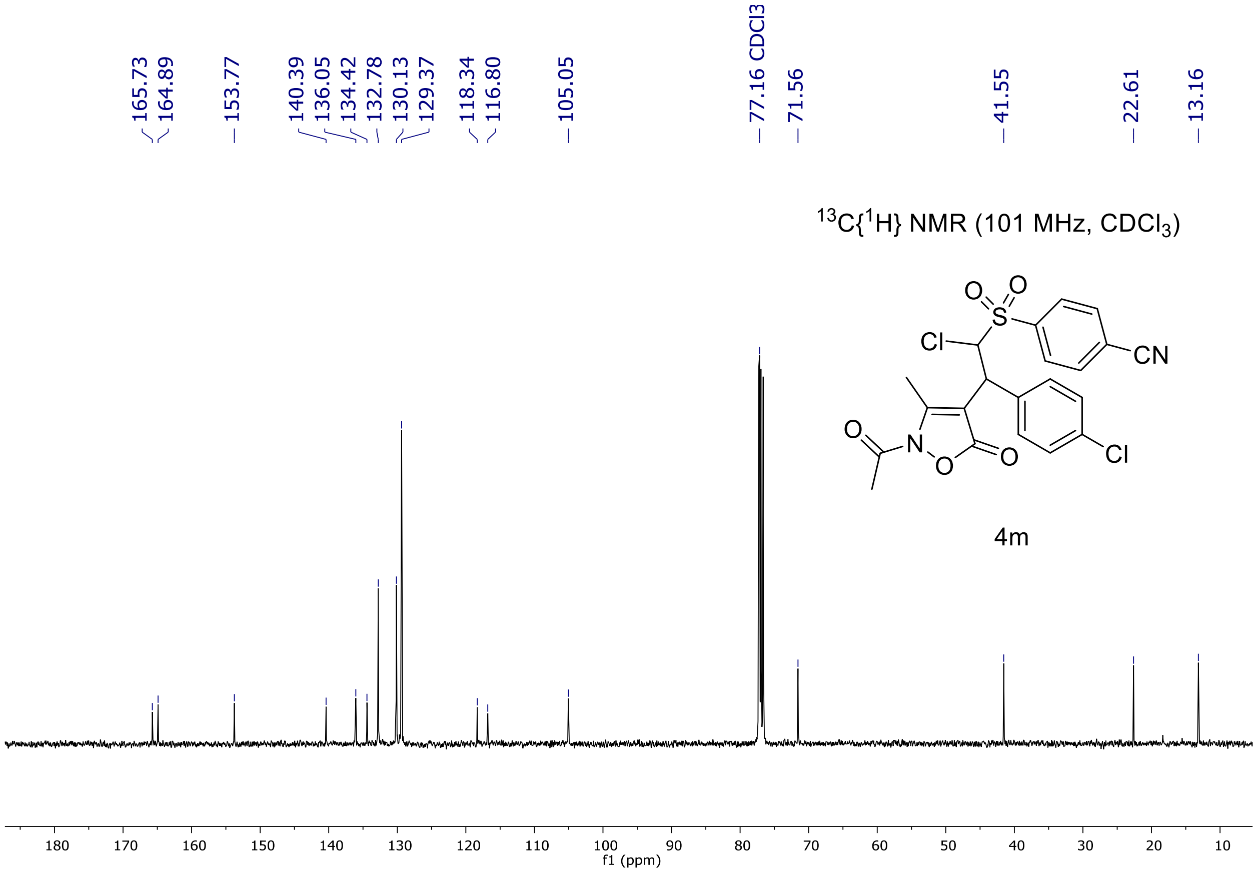
165.73
164.89
153.77
140.39
136.05
134.42
132.78
130.13
129.37
118.34
116.80
105.05
77.16 CDCl3
71.56
41.55
22.61
13.16
$^{13}C\{^1H\}$ NMR (101 MHz, $CDCl_3$)
O
O
S
Cl
CN
O
N
O
O
Cl
4m
180
170
160
150
140
130
120
110
100
90
80
70
60
50
40
30
20
10
f1 (ppm)

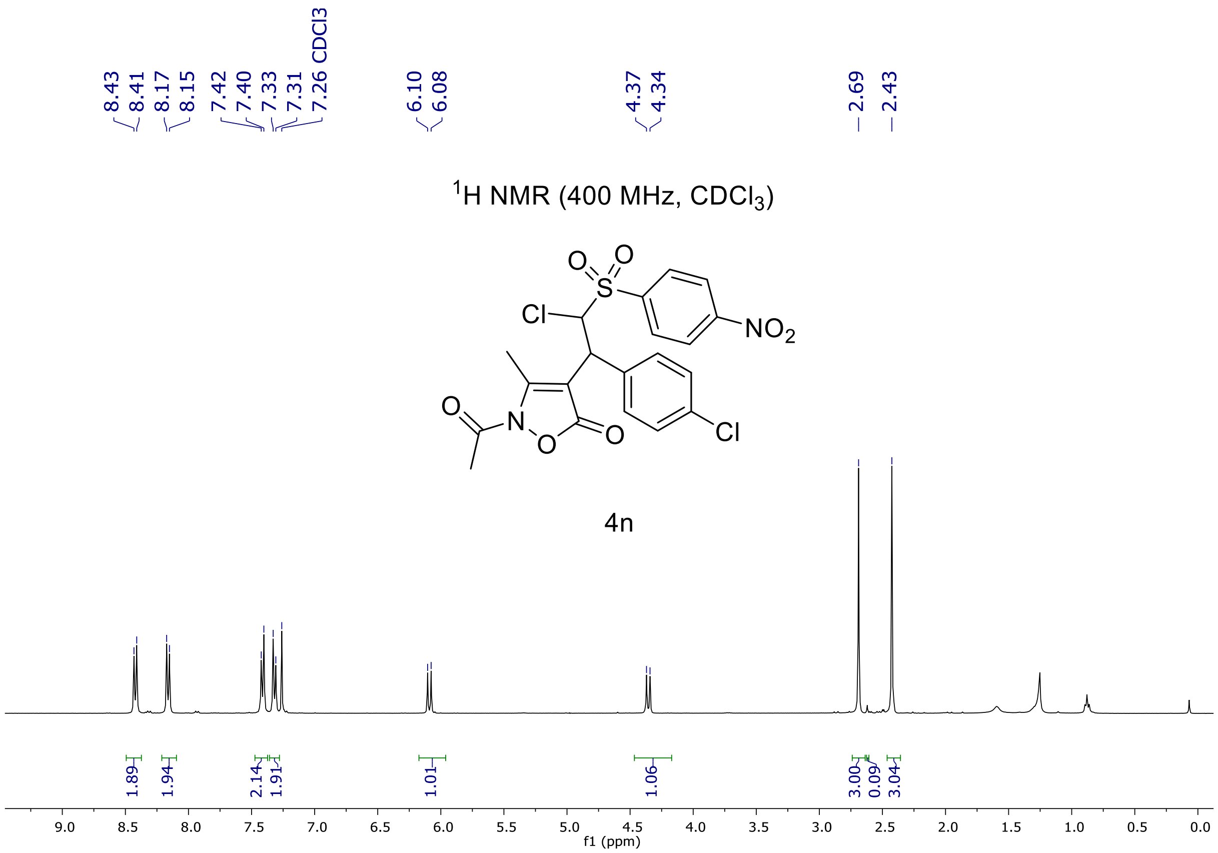
8.43
8.41
8.17
8.15
7.42
7.40
7.33
7.31
7.26 CDCl3
6.10
6.08
4.37
4.34
2.69
2.43
^{1}H NMR (400 MHz, $CDCl_3$)
Cl
O
S
O
NO_2
O
N
O
O
Cl
4n
1.89
1.94
2.14
1.91
1.01
1.06
3.00
0.09
3.04
f1 (ppm)

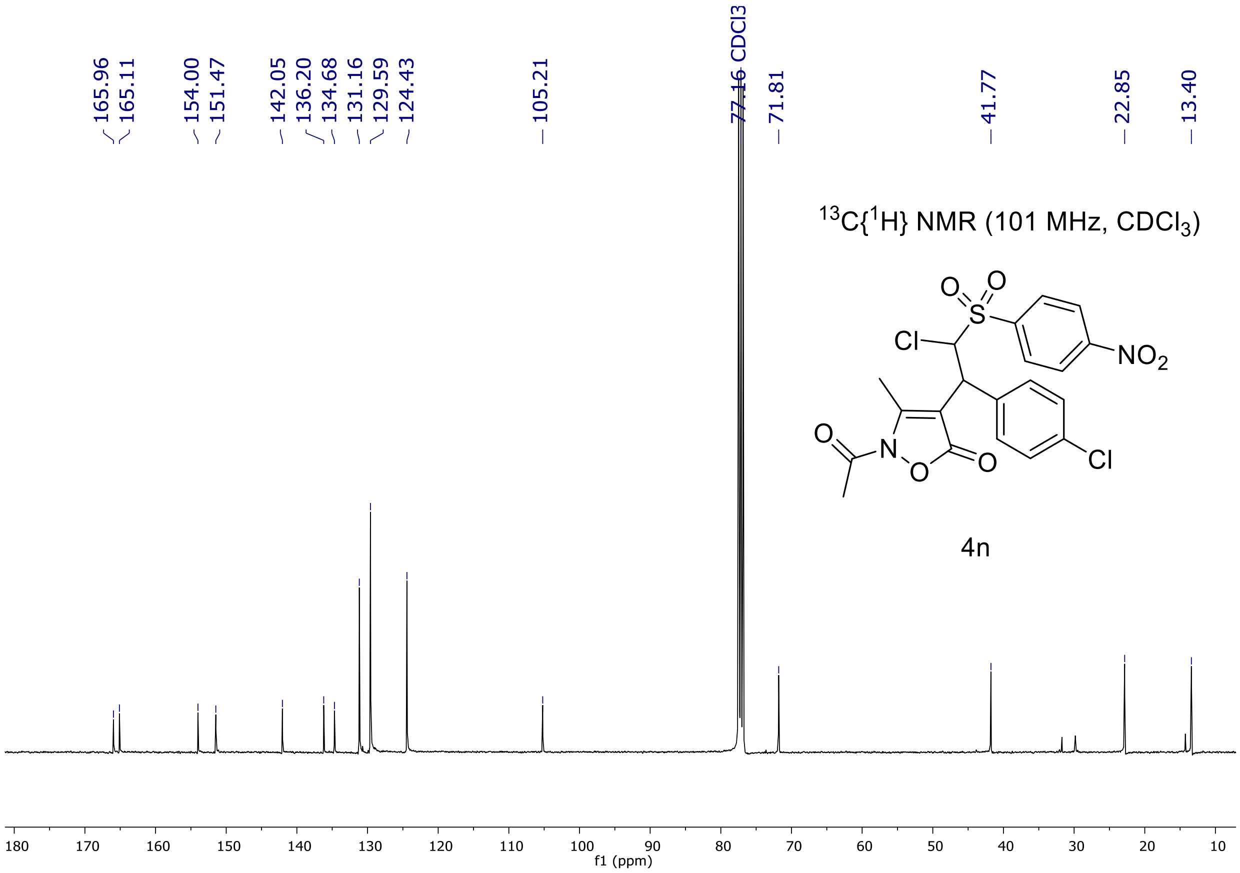
165.96
165.11
154.00
151.47
142.05
136.20
134.68
131.16
129.59
124.43
105.21
77.16 CDCl3
71.81
41.77
22.85
13.40
^{13}C{^{1}H} NMR (101 MHz, $CDCl_3$)
O
S
O
Cl
NO_2
O
N
O
O
Cl
4n
f1 (ppm)

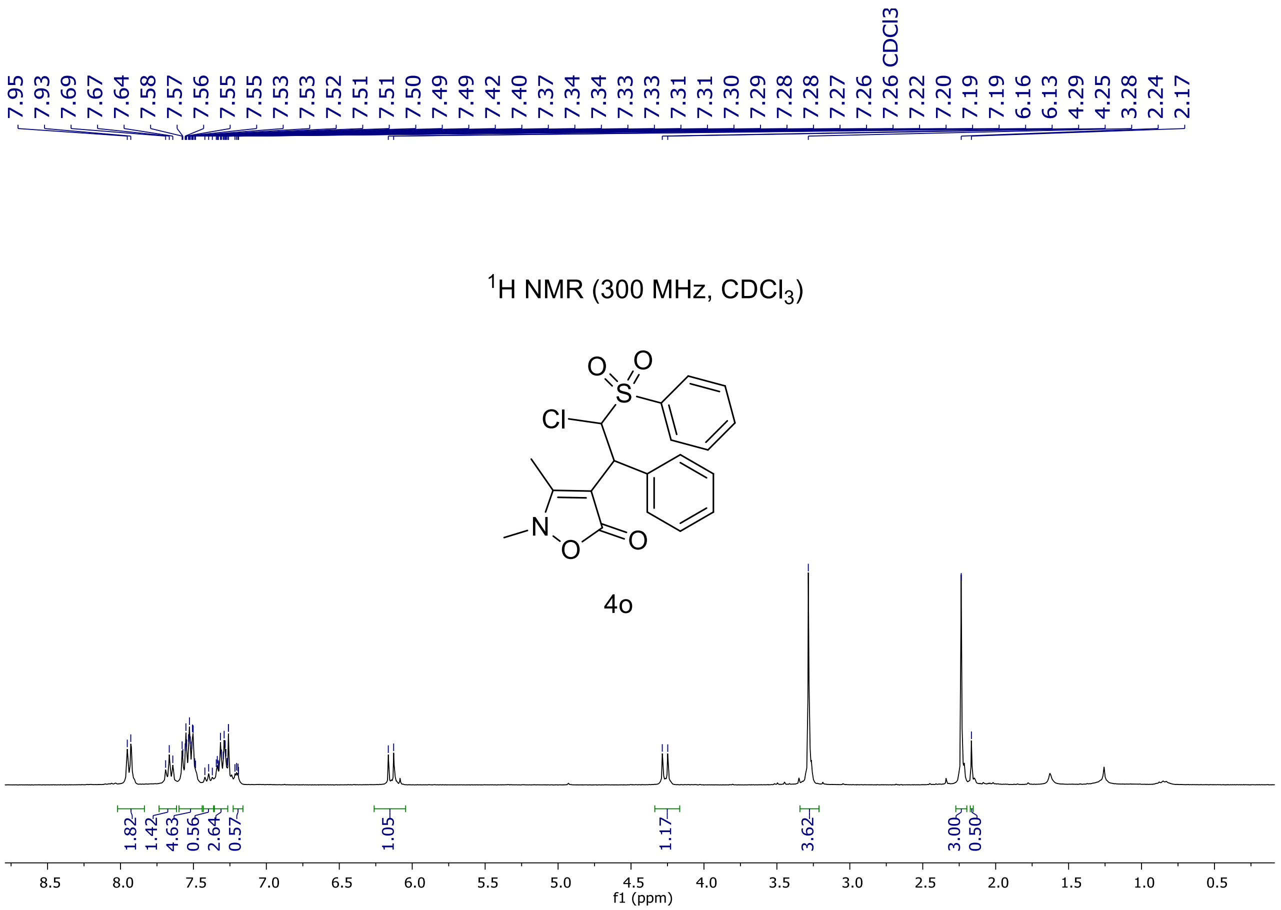

7.95 7.93 7.69 7.67 7.64 7.58 7.57 7.56 7.55 7.55 7.53 7.53 7.52 7.51 7.51 7.50 7.49 7.49 7.42 7.40 7.37 7.34 7.34 7.33 7.33 7.31 7.31 7.30 7.29 7.28 7.28 7.27 7.26 7.26 CDCl3 7.22 7.20 7.19 7.19 6.16 6.13 4.29 4.25 3.28 2.24 2.17
^{1}H NMR (300 MHz, CDCl$_3$)
4o
1.82 1.42 4.63 0.56 2.64 0.57 1.05 1.17 3.62 3.00 0.50
f1 (ppm)

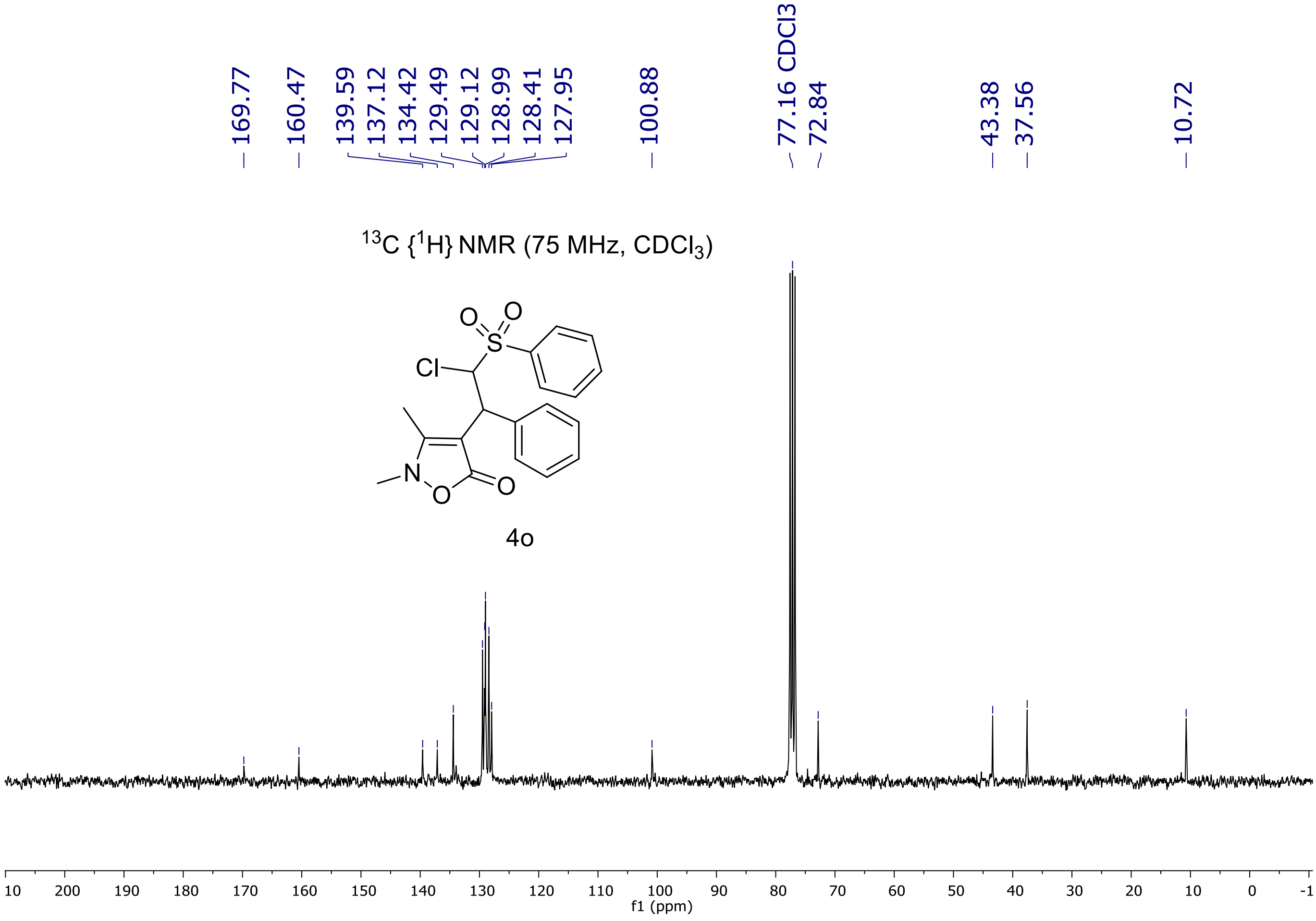

169.77 160.47 139.59 137.12 134.42 129.49 129.12 128.99 128.41 127.95 100.88 77.16 CDCl3 72.84 43.38 37.56 10.72
^{13}C {^{1}H} NMR (75 MHz, CDCl$_3$)
4o
f1 (ppm)

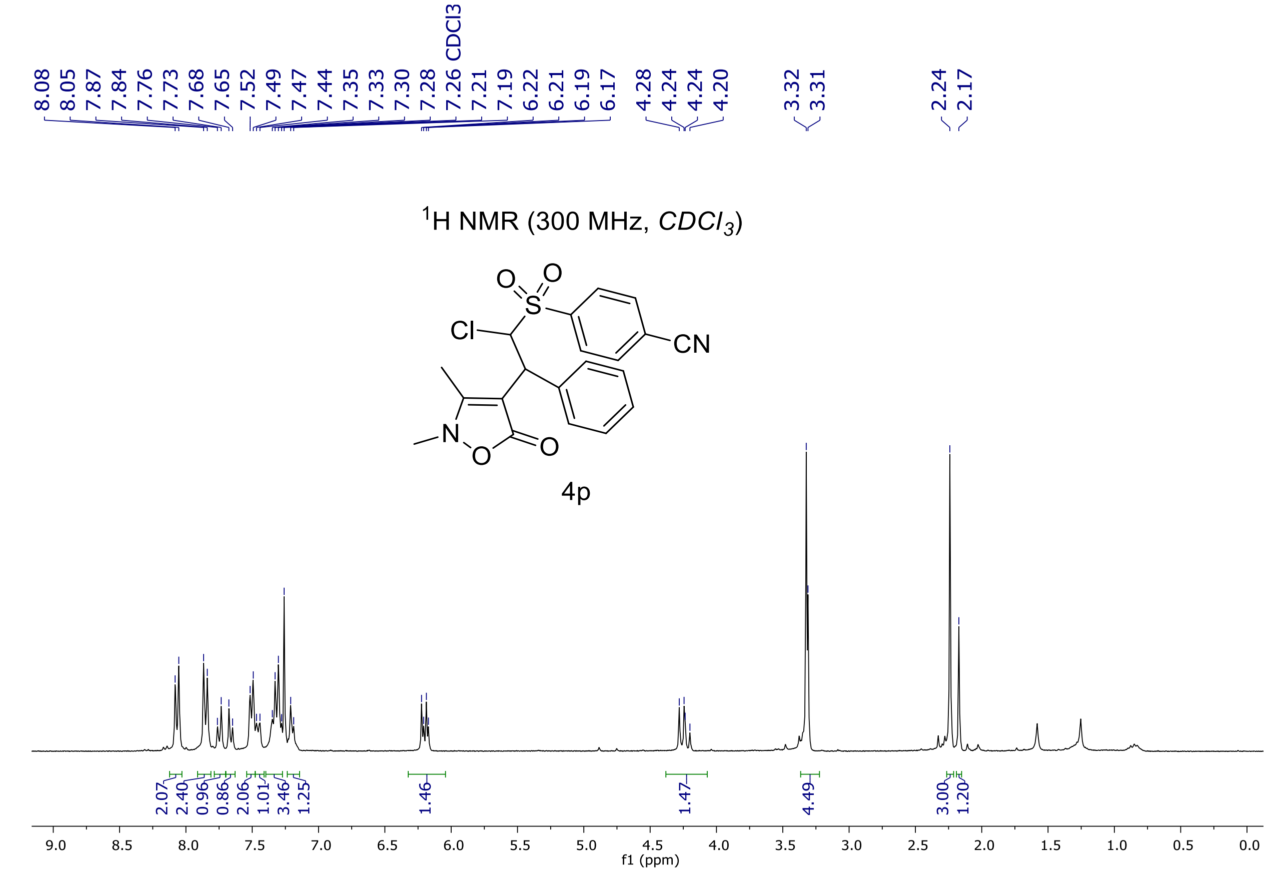
8.08 8.05 7.87 7.84 7.76 7.73 7.68 7.65 7.52 7.49 7.47 7.44 7.35 7.33 7.30 7.28 7.26 CDCl3 7.21 7.19 6.22 6.21 6.19 6.17 4.28 4.24 4.24 4.20 3.32 3.31 2.24 2.17
^{1}H NMR (300 MHz, $CDCl_3$)
O O S Cl CN N O O
4p
2.07 2.40 0.96 0.86 2.06 1.01 3.46 1.25 1.46 1.47 4.49 3.00 1.20
9.0 8.5 8.0 7.5 7.0 6.5 6.0 5.5 5.0 4.5 4.0 3.5 3.0 2.5 2.0 1.5 1.0 0.5 0.0
f1 (ppm)

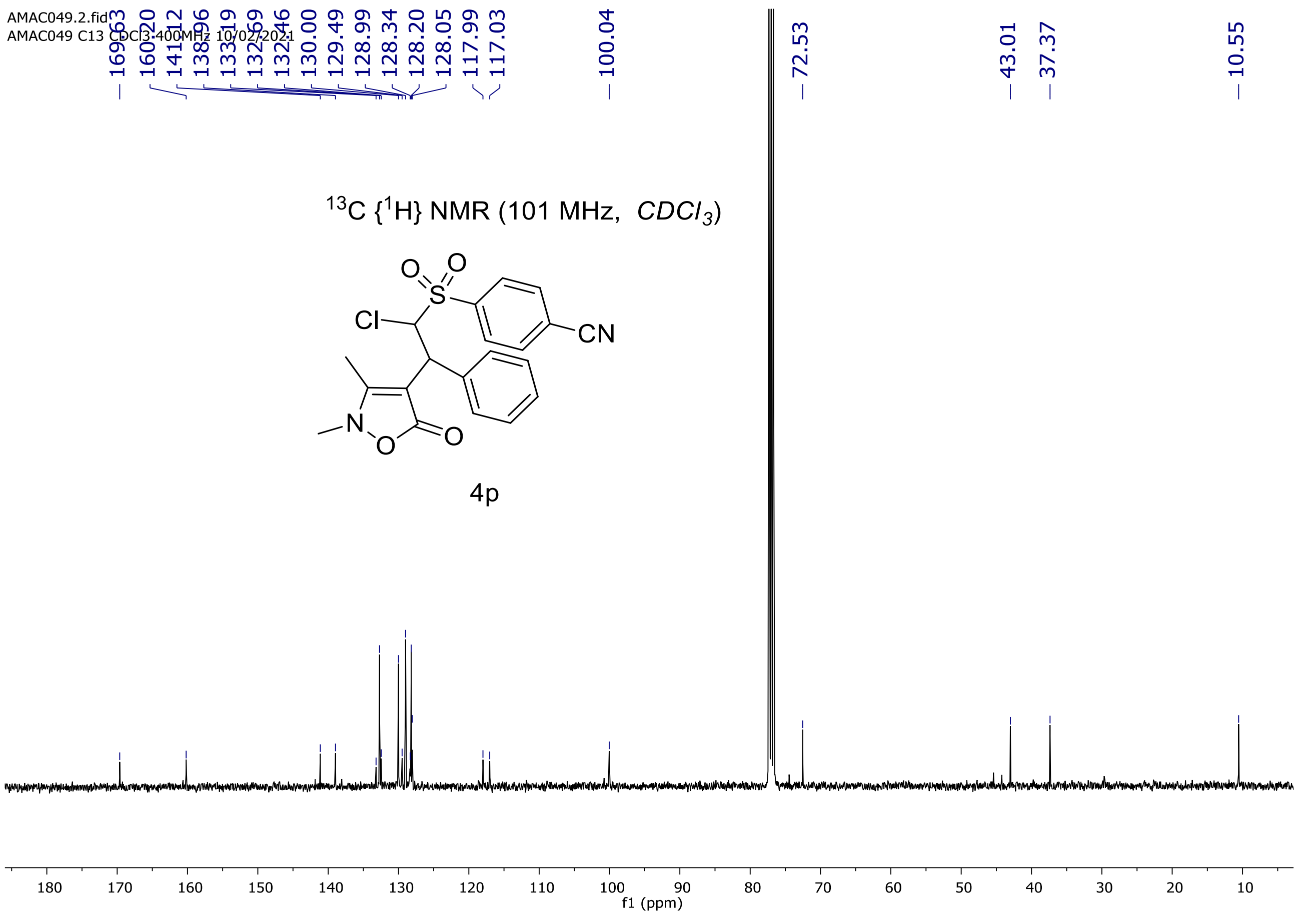
AMAC049.2.fid
AMAC049 C13 CDCl3-400MHz 10/02/2021
169.63 160.20 141.12 138.96 133.19 132.69 132.46 130.00 129.49 128.99 128.34 128.20 128.05 117.99 117.03 100.04 72.53 43.01 37.37 10.55
^{13}C {^{1}H} NMR (101 MHz, $CDCl_3$)
O O S Cl CN N O O
4p
180 170 160 150 140 130 120 110 100 90 80 70 60 50 40 30 20 10
f1 (ppm)

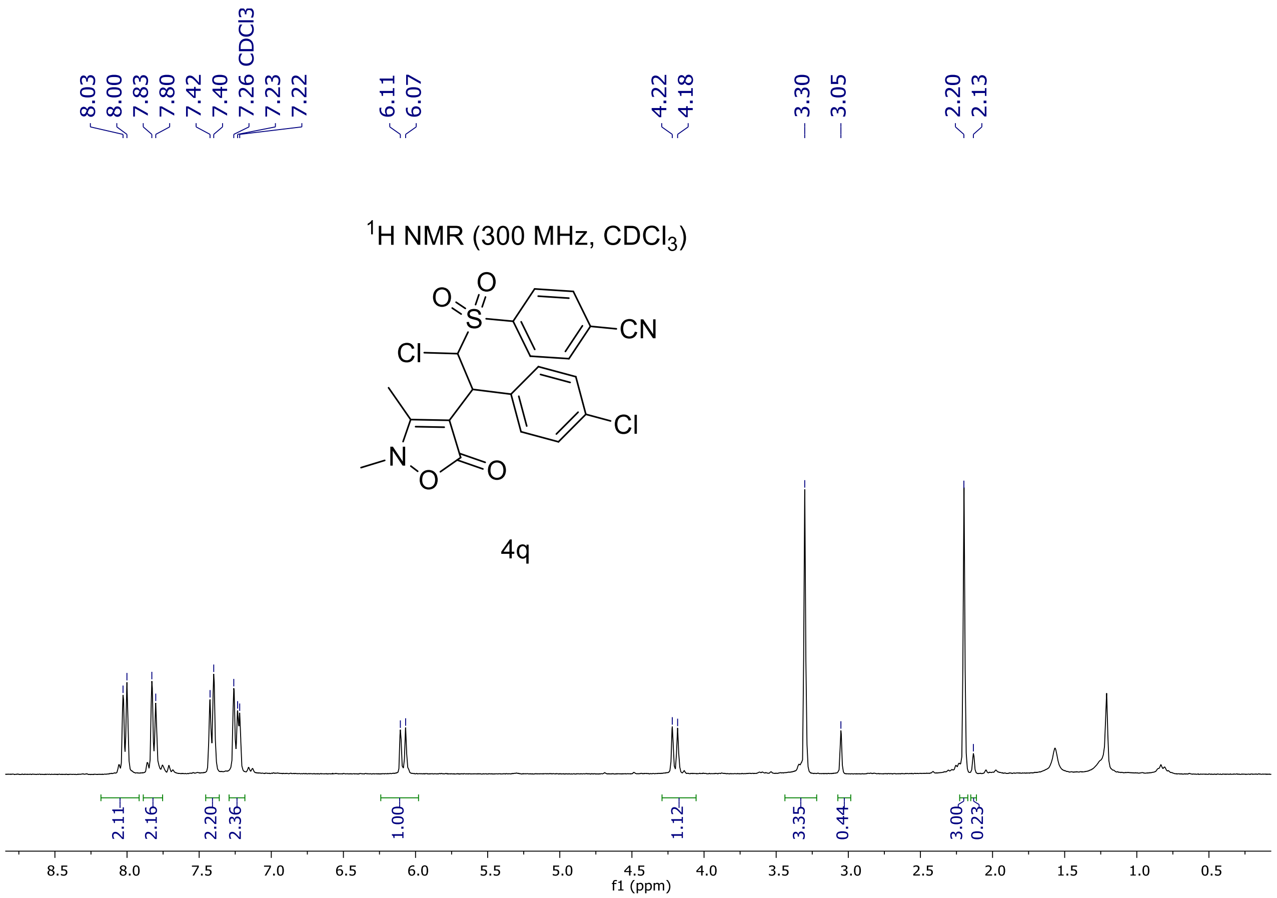
8.03
8.00
7.83
7.80
7.42
7.40
7.26 CDCl3
7.23
7.22
6.11
6.07
4.22
4.18
3.30
3.05
2.20
2.13
1H NMR (300 MHz, CDCl3)
CN
Cl
Cl
4q
2.11
2.16
2.20
2.36
1.00
1.12
3.35
0.44
3.00
0.23
f1 (ppm)

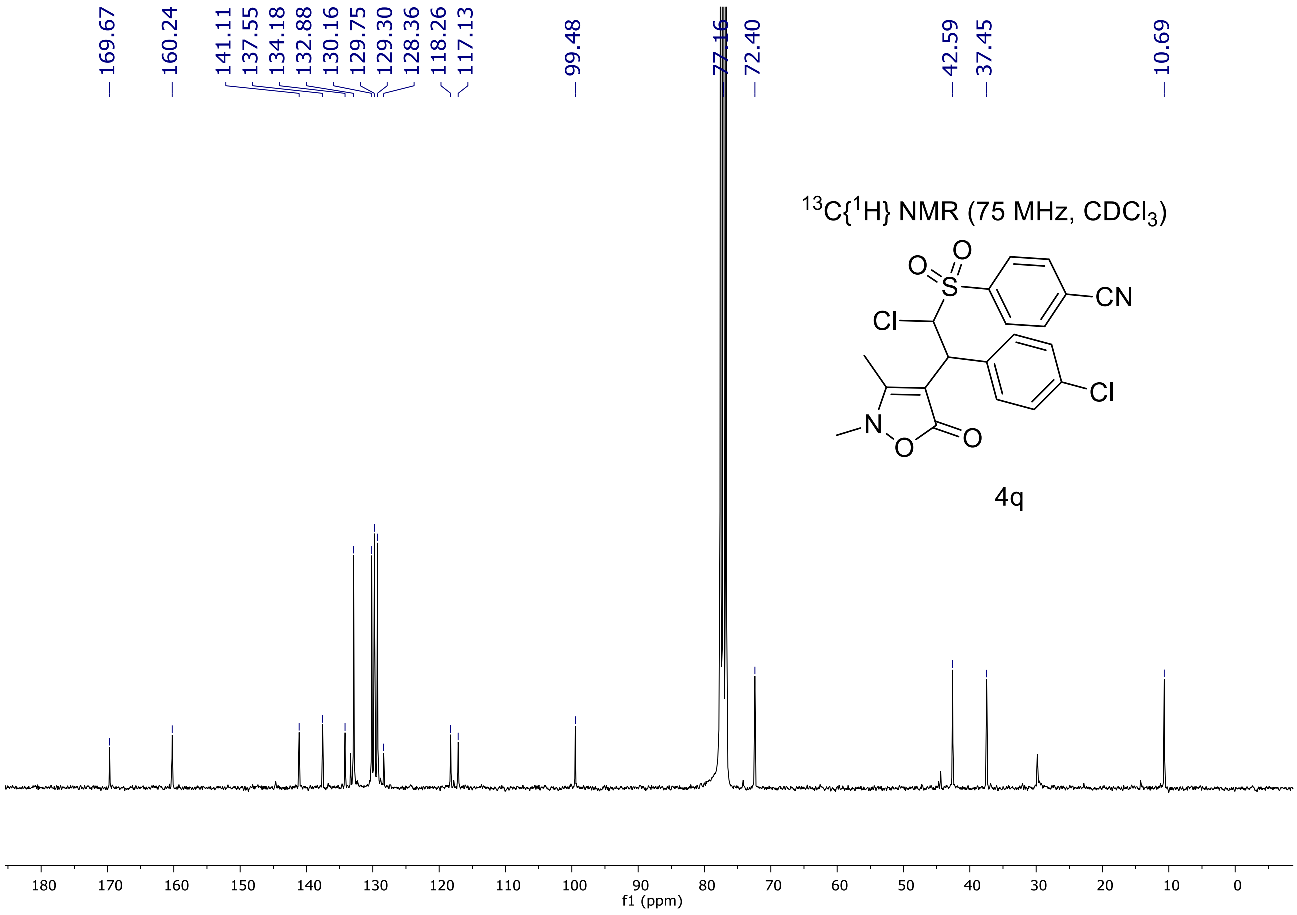
169.67
160.24
141.11
137.55
134.18
132.88
130.16
129.75
129.30
128.36
118.26
117.13
99.48
77.16
72.40
42.59
37.45
10.69
13C{1H} NMR (75 MHz, CDCl3)
CN
Cl
Cl
4q
f1 (ppm)

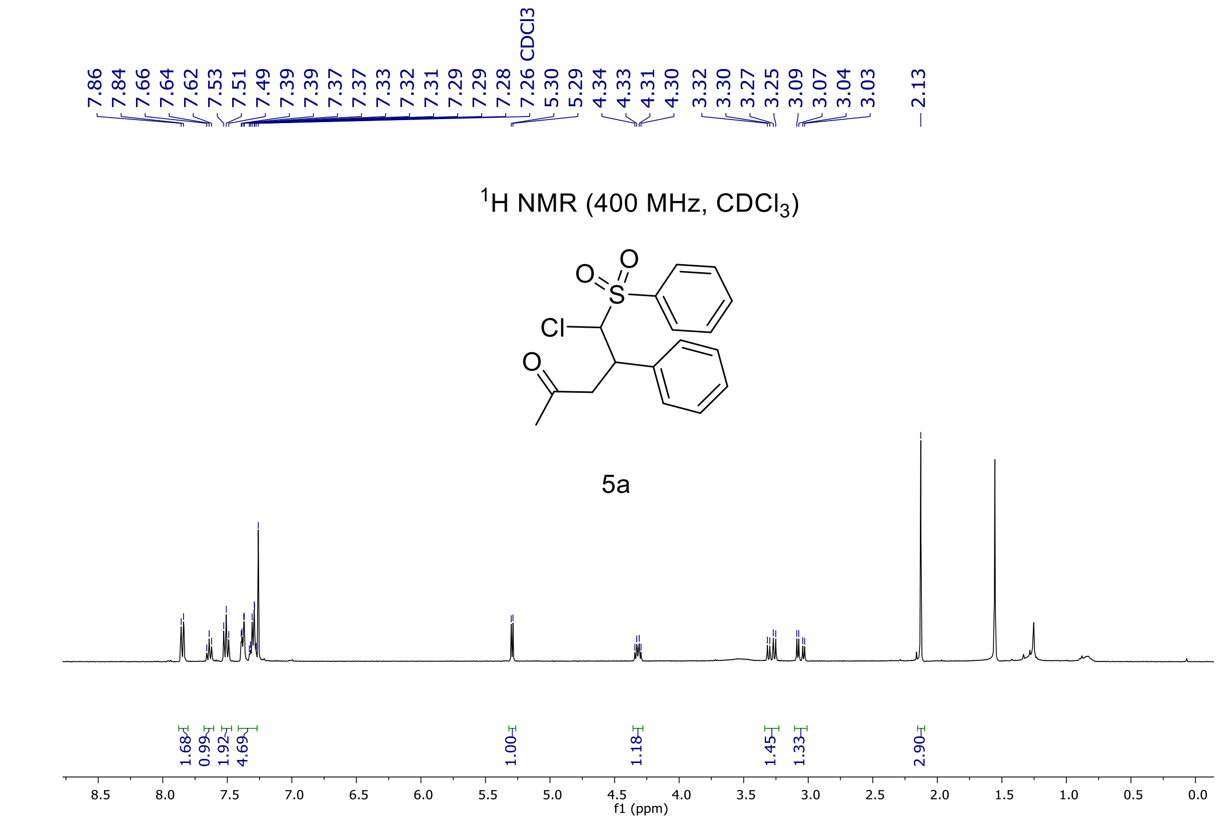
7.86 7.84 7.66 7.64 7.62 7.53 7.51 7.49 7.39 7.39 7.37 7.37 7.33 7.32 7.31 7.29 7.29 7.28 7.26 CDCl3 5.30 5.29 4.34 4.33 4.31 4.30 3.32 3.30 3.27 3.25 3.09 3.07 3.04 3.03 2.13
1H NMR (400 MHz, CDCl3)
O
O
S
Cl
O
5a
1.68 0.99 1.92 4.69 1.00 1.18 1.45 1.33 2.90
8.5 8.0 7.5 7.0 6.5 6.0 5.5 5.0 4.5 4.0 3.5 3.0 2.5 2.0 1.5 1.0 0.5 0.0
f1 (ppm)

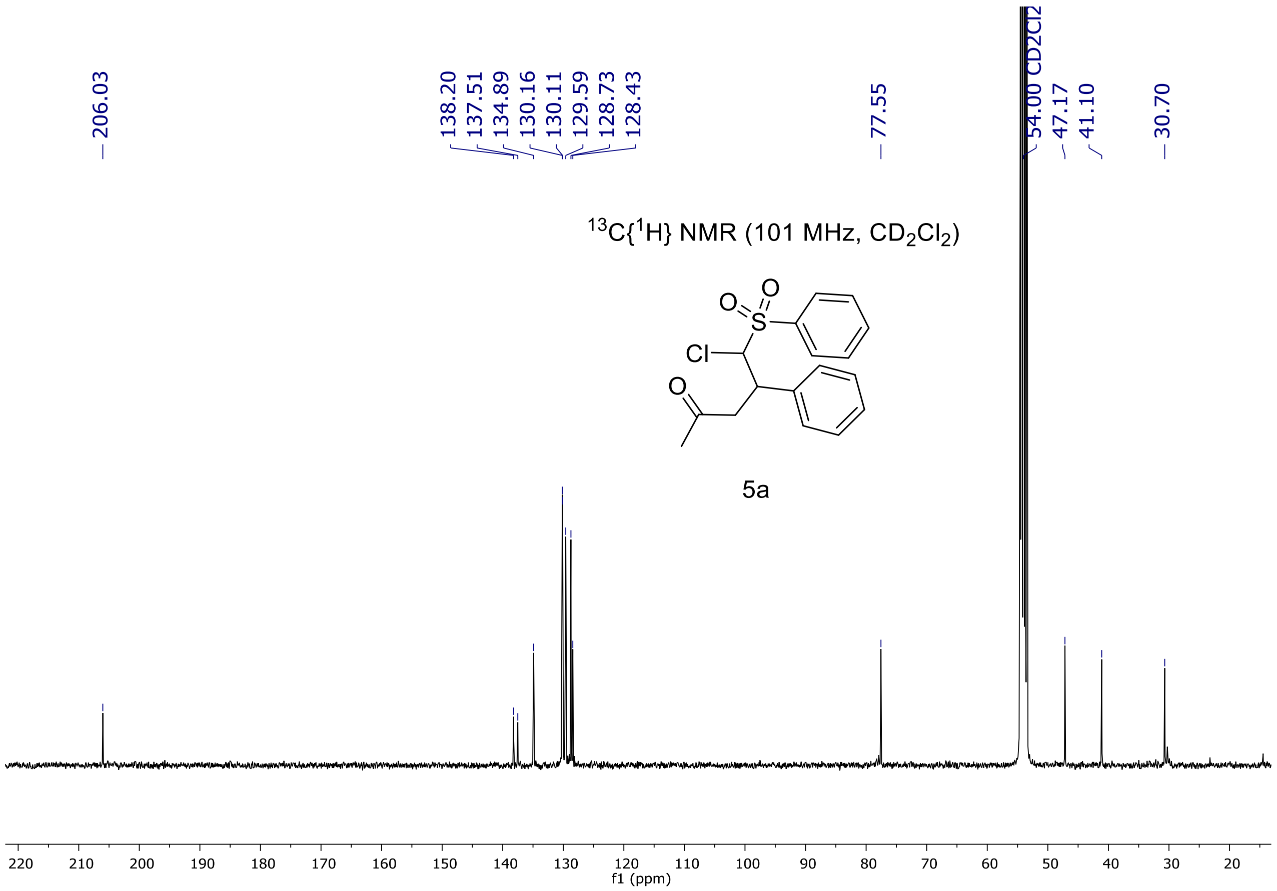
206.03
138.20 137.51 134.89 130.16 130.11 129.59 128.73 128.43
77.55
54.00 CD2Cl2 47.17 41.10 30.70
13C{1H} NMR (101 MHz, CD2Cl2)
O
O
S
Cl
O
5a
220 210 200 190 180 170 160 150 140 130 120 110 100 90 80 70 60 50 40 30 20
f1 (ppm)

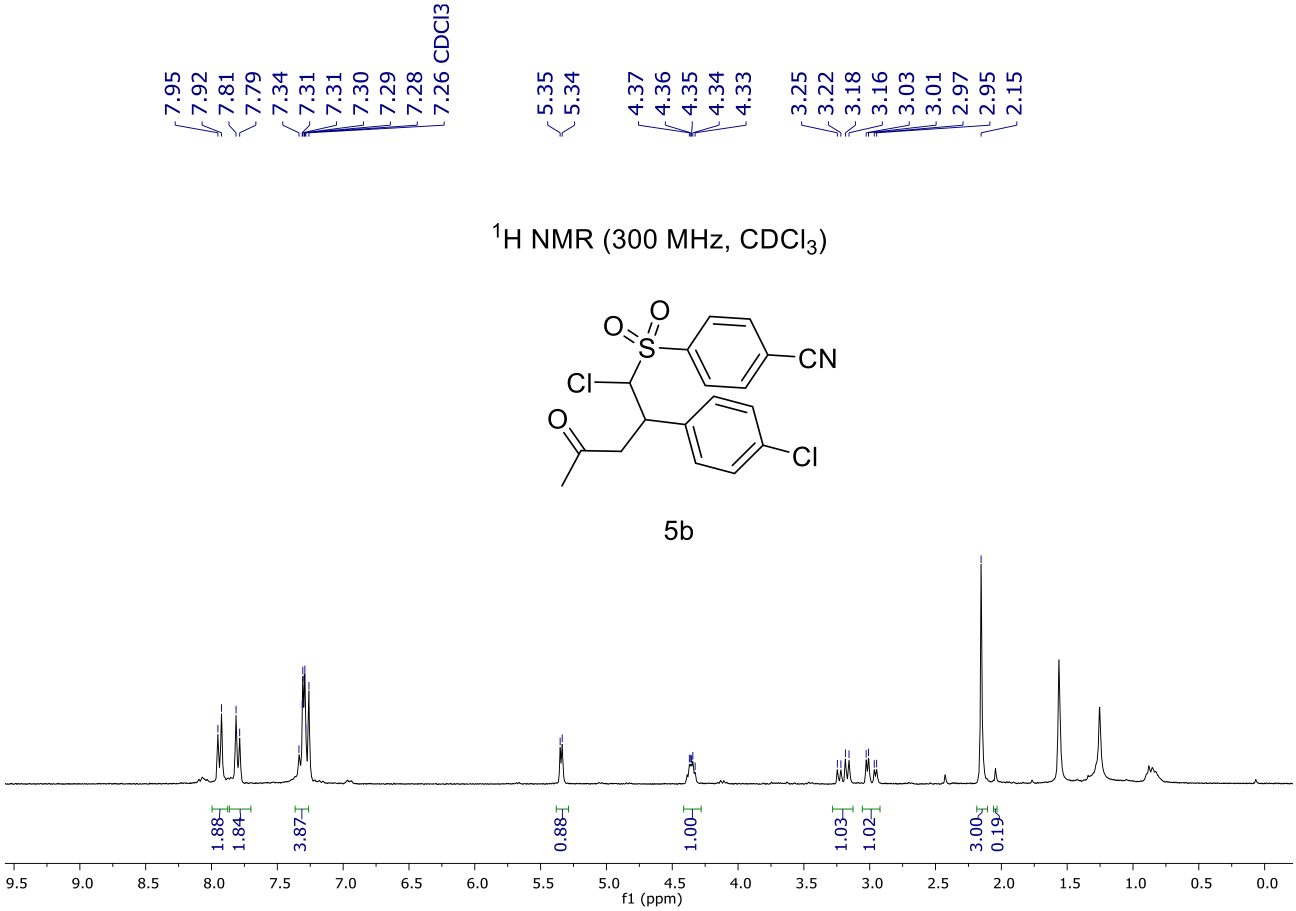
7.95
7.92
7.81
7.79
7.34
7.31
7.31
7.30
7.29
7.28
7.26 CDCl3
5.35
5.34
4.37
4.36
4.35
4.34
4.33
3.25
3.22
3.18
3.16
3.03
3.01
2.97
2.95
2.15
1H NMR (300 MHz, CDCl3)
CN
Cl
5b
1.88
1.84
3.87
0.88
1.00
1.03
1.02
3.00
0.19
f1 (ppm)

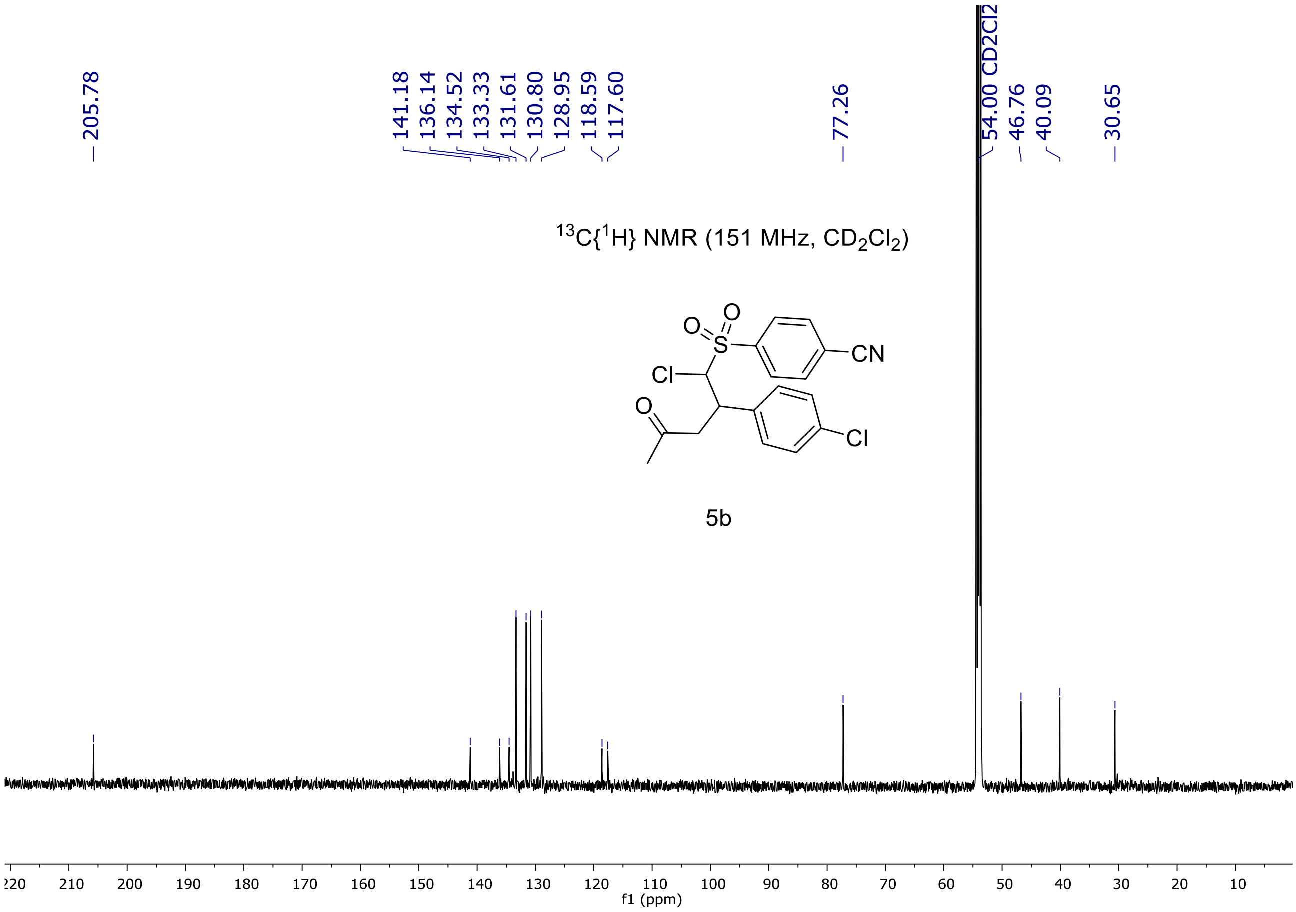
205.78
141.18
136.14
134.52
133.33
131.61
130.80
128.95
118.59
117.60
77.26
54.00 CD2Cl2
46.76
40.09
30.65
13C{1H} NMR (151 MHz, CD2Cl2)
CN
Cl
5b
f1 (ppm)

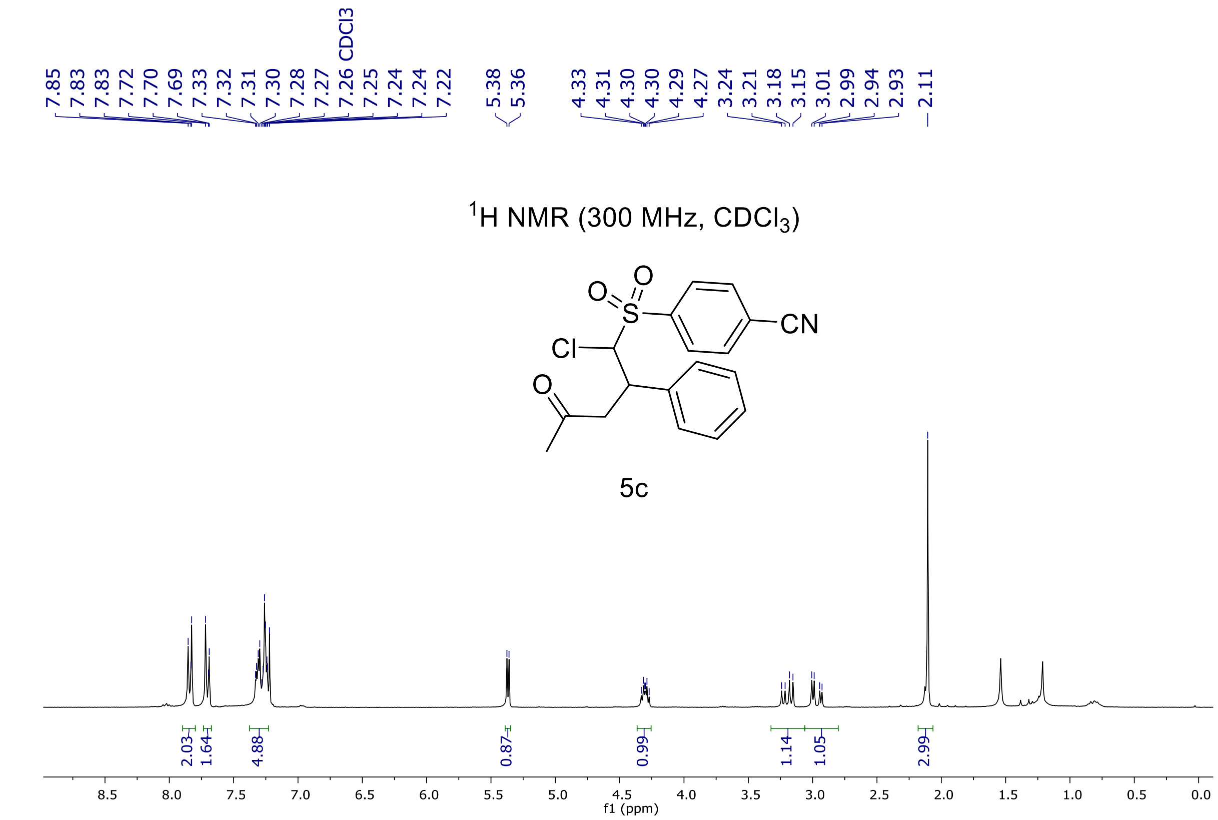
7.85
7.83
7.83
7.72
7.70
7.69
7.33
7.32
7.31
7.30
7.28
7.27
7.26 CDCl3
7.25
7.24
7.24
7.22
5.38
5.36
4.33
4.31
4.30
4.30
4.29
4.27
3.24
3.21
3.18
3.15
3.01
2.99
2.94
2.93
2.11
¹H NMR (300 MHz, CDCl₃)
O
O
S
CN
Cl
O
5c
2.03
1.64
4.88
0.87
0.99
1.14
1.05
2.99
8.5
8.0
7.5
7.0
6.5
6.0
5.5
5.0
4.5
4.0
3.5
3.0
2.5
2.0
1.5
1.0
0.5
0.0
f1 (ppm)

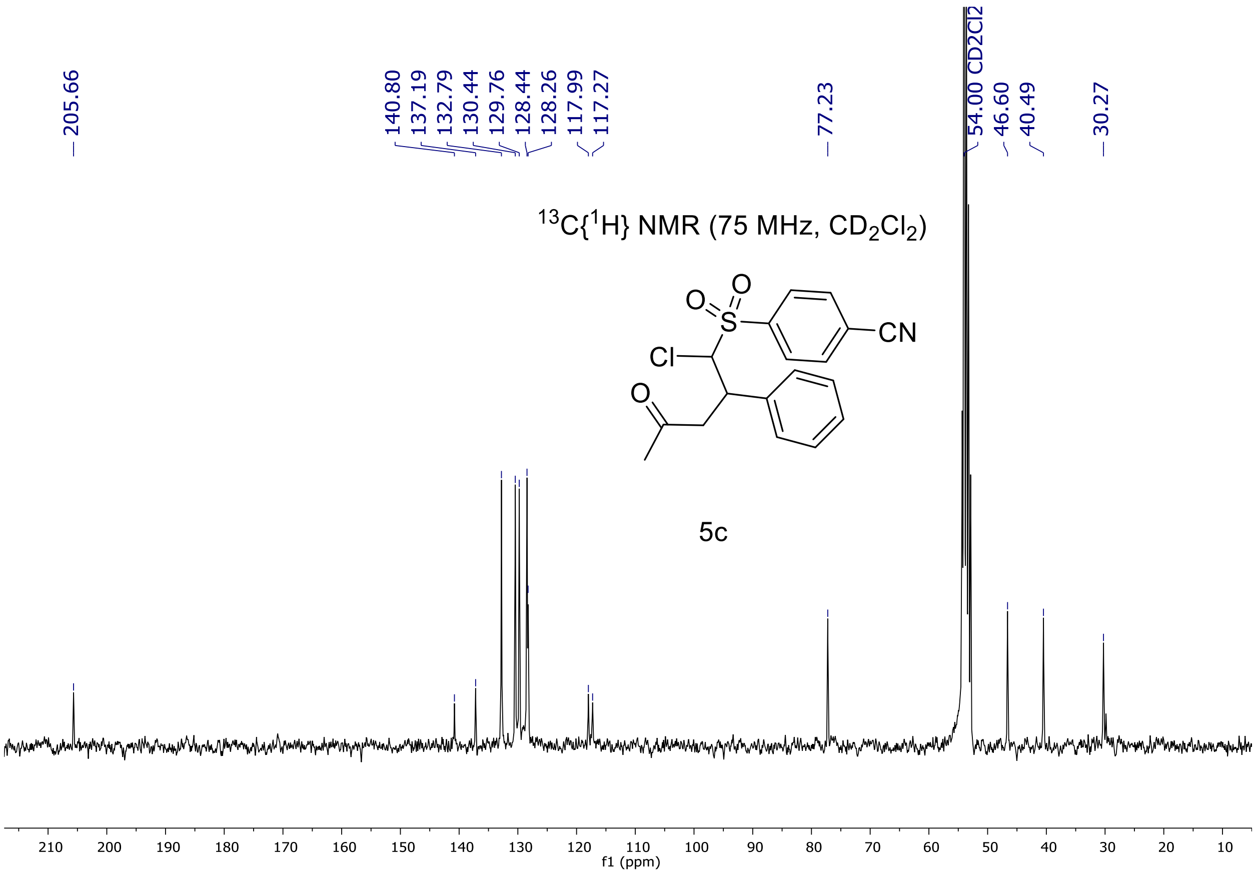
205.66
140.80
137.19
132.79
130.44
129.76
128.44
128.26
117.99
117.27
77.23
54.00 CD2Cl2
46.60
40.49
30.27
¹³C{¹H} NMR (75 MHz, CD₂Cl₂)
O
O
S
CN
Cl
O
5c
210
200
190
180
170
160
150
140
130
120
110
100
90
80
70
60
50
40
30
20
10
f1 (ppm)

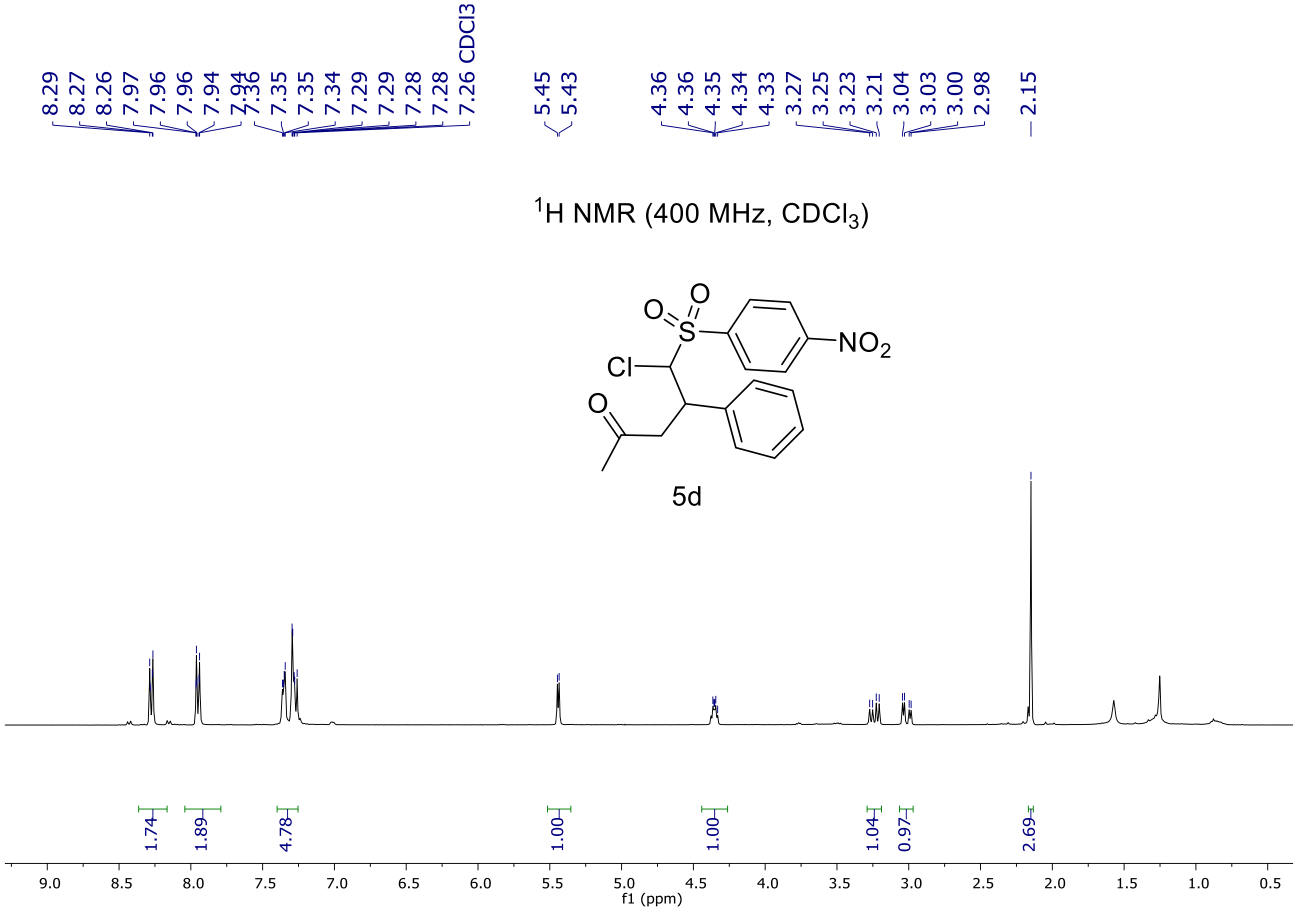
8.29
8.27
8.26
7.97
7.96
7.96
7.94
7.94
7.36
7.35
7.35
7.34
7.29
7.29
7.28
7.28
7.26 CDCl3
5.45
5.43
4.36
4.36
4.35
4.34
4.33
3.27
3.25
3.23
3.21
3.04
3.03
3.00
2.98
2.15
^{1}H NMR (400 MHz, CDCl$_3$)
O
O
S
NO$_2$
Cl
O
5d
1.74
1.89
4.78
1.00
1.00
1.04
0.97
2.69
9.0 8.5 8.0 7.5 7.0 6.5 6.0 5.5 5.0 4.5 4.0 3.5 3.0 2.5 2.0 1.5 1.0 0.5
f1 (ppm)

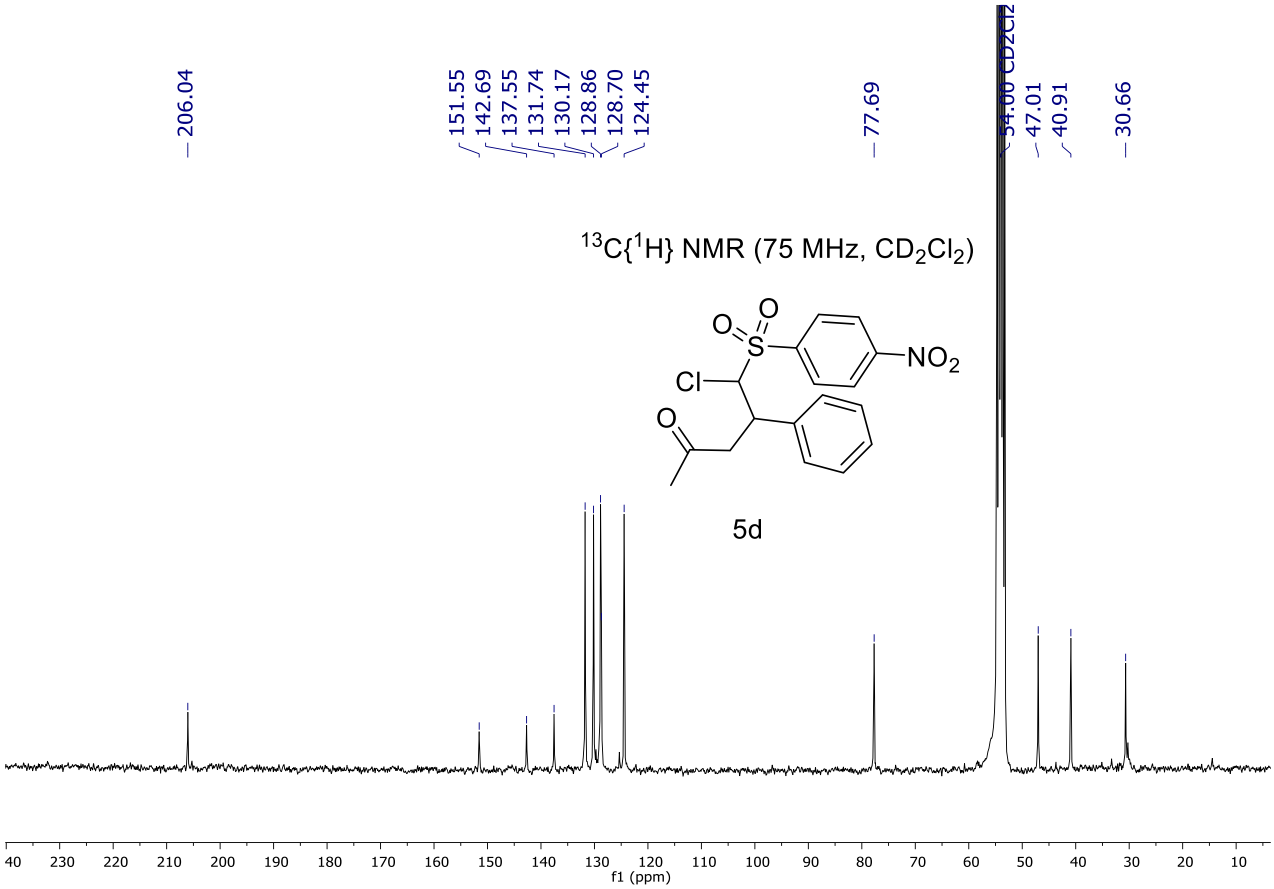
206.04
151.55
142.69
137.55
131.74
130.17
128.86
128.70
124.45
77.69
54.00 CD2Cl2
47.01
40.91
30.66
^{13}C{^{1}H} NMR (75 MHz, CD$_2$Cl$_2$)
O
O
S
NO$_2$
Cl
O
5d
40 230 220 210 200 190 180 170 160 150 140 130 120 110 100 90 80 70 60 50 40 30 20 10
f1 (ppm)

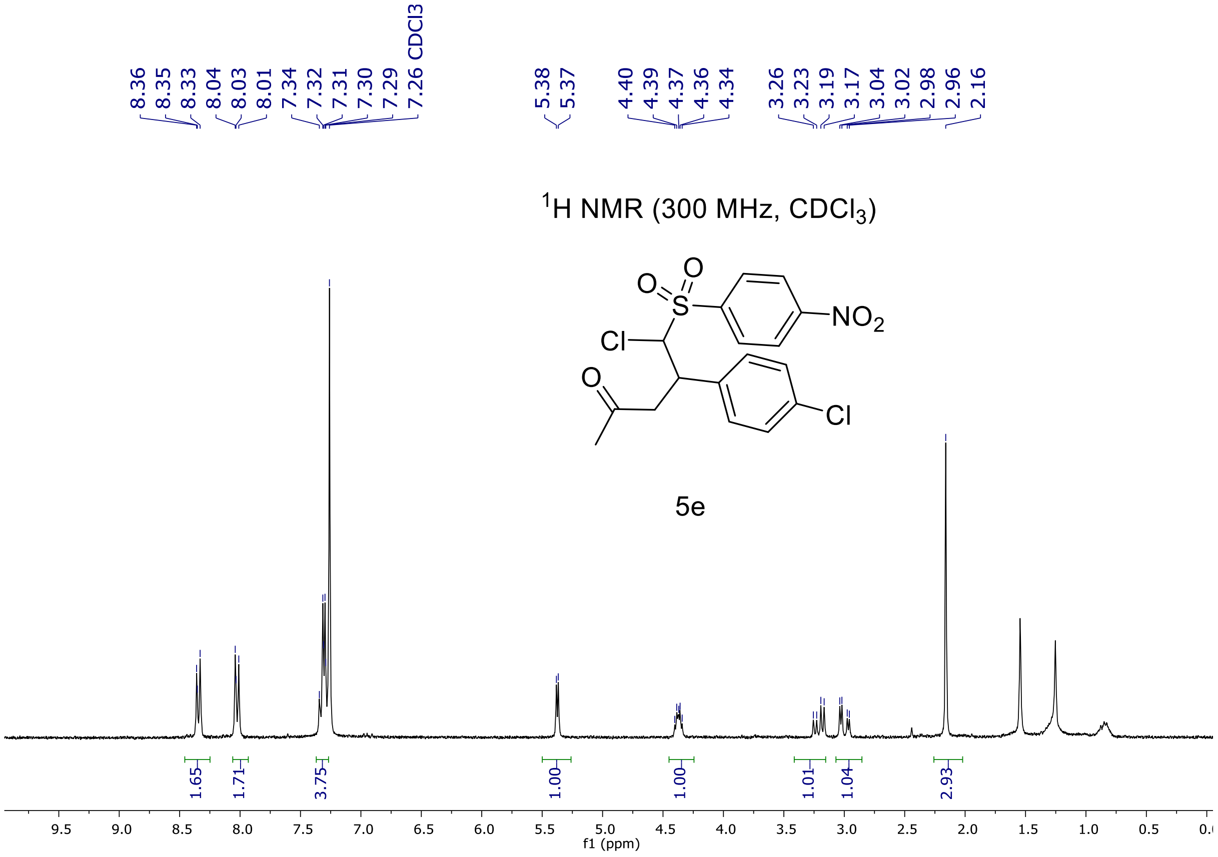
8.36
8.35
8.33
8.04
8.03
8.01
7.34
7.32
7.31
7.30
7.29
7.26 CDCl3
5.38
5.37
4.40
4.39
4.37
4.36
4.34
3.26
3.23
3.19
3.17
3.04
3.02
2.98
2.96
2.16
1H NMR (300 MHz, CDCl3)
O
O
S
NO2
Cl
O
Cl
5e
1.65
1.71
3.75
1.00
1.00
1.01
1.04
2.93
f1 (ppm)

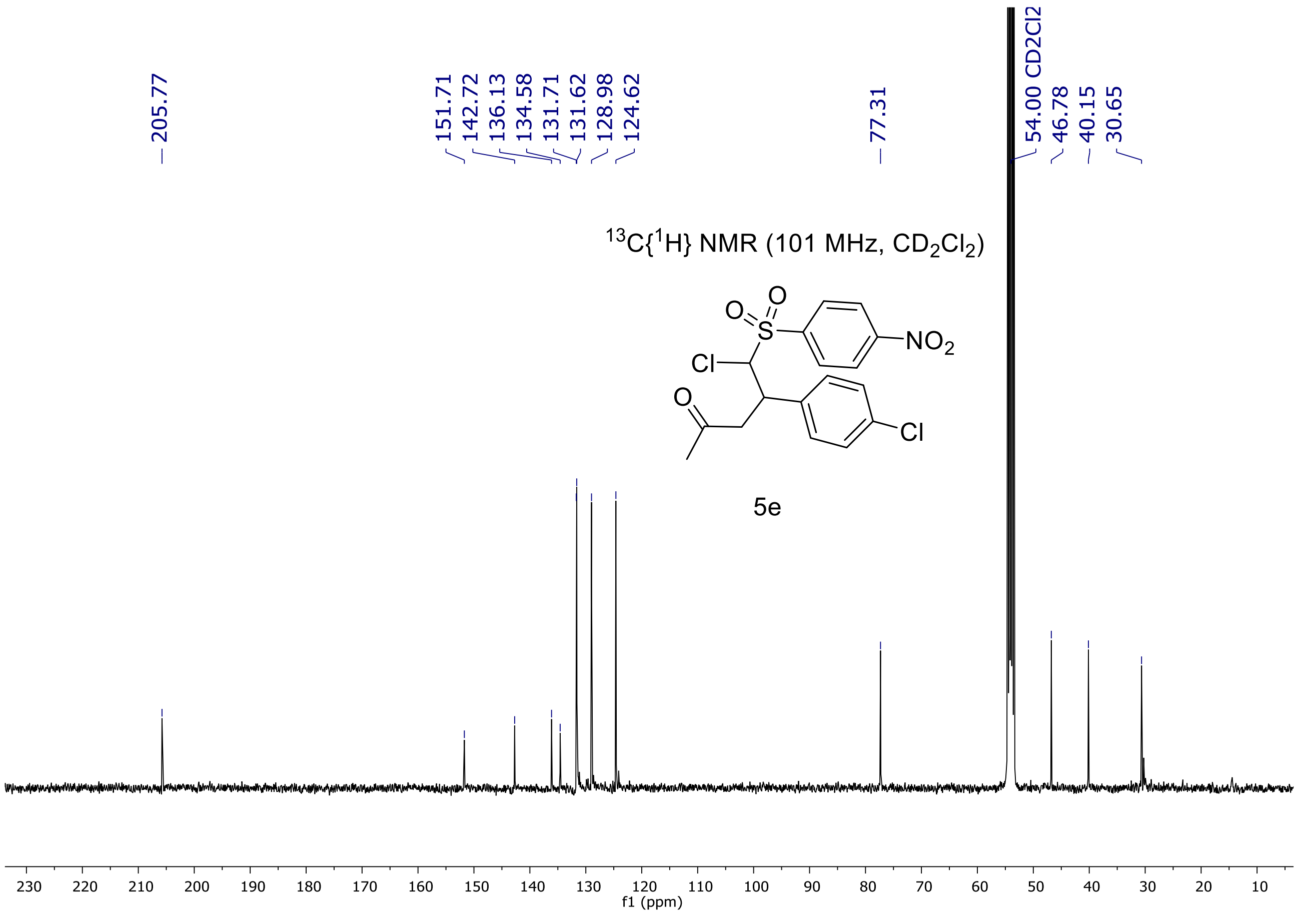
205.77
151.71
142.72
136.13
134.58
131.71
131.62
128.98
124.62
77.31
54.00 CD2Cl2
46.78
40.15
30.65
13C{1H} NMR (101 MHz, CD2Cl2)
O
O
S
NO2
Cl
O
Cl
5e
f1 (ppm)

IR spectra of some compounds

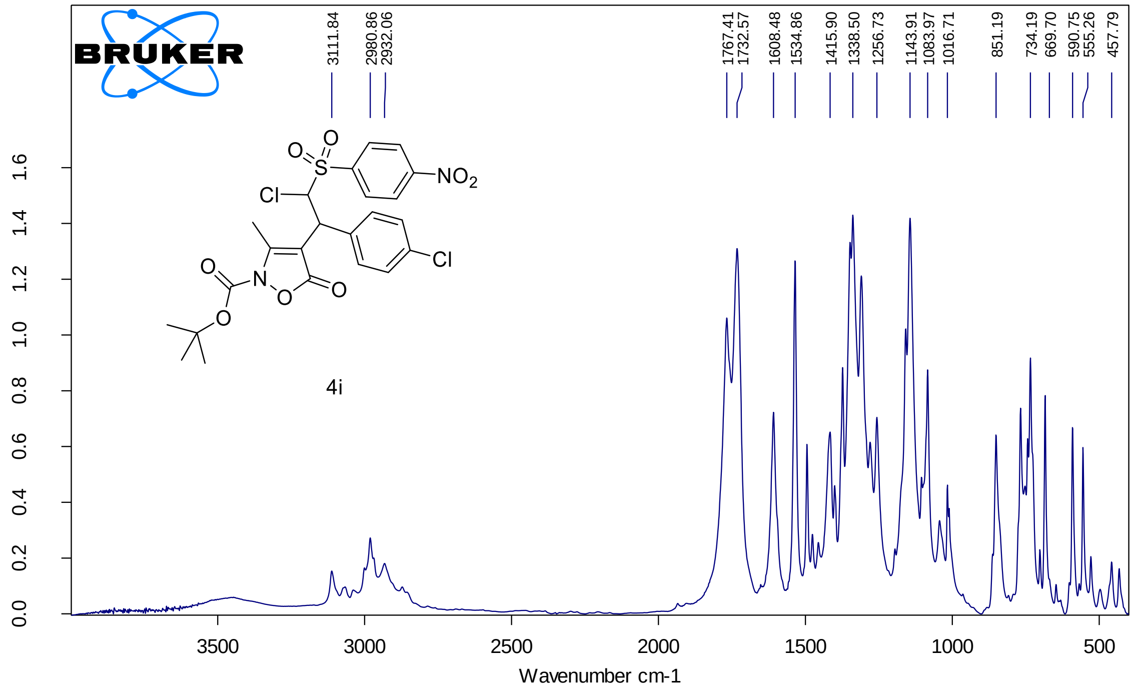

C:\Users\Opty\Desktop\Massa\AMAC150.0 Sample name KBr	27/01/2022

Page 1 of 1

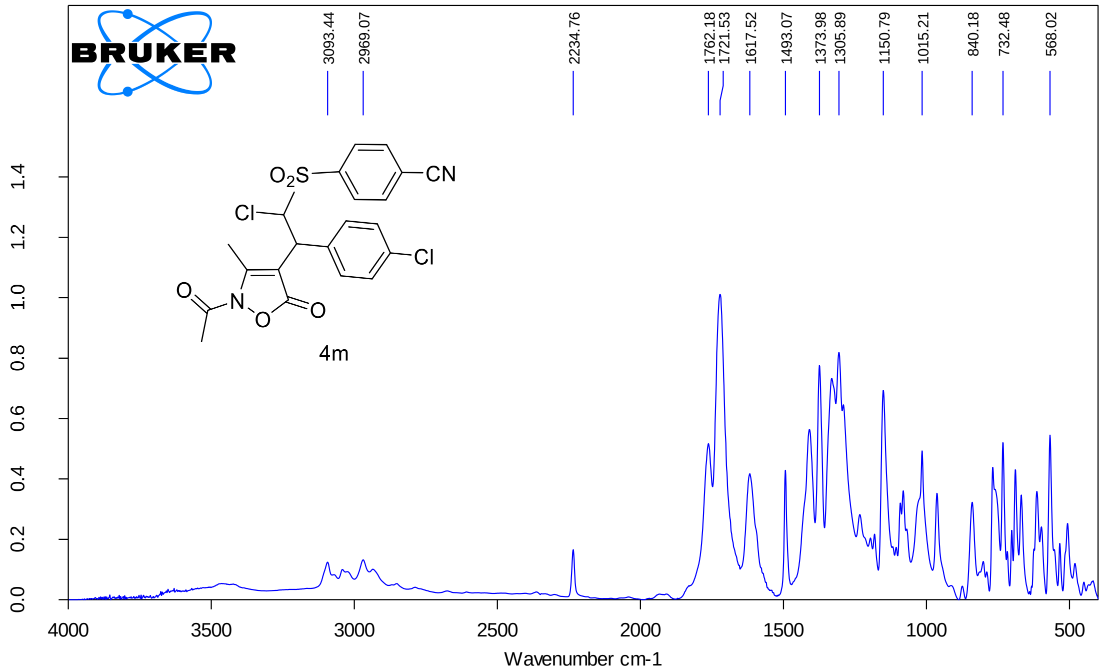

C:\Users\Opty\Desktop\Massa\AMAC114.0 Sample name KBr	27/01/2022

Page 1 of 1

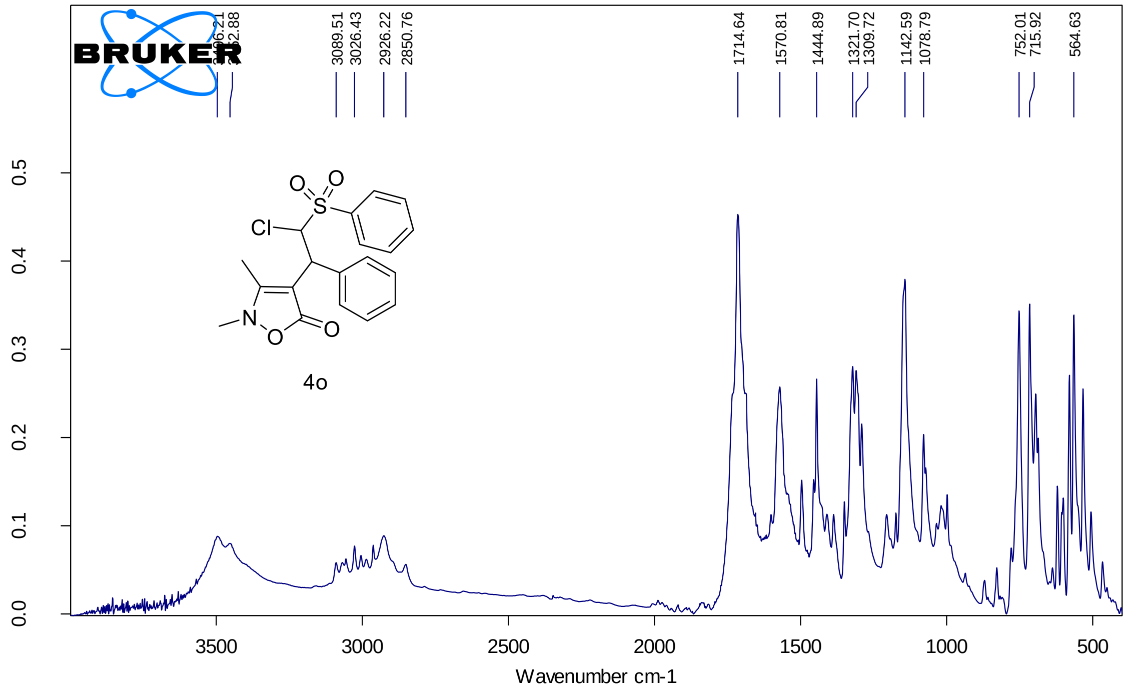

C:\Users\Opty\Desktop\Massa\AMAC119.0	Sample name	KBr	27/01/2022

Page 1 of 1

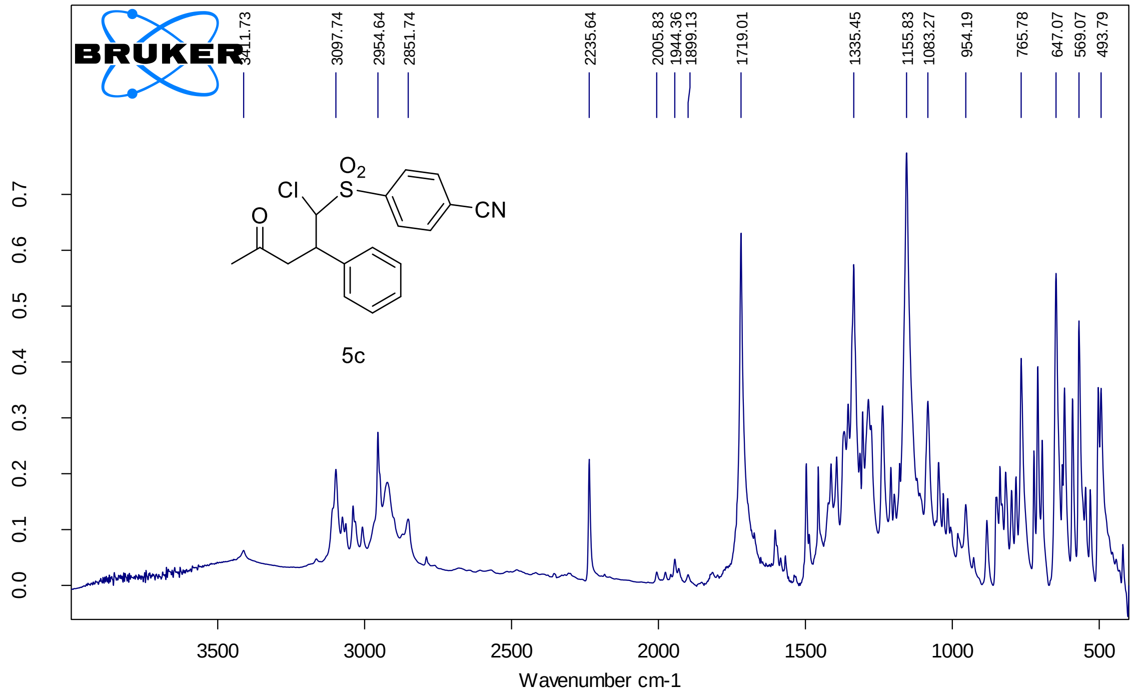

C:\Users\Opty\Desktop\Massa\AMAC141.0	Sample name	KBr	27/01/2022

Page 1 of 1
